# Accurate and efficient detection of gene fusions from RNA sequencing data

**DOI:** 10.1101/gr.257246.119

**Published:** 2021-03

**Authors:** Sebastian Uhrig, Julia Ellermann, Tatjana Walther, Pauline Burkhardt, Martina Fröhlich, Barbara Hutter, Umut H. Toprak, Olaf Neumann, Albrecht Stenzinger, Claudia Scholl, Stefan Fröhling, Benedikt Brors

**Affiliations:** 1 Division of Applied Bioinformatics, German Cancer Research Center (DKFZ) and National Center for Tumor Diseases (NCT) Heidelberg, 69120 Heidelberg, Germany;; 2 Computational Oncology Group, Molecular Diagnostics Program at the NCT and DKFZ, 69120 Heidelberg, Germany;; 3 German Cancer Consortium (DKTK), 69120 Heidelberg, Germany;; 4 Faculty of Biosciences, Heidelberg University, 69120 Heidelberg, Germany;; 5 Division of Translational Medical Oncology, NCT Heidelberg and DKFZ, 69120 Heidelberg, Germany;; 6 Division of Neuroblastoma Genomics, DKFZ, 69120 Heidelberg, Germany;; 7 Institute of Pathology, University Hospital Heidelberg, 69120 Heidelberg, Germany;; 8 German Center for Lung Research (DZL), Heidelberg site, 69120 Heidelberg, Germany;; 9 Division of Applied Functional Genomics, DKFZ and NCT Heidelberg, 69120 Heidelberg, Germany;; 10 NCT Molecular Diagnostics Program, NCT Heidelberg and DKFZ, 69120 Heidelberg, Germany

## Abstract

The identification of gene fusions from RNA sequencing data is a routine task in cancer research and precision oncology. However, despite the availability of many computational tools, fusion detection remains challenging. Existing methods suffer from poor prediction accuracy and are computationally demanding. We developed Arriba, a novel fusion detection algorithm with high sensitivity and short runtime. When applied to a large collection of published pancreatic cancer samples (*n* = 803), Arriba identified a variety of driver fusions, many of which affected druggable proteins, including ALK, BRAF, FGFR2, NRG1, NTRK1, NTRK3, RET, and ROS1. The fusions were significantly associated with *KRAS* wild-type tumors and involved proteins stimulating the MAPK signaling pathway, suggesting that they substitute for activating mutations in *KRAS*. In addition, we confirmed the transforming potential of two novel fusions, *RRBP1*-*RAF1* and *RASGRP1*-*ATP1A1*, in cellular assays. These results show Arriba's utility in both basic cancer research and clinical translation.

Gene fusions play a major role as oncogenic drivers in many cancer types. This insight has immediate consequences for the treatment of patients, because many gene fusions can be addressed therapeutically with targeted drugs (Schram et al. 2017). The most prominent examples are fusions between *BCR* and *ABL1* in chronic myeloid leukemia and acute lymphoblastic leukemia, which can be treated effectively using imatinib and related drugs (An et al. 2010). More recently, the U.S. Food and Drug Administration (FDA) granted accelerated approval for the treatment of solid tumors harboring *NTRK* fusions with larotrectinib after showing antitumor activity in three multicenter trials (Drilon et al. 2018). For this reason, a routine task in genomics-guided precision oncology is to search for evidence of gene fusions in RNA sequencing (RNA-seq) data.

Although a variety of computational tools for the detection of gene fusions have been developed over the years, there is still no gold standard. The reliable prediction of gene fusions from short-read RNA-seq has proven to be difficult owing to a myriad of artifacts being introduced during library preparation and sequence alignment. To keep the number of false-positive predictions low, the algorithms implement stringent filters, with the undesired side effect that occasionally driver fusions are discarded and events with subtle evidence in RNA-seq data are lost entirely. The current practice is to apply at least two tools and use the union or intersection of their predictions. This approach is computationally expensive, because each tool on its own typically takes many hours or even days to run. With high-throughput sequencing (HTS) technology becoming more common in clinical practice to identify targetable alterations, the demand for algorithms that are both accurate and efficient grows. Inaccurate predictions complicate the interpretation of HTS-based results, and the time-critical operation of a precision oncology trial does not tolerate slow computational pipelines, because the overall workflow allocates only a few days for bioinformatics processing (Roychowdhury et al. 2011; Worst et al. 2016).

We developed Arriba, a fusion detection algorithm specifically designed to meet the demanding requirements of HTS-assisted precision oncology. Owing to a highly optimized workflow, it can process contemporary RNA-seq samples in less than an hour. Sophisticated filters detect fusions even under unfavorable conditions, such as low sample purity. In addition, Arriba is capable of detecting aberrant transcripts that are not called by most fusion detection methods but may be clinically relevant. This includes tumor suppressor genes that are occasionally inactivated by rearrangements within the gene or by translocations to introns or intergenic regions. Even though the same technical approach can be applied to detect such transcripts, most available fusion detection tools do not report them. As a consequence, clinically relevant aberrations may be overlooked. Arriba improves over existing methods in that it can find intragenic inversions/duplications and translocations to introns/intergenic regions.

Based on novel gene fusions involving *NRG1* that we recently discovered in a series of patients with *KRAS* wild-type pancreatic tumors (Heining et al. 2018) in the context of NCT/DKTK MASTER (Horak et al. 2017), a HTS-guided precision oncology program, we applied Arriba to further explore the relevance of gene fusions in pancreatic cancer. In particular, we investigated the prevalence of druggable fusions, because there are few targeted treatment options for pancreatic cancer patients, and despite recent improvements in conventional and targeted therapies (Conroy et al. 2018; Golan et al. 2019), the 5-yr overall survival rate is <10%.

## Results

We compared the performance of Arriba v1.0.0 against six commonly used fusion detection algorithms (defuse v0.8.1 [McPherson et al. 2011], FusionCatcher v1.00 [Nicorici et al. 2014], InFusion v0.8 [Okonechnikov et al. 2016], PRADA v1.2 [Torres-García et al. 2014], SOAPfuse v1.27 [Jia et al. 2013], STAR-Fusion v1.4.0 [Haas et al. 2019]) with respect to speed and accuracy.

### Accuracy benchmarks

To show Arriba's robust performance across diverse types of input data, we assessed its accuracy on four types of peer-reviewed benchmark data sets (Supplemental Tables S1, S2):

We used in silico–generated fusion transcripts from Jia et al. (2013), who simulated 150 fusion transcripts and merged them into an RNA-seq sample from benign tissue (H1 human embryonic stem cells), serving as background expression. To measure the sensitivity of a method as a function of the expression level of a fusion transcript, nine different expression levels were simulated, ranging from five- to 200-fold.

Next, we took RNA-seq samples from Tembe et al. (2014), who used a semisynthetic approach to benchmark fusion detection algorithms. The investigators spiked in synthetic RNA molecules into RNA libraries of the melanoma cell line COLO-829. The synthetic RNA molecules mimic the transcript sequences of nine oncogenic fusions found in a variety of cancer types. They were spiked into 20 replicates of RNA libraries at 10 different concentrations ranging from 10^−8.57^ pMol to 10^−3.47^ pMol. In addition, one endogenous fusion of the COLO-829 cell line was confirmed via orthogonal validation.

To measure the performance on real data, we ran the tools on eight samples from four cell lines (Edgren et al. 2011; The ENCODE Project Consortium 2012; Lin et al. 2015), including the breast cancer cell line MCF-7, a well-studied cancer cell line with a highly rearranged genome and many gene fusions validated via orthogonal methods. We used the list of validated fusions compiled by Davidson et al. (2015), which comprises 69 distinct pairs of fusion genes. Because this list is biased toward fusions that were detected by previous methods and potentially lacks events that can be detected by newer, more sensitive methods, we also considered a prediction to be true if its breakpoints were close to the breakpoints of a structural variant identified in a whole-genome sequencing (WGS) sample of the MCF-7 cell line (Li et al. 2016). Furthermore, we subjected the top predictions of each tool to experimental validation if they were confirmed neither by previous validation tests nor by structural variants (Supplemental Table S3).

Lastly, we applied the tools to patient data from the ICGC early-onset prostate cancer cohort (ICGC-EOPC) and the TCGA diffuse large B-cell lymphoma cohort (TCGA-DLBC). Early-onset prostate cancer is characterized by a high prevalence of *TMPRSS2-ERG* fusions (Gerhauser et al. 2018). Fusions involving the immunoglobulin (IG) loci and one of *BCL2*, *BCL6*, or *MYC* are hallmark aberrations of diffuse large B-cell lymphoma (Schmitz et al. 2018) and are hard to detect owing to the poor mappability of the IG loci. We measured the recall rate of these diagnostically relevant fusions to get an impression of how well each fusion detection tool would be suited for a clinically oriented setting.

**Figure 1.**
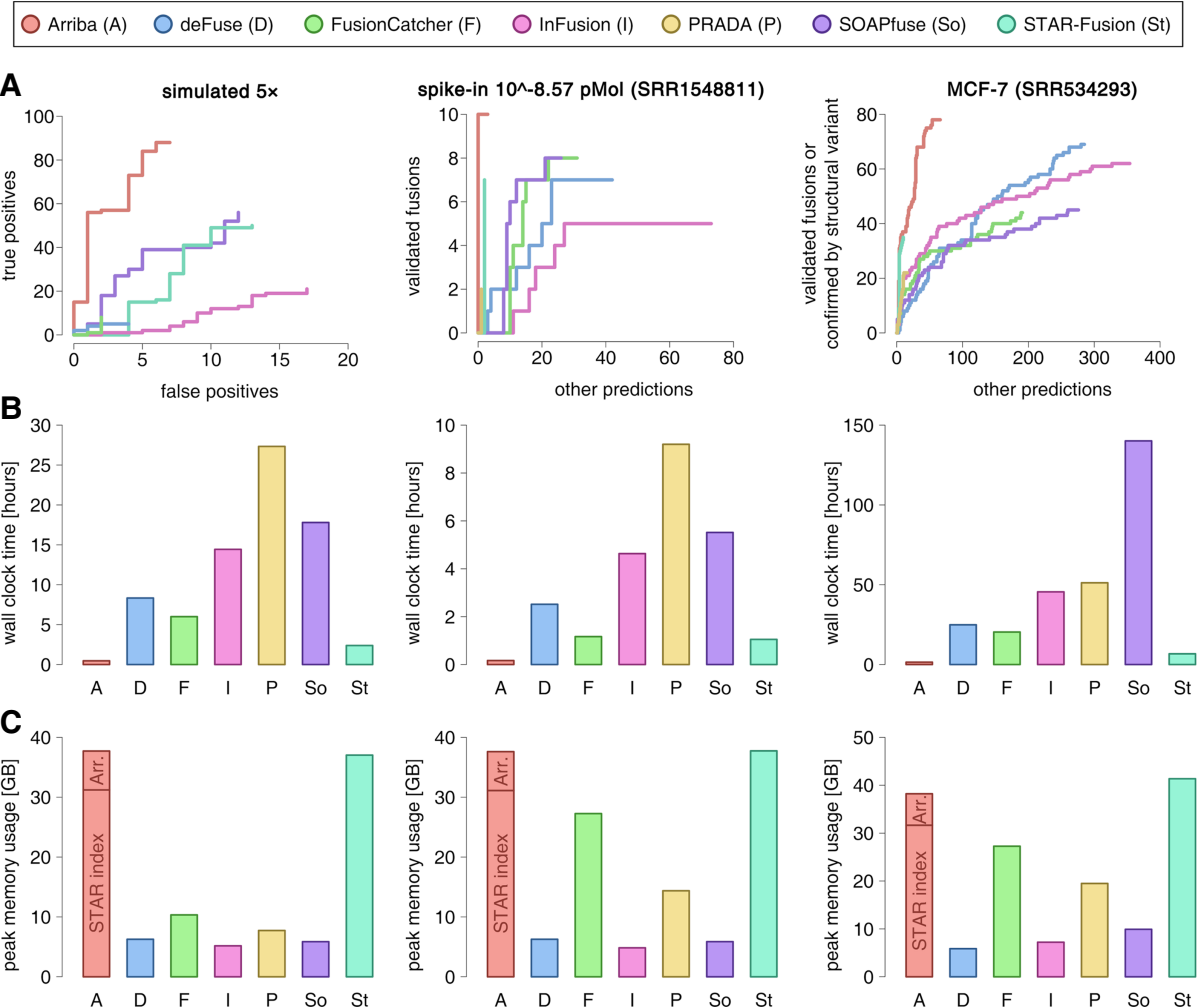
Benchmark of Arriba versus alternative methods. (*A*) Accuracy benchmarks. The figure shows samples from three types of benchmark data set: simulated fusions, spike-ins of synthetic fusions, and fusions described in the MCF-7 breast cancer cell line. The sensitivity/specificity trade-off is depicted using receiver operating characteristic (ROC)–like curves. The vertical axis indicates the number of true positives; the horizontal axis indicates the number of false positives (simulated data set) or nonvalidated predictions (spike-in and MCF-7 data sets). (*B*) Runtimes. (*C*) Peak memory consumption in gigabytes (GB). The aligner (STAR) and its index accounted for 31 GB of the memory footprint of Arriba's workflow. Approximately 7 GB were consumed by Arriba (Arr.) itself.

**Figure 2.**
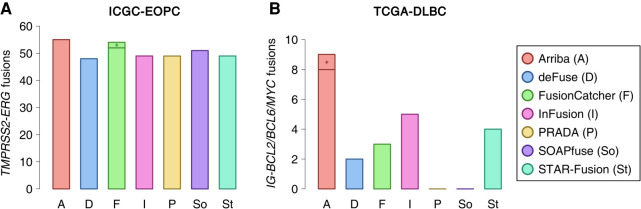
Recall of hallmark gene fusions in prostate cancer and diffuse large B-cell lymphoma. To measure the performance of Arriba and alternative methods on real patient data, we counted the number of hallmark gene fusions detected by each method in two cohorts. Fractions marked with an asterisk were only detected when a list of known/expected fusions was provided. (*A*) *TMPRSS2-ERG* fusions in the ICGC-EOPC cohort. (*B*) *IG-BCL2/BCL6/MYC* fusions in the TCGA-DLBC cohort.

Figure 1A uses receiver operating characteristic (ROC)–like curves to visualize the enrichment of validated predictions versus nonvalidated predictions as a function of the rank of a prediction in the output file of a tool. Arriba's superior performance becomes particularly evident when fusion transcripts are supported by few reads. The figure shows the accuracy of the evaluated methods on the samples with the lowest concentrations of fusion transcripts (fivefold for simulated fusions, 10^−8.57^ pMol for spike-in fusions). At higher concentrations, all methods achieve similar accuracy (Supplemental Figs. S1, S2; Supplemental Table S4). For a fair comparison, Figure 1A only considers gene-to-gene fusions, because not all tools are able to identify fusions with intergenic breakpoints. Supplemental Figure S3 considers only fusions with intergenic breakpoints and compares the performance of those methods that are capable of detecting such rearrangements. In both cases, Arriba showed favorable accuracy:

In all four types of benchmark data sets, Arriba showed the highest sensitivity: It rediscovered 88 of the 150 simulated fusions at the fivefold expression level, all of the synthetic fusions, 78 fusions in the MCF-7 cell line that had been validated or were confirmed by a structural variant, 55 *TMPRSS2-ERG* fusions in the ICGC-EOPC cohort (Fig. 2A; Supplemental Table S2A), and eight *IG-BCL2/BCL6/MYC* translocations in the TGCA-DLBC cohort (Fig. 2B; Supplemental Table S2B). This corresponds to a surplus in sensitivity of 57%, 25%, 13%, 6%, and 60%, respectively, compared with the next best method (SOAPfuse, FusionCatcher/SOAPfuse, deFuse, FusionCatcher, and InFusion, respectively). The most frequent reason that Arriba failed to report an expected event was an insufficient number of supporting reads; that is, STAR aligned between zero and two chimeric reads, which is below/at the detection limit of Arriba. Only three of the simulated fusions were erroneously classified as alignment artifacts.

If desired, Arriba and FusionCatcher can be run with a list of expected/known fusions. The tools then apply sensitive parameters for the listed fusion candidates, which is useful when high sensitivity is desirable, such as in a clinical setting. We processed the ICGC-EOPC and TCGA-DLBC cohorts anew with Arriba and FusionCatcher, this time supported by a list of known fusions. Arriba did not detect any additional *TMPRSS2-ERG* fusions in the ICGC-EOPC cohort; FusionCatcher detected two more ones but even then, in total, still fewer than Arriba. In the TCGA-DLBC cohort, Arriba identified one additional *IG-BCL2* fusion, thus expanding its sensitivity advantage over the second-best method to 80%, whereas FusionCatcher's sensitivity remained unchanged. Another common approach to improve sensitivity in a clinically oriented workflow is to run multiple complementary fusion detection tools. Even when all alternative methods were combined, the detection rate improved only marginally over that of standalone Arriba: Apart from a single *TMPRSS2-ERG* fusion in patient ICGC_PCA032, Arriba subsumed all patients reported as fusion-positive by alternative methods (Supplemental Table S2).

In terms of specificity, Arriba can compete with state-of-the-art methods. Of the 98 events predicted from the data set simulating fusions at fivefold expression, only 10 were false positives, which is the smallest fraction of incorrect predictions among all tested methods. At higher simulated expression levels, Arriba achieves average specificity. The number of false-positive predictions from the COLO-829 and MCF-7 samples cannot be determined precisely, because not all endogenous fusions are known. However, qualitative conclusions on the specificity of the evaluated tools can be derived from the rankings of their predictions. An enrichment of validated events among the top-ranking predictions indicates that a tool is of high practical utility, because validating predictions in this order results in a high fraction of successful validations per spent budget. Even at the lowest concentration of spike-in fusion transcripts, Arriba's top-ranking predictions were near-optimally enriched for true positives. Other tools achieved the same level of enrichment only at higher concentrations. The MCF-7 data set contains a mixture of high- and low-expressed fusions. All methods showed strong enrichment among the top-ranking predictions, which mostly consist of highly expressed fusions supported by many chimeric reads in the RNA-seq data. The specificities of the methods diverged for borderline detectable events, which are reported toward the end of the output files. Here, Arriba outperformed all other methods.

Arriba assigns one of three confidence classes to its predictions: low, medium, and high. Users can choose their preferred balance between sensitivity and specificity by selecting for events above a certain confidence class. Fifty-six of 85 (66%) of the high-confidence predictions, 13 of 25 (52%) of the medium-confidence predictions, and nine of 34 (26%) of the low-confidence predictions from the MCF-7 sample were correct. In view of the high false-positive rate of low-confidence predictions, we recommend that users treat these predictions with skepticism, unless additional evidence corroborates them, such as a correlating structural variant identified from a matched WGS sample. When searching for recurrently fused genes in a cohort, it is advisable to only consider medium- and high-confidence predictions; otherwise, the results will be enriched with false positives. But in situations in which sensitivity is crucial, low-confidence predictions can be of high value. For example, in HTS-based precision oncology, an increased number of false-positive predictions is acceptable as a trade-off for higher sensitivity if potentially relevant predictions are validated via orthogonal methods (Lier et al. 2018).

### Runtimes and memory consumption

We measured the runtimes of all tools on an AMD Opteron 6376 CPU using eight cores. The test samples comprised between 13 and 360 million reads (Supplemental Table S1). Arriba was the fastest in terms of both elapsed time (wall clock time) and CPU time, excelling the second-fastest tool, STAR-Fusion, by a factor of 5.6 on average (Fig. 1B; Supplemental Fig. S4). Despite having a workflow architecture similar to STAR-Fusion, Arriba's turn-around time was noticeably shorter, because STAR-Fusion takes longer for filtering of fusion candidates and aligns in two passes, whereas Arriba uses only a single pass. Arriba's workflow spent 91% of the runtime (95% of the CPU time) in the alignment step using STAR (Dobin et al. 2013). Because Arriba can extract candidate reads while the alignment is running, it extends the wall clock time only marginally: The post-alignment runtime was just 6.3 min in the worst case.

On average, the workflow based on Arriba consumed 38 GB of memory, which is 5.8 times more than the most memory-efficient tool, SOAPfuse (Fig. 1C; Supplemental Fig. S5). Approximately 31 GB of the memory footprint can be attributed to the suffix array index of STAR. Arriba itself consumed between 6.5 and 6.8 GB. By running Arriba sequentially rather than in parallel to STAR, the peak memory usage can be reduced to the size of STAR's index, at the expense of slightly longer runtimes.

### Using Arriba in practice

To accelerate routine tasks in gene fusion-related research, Arriba offers a number of useful features that go beyond mere prediction of fusion breakpoints. It provides the transcript sequence flanking the junction site, which helps with the design of primers for validation via Sanger sequencing. It also computes the peptide sequence resulting from the chimeric transcript, which can serve as a basis for the prediction of fusion-derived neoepitopes.

Furthermore, Arriba provides visualization tools to facilitate the interpretation of gene fusions. The R script *draw_fusions.R* yields publication-quality figures of Arriba's predictions. The figures depict the exons retained in the fusion gene as well as a coverage profile to reflect changes in expression of the exons before and after the breakpoints. Furthermore, the figures show the Pfam (El-Gebali et al. 2019) protein domains that are retained in the fusion. Because STAR stores chimeric alignments in SAM format, the alignments can be loaded into a genome browser, such as the Integrative Genomics Viewer (IGV) (Thorvaldsdottir et al. 2013), for closer inspection. Exploring the vicinity of the breakpoints interactively in a graphical viewer helps identify false-positive predictions arising from alignment artifacts and can give further insight into the architecture of complex rearrangements. Arriba provides a feature track with protein domains, which can be loaded into IGV alongside with the alignments to assess the functional implications of a fusion.

In addition to RNA-seq data, clinical research projects occasionally generate WGS data for each patient. Arriba's prediction accuracy can further be improved by supplying a list of structural variants obtained from WGS, which are incorporated into the filtering of equivocal predictions.

### Identification of oncogenic gene fusions in pancreatic cancer

The discovery of recurrent fusions involving *NRG1* in *KRAS* wild-type pancreatic tumors (Heining et al. 2018), as well as case reports of fusions involving *BRAF*, *PRKACA*, *NTRK1/3*, and *RET* (The Cancer Genome Atlas Research Network 2017; Drilon et al. 2018; Gao et al. 2018; Heining et al. 2018), prompted us to systematically screen for fusion genes in this cancer entity.

We collected RNA-seq samples from a total of 803 donors (Supplemental Table S5) across 18 published studies on pancreatic cancer (Barretina et al. 2012; Carugo et al. 2016; Diaferia et al. 2016; Kirby et al. 2016; Witkiewicz et al. 2016; Bhattacharyya et al. 2017; Horak et al. 2017; Nicolle et al. 2017; The Cancer Genome Atlas Research Network 2017; Aung et al. 2018; Lomberk et al. 2018; Bryant et al. 2019; Lin et al. 2019; Maurer et al. 2019). For 327 samples, matched WGS data were available. When Arriba predicted a gene fusion from the transcriptomic data, we checked for a correlating structural variant in the WGS data as confirmation for the validity of the prediction.

**Figure 3.**
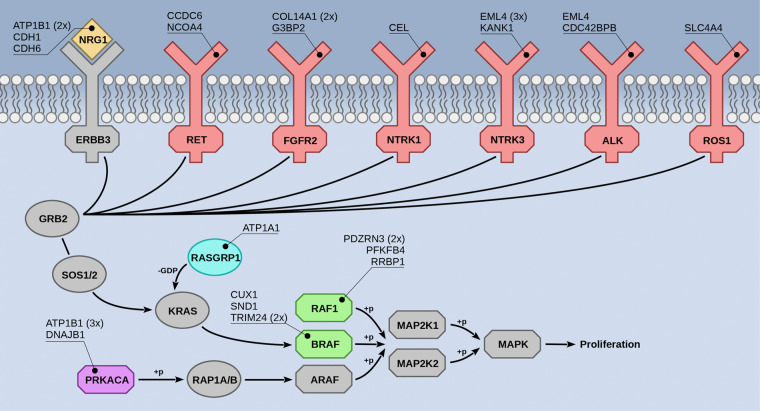
Gene fusions in pancreatic cancer. Overview of proteins in the MAPK signaling pathway found to be fused in pancreatic tumors. Colored proteins were fused to one of the genes listed in the callouts. Proteins shown in gray were not found to be fused. The frequencies of recurrent fusion partners are indicated in parentheses. The detailed structure of all fusions is depicted in Supplemental Figure S6.

We detected 30 potential driver fusions in the RNA-seq data (Fig. 3; Supplemental Fig. S6)—all of which were confirmed by structural variants in WGS data when available (Supplemental Fig. S7)—involving the following oncogenes: *BRAF* (4×), *NRG1* (4×), *NTRK3* (4×), *PRKACA* (4×), *RAF1* (4×), *FGFR2* (3×), *ALK* (2×), *RET* (2×), *NTRK1* (1×), *RASGRP1* (1×), and *ROS1* (1×). Some of the affected proteins are direct interaction partners of KRAS (RASGRP1, BRAF, RAF1), suggesting that the corresponding fusion proteins might activate the same pathway as oncogenic KRAS. Indeed, a statistical analysis interrogating if genes of any of the pathways annotated in the KEGG database (Kanehisa et al. 2017) were overrepresented in the set of 11 oncogenes listed above confirmed a significant association with the mitogen-activated protein kinase (MAPK) signaling pathway (KEGG ID hsa04010, overrepresentation enrichment analysis by WebGestalt [Wang et al. 2017], *P*-value = 7.8 × 10^−7^, Benjamini–Hochberg false-discovery rate = 8.4 × 10^−5^). Six of the oncogenes are contained in this pathway; the others activate the MAPK signaling via connected pathways (KEGG IDs hsa04012, hsa04722, hsa05200, hsa05223).

In 105 samples (13%) that were included into our analysis, we did not detect altered *KRAS*. The oncogenic fusions were significantly enriched in these samples (two-sided Fisher's exact test, *P*-value = 9.9 × 10^−21^), with only four fusions (2× *FGFR2-COL14A1*, 1× *DNAJB1-PRKACA*, 1× *KANK1-NTRK3*) being found in *KRAS* mutant tumors (Supplemental Table S5). In 79 tumors, we detected neither a *KRAS* mutation nor a driving fusion. To rule out the possibility that we overlooked fusions owing to short-comings of Arriba, we also ran the other fusion detection tools on the cohort, but none of them reported driving fusions beyond Arriba's set. In fact, Arriba showed the highest sensitivity, detecting between three and 11 more driving fusions than the other methods, thus confirming the results of our benchmarks.

Some of the identified pancreatic gene fusions have been reported in the context of other cancer types: One case carried a *DNAJB1-PRKACA* fusion, which has been described in fibrolamellar hepatocellular carcinoma (Honeyman et al. 2014). Three cases harbored fusions between *EML4* and *NTRK3*, first observed in infantile fibrosarcoma (Tannenbaum-Dvir et al. 2015). Three cases were characterized by *NCOA4-RET*, *CCDC6-RET*, and *SND1-BRAF* fusions, which are more commonly seen in papillary thyroid carcinoma (Gao et al. 2018). In a pancreatic cancer cell line, we found a fusion between *EML4* and *ALK*, as known from non-small-cell lung cancer (Gao et al. 2018). *TRIM24-BRAF* and *CUX1-BRAF* fusions have previously been reported in melanoma (Ross et al. 2016). Fusions between *KANK1* and *NTRK3* have been observed in *BRAF* wild-type renal metanephric adenoma (Catic et al. 2017). The other fusions had not been described before but resembled well-known oncogenic fusions with regard to their structure (Supplemental Fig. S6): The oncogene constituted the 3′ end of the fusion and comprised the same exons as seen in established oncogenic fusions, but the 5′ gene of the fusion had not been observed as a recurrent partner. For example, we identified a fusion with *NTRK1*, which retained the kinase domain of *NTRK1*, but instead of the more common fusion partner *TPM3* (Drilon et al. 2018), the gene *CEL* served as 5′ fusion partner. Two of the rearrangements affecting *RAF1* were structurally similar to *RAF1* fusions known from cutaneous melanoma (Gao et al. 2018). And the fusions involving *ALK* and *ROS1* preserved the tyrosine kinase domains of these genes as seen in lung adenocarcinoma (Gao et al. 2018).

#### Functional validation of two novel fusion genes

**Figure 4.**
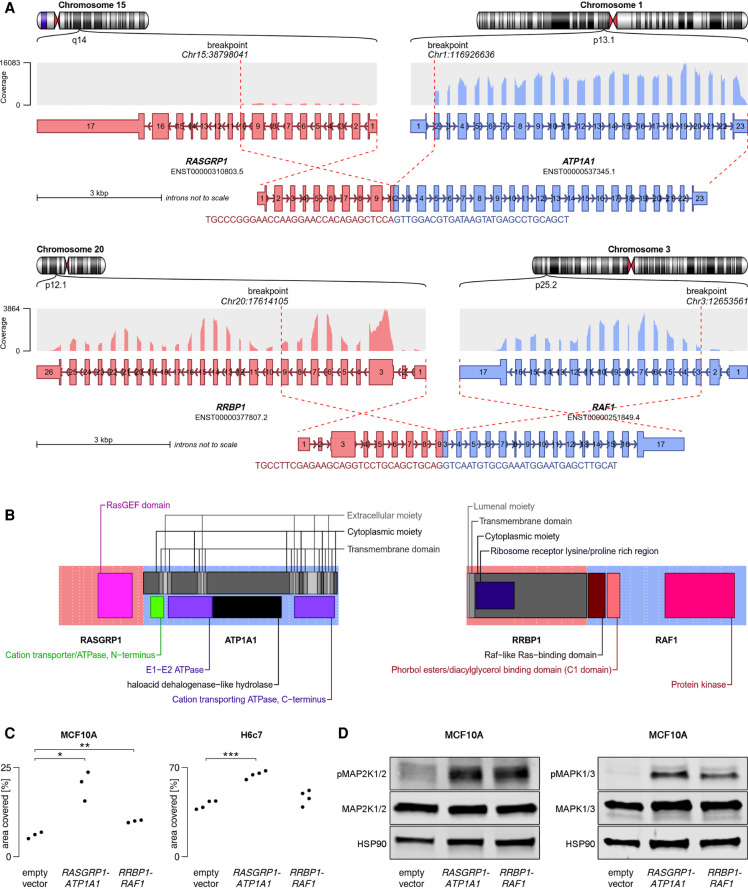
Structural and functional characteristics of *RRBP1-RAF1* and *RASGRP1-ATP1A1*. (*A*) Structure of the fusion transcripts. (*B*) Protein domains retained in the fusion proteins and topology. Near full-length RAF1 was found to be fused to the transmembrane protein RRBP1, presumably tethering RAF1 to the endoplasmatic reticulum with its kinase domain facing the cytoplasmic space. The oncogene RASGRP1 was predicted to be fused to ATP1A1, a protein embedded in the plasma membrane. Although oncogenes are more often found to constitute the C terminus of a fusion protein, RASGRP1 appeared to be fused to the N terminus of ATP1A1, thereby replacing several C-terminal domains of RASGRP1, which normally regulate recruitment to the plasma membrane, where RASGRP1 activates its target, KRAS (Beaulieu et al. 2007). Presumably, replacement of these regulatory domains by a membrane-bound protein increased the activity of RASGRP1 by means of warranting proximity to KRAS. (*C*) MCF10A and H6c7 cells were stably transduced with one of the fusion constructs or empty vector. MCF10A cells were cultured for 8 d without EGF, H6c7 cells were cultured for 7 d with EGF, and the area covered by cells was measured. Statistical significance was tested using a two-sided Welch *t*-test (MCF10A *RASGRP1-ATP1A1*: *P*-value = 0.023; MCF10A *RRBP1-RAF1*: *P*-value = 0.0094; H6c7 *RASGRP1-ATP1A1*: *P*-value = 4.1×10**^−^**^5^; H6c7 *RRBP1-RAF1*: *P*-value = 0.14). (*D*) Western blot showing increased phosphorylation of MAP2K1/2 (MEK1/2) and MAPK1/3 (ERK2/1) in *TP53*-deficient MCF10A cells stably transduced with one of the fusions as compared to empty vector.

Finally, we sought to experimentally validate predicted gene fusions as oncogenic drivers experimentally. We selected *RASGRP1-ATP1A1* and *RRBP1-RAF1* (Fig. 4A,B), because *RASGRP1* has not been implicated in oncogenic fusions before, and *RRBP1* is a novel partner of *RAF1* and was fused to near-full-length *RAF1* instead of exon 8, as is more common (Gao et al. 2018).

The fusions were introduced by lentiviral transduction into H6c7 cells, an immortalized human pancreatic duct epithelial cell line, and into *TP53*-deficient MCF10A cells, an EGF-dependent, immortalized human mammary epithelial cell line frequently used to determine the transforming potential of oncogenes (Stolze et al. 2015; Ng et al. 2018). Both fusions significantly enhanced EGF-independent colony formation relative to empty vector control (Fig. 4C; Supplemental Figs. S8, S9). Furthermore, the fusion proteins increased the phosphorylation of MAP2K1/2 (MEK1/2) and MAPK1/3 (ERK2/1) upon EGF withdrawal, indicating constitutive activation of the MAPK pathway (Fig. 4D). Together, these experiments confirmed the oncogenic activity of *RASGRP1-ATP1A1* and *RRBP1-RAF1* and further supported the notion that Arriba predicts oncogenic fusions with high confidence.

To test if the fusions could be addressed therapeutically, we treated cells with two compounds targeting the MAPK signaling axis: the RAF1 inhibitor sorafenib and the MAPK (ERK) inhibitor FR180204. Although the cell cultures responded to all compounds, fusion-positive cells did not prove to be more sensitive than empty vector controls (Supplemental Fig. S10).

## Discussion

We introduce Arriba, a novel computational tool for the detection of gene fusions from RNA-seq data, which delivers results in markedly shorter time than commonly used tools. This improvement in computational efficiency is even more pronounced when considering that Arriba's workflow is the only one to yield reusable alignments. All other presented methods align reads only for the sake of fusion detection, in a format that is not suitable for further processing. Although the workflow of STAR-Fusion is similar to that of Arriba, it requires the alignment parameters of STAR to be modified in a way that impairs downstream processing. As explained in the Methods section, Arriba avoids this requirement by using an extra extraction step.

At the same time, the benchmarks show that our approach does not sacrifice accuracy. In fact, Arriba shows extraordinary sensitivity and identifies fusions with subtle evidence in the RNA-seq data at higher precision than other methods. In addition, Arriba can detect some types of aberrant transcripts, which have so far been neglected in the development of most fusion detection algorithms. Intragenic rearrangements and translocations to intronic or intergenic regions may lead to the loss of function of the affected genes and thus represent important pieces of evidence in the characterization of dysfunctional tumor suppressor genes.

From a practical standpoint, it is also worth mentioning that only Arriba, InFusion, and STAR-Fusion processed all samples discussed in this work without issues. The other tools failed to process some samples, because the tools were either incompatible with certain data types, did not finish after several weeks, or reproducibly terminated with an error, thus requiring debugging and manual fixing for the problematic samples to be processed successfully (see Methods section).

### Shortcomings and future development

The STAR aligner does not report chimeric alignments that map to multiple loci. This complicates the detection of fusions involving genes with paralogs. For example, *CIC-DUX4* fusions in small round-cell sarcomas (Kawamura-Saito et al. 2006) are easily missed by Arriba owing to the presence of multiple copies of the *DUX4* gene in the human genome. For the same reason, the detection of integrated viral DNA into the host genome is impaired. A common and straightforward approach to detect viral integration is to align reads to concatenated genomes of the host and a collection of viruses. Viral integration can then be identified as reads aligning partially to both the host genome and a viral genome. Because related strains of viruses share a substantial fraction of sequence identity, this approach has a blind spot in regions conserved across strains. With version 2.6.0a, STAR introduced the ability to align chimeric reads to multiple loci in the genome, but such alignments are currently only reported in STAR's proprietary data format (the file *Chimeric.out.junction*). Once STAR reports multimapping chimeric alignments in a SAM-compliant format, Arriba can be enhanced to detect fusions that are supported by multimapping reads.

### Relevance of gene fusions in pancreatic cancer

We combined published data from a wide range of studies, yielding to our knowledge the largest collection of RNA-seq samples from pancreatic tumors to date. By applying Arriba to this collection, we discovered gene fusions in a notable fraction of *KRAS* wild-type tumors (25%) as well as four *KRAS* mutant cases. The fusions involved a variety of genes that have been shown to contribute to MAPK signaling, thus likely phenocopying the effect of activating *KRAS* point mutations that are present in pancreatic adenocarcinoma in >90% of cases (The Cancer Genome Atlas Research Network 2017).

Importantly, some of the lesions represent bona fide entry points for targeted therapeutic approaches, which have been applied with success in other cancer types. Non-small-cell lung carcinomas with *ALK* or *ROS1* fusions are sensitive to treatment with crizotinib and other, second- and third-generation inhibitors (Shaw et al. 2014). *NTRK*-rearranged pancreatic tumors are eligible for targeted inhibition with larotrectinib in accordance with the recent approval by the FDA for any solid tumor bearing *NTRK* fusions regardless of the origin (Drilon et al. 2018). We found three cases carrying fusions with *FGFR2*, which might predict response to ponatinib as previously shown in cholangiocellular carcinoma (Borad et al. 2015). BLU-667 is a highly specific RET inhibitor developed for the treatment of tumors with *RET* mutations and rearrangements, including *NCOA4-RET* and *CCDC6-RET* fusions, as observed in two of the analyzed pancreatic tumors. This drug is currently undergoing a phase 1 clinical trial (Subbiah et al. 2018). Furthermore, gene fusions affecting *BRAF* or *RAF1* are increasingly recognized as potential therapeutic targets for either direct (Ross et al. 2016) or indirect inhibition using MAPK (ERK) inhibitors (McEvoy et al. 2019), although we could not confirm the efficacy of such treatment regimens in our cell culture experiments. Together, of the 30 fusions identified by Arriba, 25 involved a fusion partner that is amenable to targeted therapy.

In view of the therapeutic relevance of these fusions and the overall high incidence of oncogenic fusions in *KRAS* wild-type pancreatic tumors, we recommend systematic testing of the *KRAS* mutation status and screening for gene fusions in the absence of *KRAS* mutations.

## Methods

### Arriba workflow

**Figure 5.**
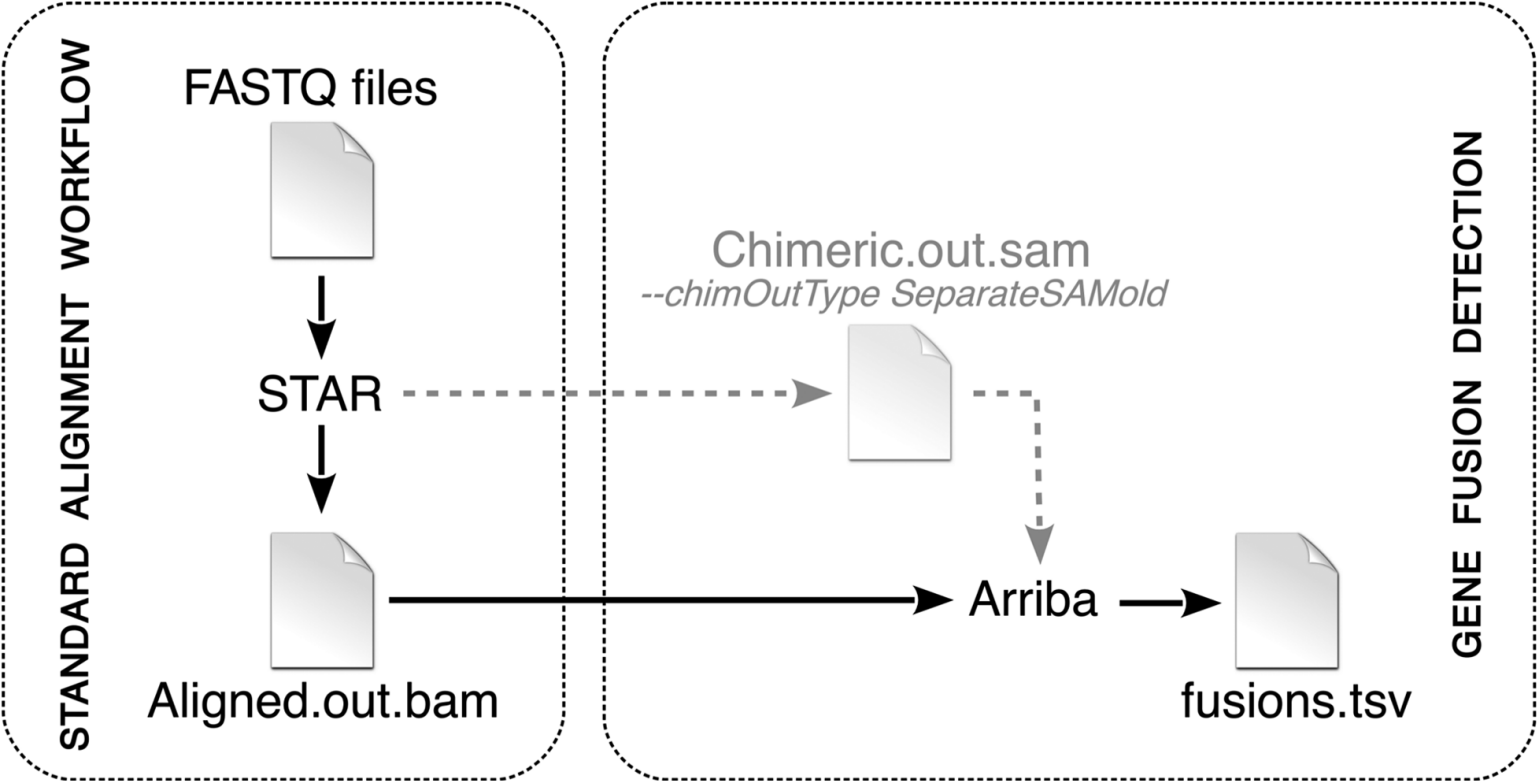
Arriba workflow. Arriba is an extension of a standard alignment workflow based on STAR. In legacy mode, STAR writes chimeric alignments to the file *Chimeric.out.sam*. In newer versions, STAR writes them to the main output file *Aligned.out.bam*. Arriba can take either file as input to search for gene fusions.

Many fusion detection algorithms attempt to boost sensitivity with the help of elaborate alignment methods. Common strategies use multiple rounds of alignment with iteratively trimmed reads (Jia et al. 2013), alignment with multiple algorithms (Nicorici et al. 2014), or alignment against assemblies generated on the fly (Davidson et al. 2015). Although these techniques improve the discovery of fusion-supporting reads, they come at the expense of long runtimes. In contrast, Arriba's workflow is linear with just a single alignment step followed by a filtering step (Fig. 5).

#### Extraction of chimeric reads

Arriba builds on the ultrafast STAR RNA-seq aligner (Dobin et al. 2013). When run with the parameter *‐‐chimSegmentMin*, STAR searches for two types of chimeric alignments: split reads, that is, reads with two segments aligning in a noncontiguous fashion, and discordant mates (also referred to as spanning reads or bridge reads), which are paired-end reads originating from the same fragment but with the mates aligning in a nonlinear way. The chimeric alignments are collected in a separate output file named *Chimeric.out.sam* or since STAR version 2.5.3a—when the parameter *‐‐chimOutType WithinBAM* is specified—in the main output file *Aligned.out.bam*. Arriba extracts the chimeric alignments from either of these files and integrates them to identify gene fusions.

STAR only reports an alignment as chimeric if a noncontiguous segment does not align to a downstream exon within a reasonable distance, as defined by the parameter *‐‐alignIntronMax*. Otherwise, it assumes that the gap in the alignment represents an intron skip and creates a gapped alignment. Some well-known oncogenic fusions arise from focal deletions, which pull the 5′ end of an upstream gene and the 3′ end of a downstream gene together. Prominent examples are fusions between *GOPC* and *ROS1* in lung adenocarcinoma (Suehara et al. 2012) or between *EIF3E* and *RSPO2* in colon cancer (Seshagiri et al. 2012). Instead of creating chimeric alignments, STAR aligns reads supporting these fusions as if the fused genes were joined by splicing, because STAR determines the type of alignment solely based on the size of the gap rather than the gene annotation. In addition to extracting chimeric alignments, Arriba also screens for alignments spanning the boundaries of annotated genes in order to avoid missing fusions resulting from focal deletions.

Unlike many other fusion detection pipelines, Arriba can reuse existing alignments of STAR rather than requiring reads to be aligned exclusively for the sake of calling gene fusions. STAR-Fusion is also capable of reusing existing alignments but requires that the STAR parameter *‐‐alignIntronMax* be reduced or else it is ignorant of fusions arising from focal deletions. However, setting this parameter smaller than the common intron size impairs the alignment quality, because many intron-spanning mates will be flagged as improperly paired. Of all presented methods, Arriba is the only one that offers a seamless integration into a standard RNA-seq alignment workflow. Alignments are a prerequisite to various types of analyses, such as the quantification of gene expression or the identification of allele-specific expression. Both are routine tasks in clinical research and necessitate the generation of alignments anyway. The ability to plug in Arriba as an extension to an existing RNA-seq workflow therefore makes fusion detection highly efficient, because it incurs negligible CPU time.

#### Filtering of artifacts

Once all candidate alignments have been collected, Arriba applies a set of filters to remove artifacts and to enrich for high-confidence predictions. There are negatively and positively selecting filters. Negatively selecting filters discard candidates deemed to be artifacts, such as candidates supported by reads with homopolymers, tandem repeats, or an excessive amount of mismatches; candidates between homologous genes; alignments with short anchors; and candidates with few supporting reads relative to the total number of candidates in the fusion partners. Moreover, a position-specific blacklist is applied to remove recurrent artifacts and transcripts observed in benign tissue. The blacklist was trained on RNA-seq samples from the Human Protein Atlas (Uhlen et al. 2015), the Illumina Human BodyMap2 (NCBI Sequence Read Archive [SRA; https://www.ncbi.nlm.nih.gov/sra] accession ERP000546), the ENCODE Project (The ENCODE Project Consortium 2012), the Roadmap Epigenomics Project (Roadmap Epigenomics Consortium et al. 2015), and the NCT/DKTK MASTER cohort (Horak et al. 2017). Positively selecting filters rescue candidates discarded by negatively selecting filters, provided that there is strong evidence that a candidate was discarded erroneously, such as candidates with breakpoints at annotated splice sites, a user-defined whitelist of known/highly recurrent fusions, or a correlating structural variant detected via WGS.

**Figure 6.**
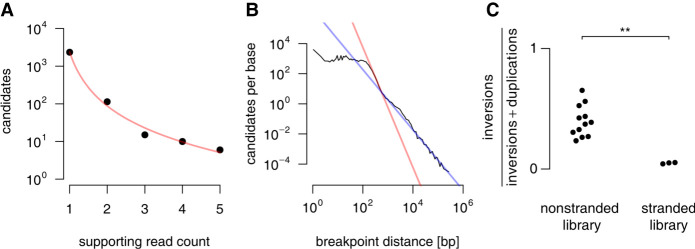
Covariates used to estimate the level of background noise. One of Arriba's artifact filters removes candidates with fewer supporting reads than the estimated level of background noise. For this purpose, Arriba calculates several covariates that correlate with the level of background noise. (*A*) Arriba assumes a polynomial relationship between the noise level (unfiltered candidates) and their number of supporting reads. The data shown here are based on the highly expressed housekeeping gene *GAPDH* in the MCF-7 cell line (SRA accession ERR358487). (*B*) The figure shows the number of unfiltered candidates as a function of the breakpoint distance averaged over all genes in the MCF-7 cell line. Artifacts tend to have breakpoints in close proximity as evidenced by a sharp increase in the number of candidates with decreasing distance. Arriba fits two models depending on whether the breakpoints are closer or further apart than 400 bp (red and blue lines, respectively). (*C*) The library preparation method can affect the proportions of artifacts. For example, the samples from Heining et al. (2018) are a mixture of stranded and nonstranded libraries. The stranded libraries are enriched for duplications compared with the nonstranded libraries (two-sided Wilcoxon rank-sum test, *P*-value = 0.0044).

Positively selecting filters and the statistical model used to filter candidates by their number of supporting reads are the key features which accomplish Arriba's high sensitivity. Arriba assumes a polynomial relationship between the number of supporting reads and the level of background noise. Only candidates with more supporting reads than the estimated level of background noise are reported (Fig. 6A). In addition, the model incorporates several covariates that correlate with the level of background noise, including the sequencing depth, the breakpoint distance (Fig. 6B), the library preparation protocol (stranded vs. nonstranded) (Fig. 6C), and the location of the breakpoints (intron vs. exon vs. splice site). Based on the number of reads supporting a candidate, the expected level of background noise (e-value) is calculated using Equation 1. In the following, lowercase components of the equations represent dynamically calculated variables; uppercase components are empirically determined constants, which were trained on RNA-seq samples from the NCT/DKTK MASTER cohort and proved to be reasonably stable across different data sets.

$$\begin{array}{r} e-\mathrm{value}=\mathrm{base\_level\_bg\_noise}*\mathrm{depth\_penalty}\\ {}*\;\mathrm{distance\_penalty}\;*\;\mathrm{inv\_to\_dup\_ratio}\;*\;\mathrm{intron\_to\_exon\_ratio} \end{array}$$

The base level of background noise is computed for each gene individually. It increases linearly with the total number of candidates in a gene and decreases in a polynomial manner as a function of the number of supporting reads:

$$\begin{array}{rl} & \mathrm{base\_level\_bg\_noise}=\frac{\mathrm{total\_candidates\_of\_gene}}{\mathrm{sum\_of\_exon\_lengths\_of\_gene}}\\ & *\;{\left(\mathrm{supporting\_reads}-\mathrm{SHIF}T_{\mathrm{noise}}\right)}^{\mathrm{SLOP}E_{\mathrm{noise}}}\;*\;\mathrm{INTERCEP}T_{\mathrm{noise}}\\ & \mathrm{with}\;\;\;\mathrm{SHIF}T_{\mathrm{noise}}=-0.73\;\;\;\mathrm{and}\;\;\;\mathrm{SLOP}E_{\mathrm{noise}}=-2.28\;\;\;\mathrm{and}\;\\ & \mathrm{INTERCEP}T_{\mathrm{noise}}=10^{-1.75}\; \end{array}$$

The depth penalty increases linearly with the total number of mapped reads. The slope of the linear function decreases with increasing number of supporting reads:

$$\begin{array}{rl} & \mathrm{depth\_penalty}=\mathrm{SLOP}E_{\mathrm{depth}}\;*\;{\left(\mathrm{SLOPE\_MODIFIER}\right)}^{\mathrm{supporting\_reads}}\\ & \;*\;\mathrm{mapped\_reads}\\ & \mathrm{with}\;\;\;\mathrm{SLOP}E_{\mathrm{depth}}=2\;*\;10^{-11}\;\;\;\;\mathrm{and}\;\;\mathrm{SLOPE\_MODIFIER}=0.02\; \end{array}$$

The distance penalty is applied to breakpoints <400 kb apart. It increases polynomially with decreasing distance of the breakpoints. Two different model fits are used depending on whether the breakpoints are closer or further apart than 400 bp:

$$\begin{array}{rl} & \mathrm{distance\_penalty}=({\mathrm{distance})}^{\mathrm{SLOP}E_{\mathrm{distance}}}\;*\;\mathrm{INTERCEP}T_{\mathrm{distance}}\\ & \mathrm{with}\;\;\{\begin{array}{c} \mathrm{SLOP}E_{\mathrm{distance}}=-4.58\;\;\;\;\mathrm{and}\;\;\mathrm{INTERCEP}T_{\mathrm{distance}}=8.27\;*\;10^{10},\mathrm{if}\;\;\;\;\mathrm{distance}<400\;\;\mathrm{bp}\\ \mathrm{SLOP}E_{\mathrm{distance}}=-1.53\;\;\;\;\mathrm{and}\;\;\mathrm{INTERCEP}T_{\mathrm{distance}}=3.73\;*\;10^{8},\;\;\;\;\;\mathrm{if}\;\;\;\;\mathrm{distance}\geq 400\;\;\mathrm{bp} \end{array} \end{array}$$

Arriba calculates the ratio of inversions to duplications, which is influenced by the library preparation protocol. For example, some stranded libraries are prone to induce artifacts resembling duplications. Duplications and inversions are therefore penalized in proportion to their relative frequency:

$$\begin{array}{rl} & \mathrm{inv\_to\_dup\_ratio}=\\ & \frac{1}{\mathrm{total\_candidates}}\;*\;\{\begin{array}{c} \mathrm{total\_inversions},\;\;\;\;\mathrm{if}\;\;\;\;\mathrm{event}\;\;\;\;\mathrm{type}\;\;\;\;\mathrm{is}\;\;\;\;\mathrm{inversion}\\ \mathrm{total\_duplications},\;\;\;\;\mathrm{if}\;\;\;\;\mathrm{event}\;\;\;\;\mathrm{type}\;\;\;\;\mathrm{is}\;\;\;\;\mathrm{duplication} \end{array} \end{array}$$

Likewise, candidates are penalized based on where the breakpoints are located and the relative frequencies of candidates with breakpoints in introns, in exons, or at splice sites:

$$\begin{array}{rl} & \mathrm{intron\_to\_exon\_ratio}=\\ & \frac{1}{\mathrm{total\_candidates}}\;*\;\{\begin{array}{c} \mathrm{total\_intronic\_candidates},\;\;\;\;\mathrm{if}\;\;\;\;\mathrm{breakpoint}\;\;\;\;\mathrm{is}\;\;\;\;\mathrm{intronic}\\ \mathrm{total\_exonic\_candidates},\;\;\;\;\mathrm{if}\;\;\;\;\mathrm{breakpoint}\;\;\;\;\mathrm{is}\;\;\;\;\mathrm{exonic}\\ \mathrm{total\_spliced\_candidates},\;\;\;\;\mathrm{if}\;\;\;\;\mathrm{breakpoint}\;\;\;\;\mathrm{is}\;\;\;\;\mathrm{spliced} \end{array} \end{array}$$

Arriba's sensitivity is boosted further by two positively selecting filters, which recover candidates discarded owing to an insufficient number of supporting reads: One filter selects candidates having both breakpoints at splice sites; another filter selects fusions between genes linked by at least four distinct fusion transcripts as evidenced by four or more breakpoints coinciding with a splice site in one of the genes (but not necessarily in both).

### Benchmarking

All fusion detection tools were run with default parameters with the following exceptions: The parameter *-junL* of PRADA has no default value and was set to 80% of the read length as recommended by the developers. For benchmarks regarding the detection of fusions with intergenic breakpoints, InFusion was executed with the parameters *‐‐allow-intronic*, *‐‐allow-intergenic*, and *‐‐allow-non-coding*. Otherwise, InFusion does not call this type of events. By default, FusionCatcher uses an internal list of known oncogenic fusions to improve sensitivity. For an unbiased benchmark that is more reflective of FusionCatcher's sensitivity for de novo fusion discovery, we disabled this list by calling FusionCatcher with the parameter *‐‐skip-known-fusions*. In addition, the default value of the parameter *‐‐allowed-labels* of the script *extract_fusion_genes.py* had to be emptied for the parameter *‐‐skip-known-fusions* to take effect.

Wall clock time, CPU time, and memory consumption were measured by the GNU *time* utility.

We considered a prediction to be a true positive if the fusion partners matched a list of validated fusions or if the breakpoints were within a distance of 100 kb from a structural variant detected in a matched WGS sample. The orientation of the genomic breakpoints was not required to match the orientation of the transcriptomic breakpoints, because FusionCatcher does not report this information. Whether orientation was considered or not had marginal effect on the results, however. The predicted breakpoints of all tools were reannotated with the GENCODE v19 gene model to harmonize the gene names. If a tool reported multiple alternatively spliced transcript variants involving the same pair of genes and thus arising from the same genomic rearrangement, only one of the transcripts was counted. Similarly, if a pair of breakpoints overlapped with multiple genes and was reported more than once with different gene names, only one of the instances was counted. PRADA and SOAPfuse do not sort their output by confidence. The predictions of these tools were therefore ranked by the number of supporting reads in decreasing order. The predictions of deFuse were sorted by the column *probability*.

### Validation of fusion predictions from the MCF-7 cell line

For each fusion detection method, we subjected the top predictions from the MCF-7 cell line to experimental validation using Sanger sequencing if the prediction had not been validated in a previous study (Davidson et al. 2015) or confirmed by a structural variant (Li et al. 2016). We selected fusion predictions that were made in at least two independent batches of the MCF-7 cell line to avoid selecting batch-specific fusions (Supplemental Table S3). The fusion-specific primers were designed using Primer3 (Untergasser et al. 2012). One microgram of MCF-7 RNA was transcribed into cDNA using SuperScript III (Invitrogen) reverse transcriptase according to the manufacturer's instructions and used as template for polymerase chain reactions (PCRs). PCRs were performed with Taq PCR master mix (2×; Roboklon) according to the manufacturer's instructions and the following PCR conditions: initial denaturation of 5 min; 35 cycles or denaturation of 1 min at 95°C, annealing of 1 min at 60°C, and elongation of 1 min at 72°C; and final elongation of 2 min. The PCR products were separated electrophoretically in 2% agarose gels and visualized. Bands at the expected height were cut out and purified for sequencing. The sequencing was performed on a 3500 capillary sequencer (Applied Biosystems) according to the manufacturer's instructions.

### Sample collection

The sample collection procedures and ethics approvals can be found in the respective publications of the samples that were analyzed in this study (Barretina et al. 2012; Carugo et al. 2016; Diaferia et al. 2016; Kirby et al. 2016; Witkiewicz et al. 2016; Bhattacharyya et al. 2017; Horak et al. 2017; Nicolle et al. 2017; The Cancer Genome Atlas Research Network 2017; Aung et al. 2018; Heining et al. 2018; Lomberk et al. 2018; Bryant et al. 2019; Lin et al. 2019; Maurer et al. 2019). In addition to the published samples, we included samples from one *KRAS* wild-type pancreatic cancer patient that was recruited in the NCT/DKTK MASTER cohort and had not been published yet. The samples from this patient were collected and prepared as previously described (Heining et al. 2018). The patient gave written informed consent in accordance with protocol S-206/2011 approved by the Ethics Committee of the University of Heidelberg. Permission to publish the results presented in this study is covered by the written informed consent given by the patients.

The raw sequencing data supporting the findings of this study were obtained from the NCBI Sequence Read Archive (SRA; https://www.ncbi.nlm.nih.gov/sra) using the accession numbers ERP107752, SRP102440, SRP051606, SRP072492, SRP072493, ERP022034, ERP023824, ERP015474, SRP077921, SRP161484, SRP096338, and SRP158639; from the Genomics Data Commons Portal (GDC; https://portal.gdc.cancer.gov/) using the accession numbers CCLE-PAAD and TCGA-PAAD; and from the European Genome-Phenome Archive (EGA; https://ega-archive.org/) using the accession numbers EGAD00001003584, EGAD00001003582, EGAD00001003410, EGAD00001003945, EGAD00001003972, EGAD00001004068, and EGAD00001005069.

### Identification of fusions from pancreatic cancer samples

We ran STAR version 2.5.3a with the following parameters to align RNA-seq reads: *‐‐outFilterMultimapNmax 1 *‐‐*outFilterMismatchNmax 3 *‐‐*outFilterMismatchNoverLmax 0*.3 ‐‐*alignIntronMax 500000 *‐‐*alignMatesGapMax 500000 ‐‐chimSegmentMin 10 ‐‐chimJunctionOverhangMin 10 ‐‐chimScoreMin 1 ‐‐chimScoreDropMax 30 ‐‐chimScoreJunctionNonGTAG 0 ‐‐chimScoreSeparation 1 ‐‐alignSJstitchMismatchNmax 5 -1 5 5 ‐‐chimSegmentReadGapMax 3 *‐‐*chimMainSegmentMultNmax 10*. The STAR index was created using the GENCODE gene model (v19 for human, vM12 for mouse) and the parameter *‐‐sjdbOverhang 200*. Reads were aligned against concatenated assemblies of the 1000 Genomes Phase II human reference genome (hs37d5) and the PhiX genome (*NC_001422.1*). Samples from patient-derived xenograft mouse models were aligned against concatenated assemblies of the human (hs37d5+PhiX) and murine (mm10) reference genomes.

Gene fusion tools were run with the same parameters as for the benchmark. For some tools, manual intervention was required to make the pipelines complete successfully on a small subset of the samples: FusionCatcher sometimes failed to parse the read identifiers, which could be solved by reformatting the identifiers of the offending samples. The java virtual machine launched by PRADA's script *fromfq.pbs* occasionally ran out of memory and had to be increased to 64 GB (from 8 GB) using the parameters *-Xmx64g* and *-XX:+UseSerialGC*. Moreover, PRADA uses the IS linear-time algorithm for construction of BWA indices by default. This algorithm is not suitable for indices >2 GB in size and thus had to be changed to the BWT-SW algorithm by calling BWA with the parameter *-a bwtsw* in the script *prada-fusion* for larger samples. Occasionally, deFuse terminated with an error, because the executable *calccov* reported the text *nan* instead of numeric values for a small set of genome coordinates. The pipeline completed when the illegal values were replaced by zeros. SOAPfuse and deFuse ran for >4 wk on some samples and were terminated prematurely.

When a matched WGS sample was available, we expected a fusion prediction to be confirmed by a nearby structural variant. Only structural variants within a distance of 100 kbp and matching orientation were recognized as correlating events. DNA-seq samples from Heining et al. (2018) and from this study were aligned as previously described (Heining et al. 2018); all other DNA-seq samples were aligned using the PanCancer BWA-MEM alignment workflow (https://github.com/ICGC-TCGA-PanCancer/Seqware-BWA-Workflow). We used our previously reported pipeline SOPHIA version 35 (https://bitbucket.org/utoprak/sophia/src) to call structural variants (Heining et al. 2018).

We used the *mpileup*, *call*, and *filter* modules of BCFtools (Li 2011) version 1.6 in conjunction with Annovar (Wang et al. 2010) version 2016-02-01 to identify *KRAS* mutations. BCFtools was configured to report all reference mismatches supported by at least two reads and ≥10% allele fraction. In addition, mutations at codons other than 11, 12, 13, and 61 were manually curated by inspecting the supporting reads in IGV. When no *KRAS* missense mutation was found in the RNA-seq data, the mutation status of *KRAS* was taken from the respective study, whenever available (Barretina et al. 2012; Witkiewicz et al. 2016; Horak et al. 2017; Nicolle et al. 2017; The Cancer Genome Atlas Research Network 2017; Aung et al. 2018).

To identify replicates within and across the collected cohorts, we compared the genotype of all samples at 1000 common SNP positions. Samples that grouped together using Euclidean distance-based hierarchical clustering were considered to be replicates and were either merged or kept from only one cohort.

We inferred from a combination of features whether a gene fusion should be considered a (putative) driver, including the expression level, Arriba's confidence score, preservation of the reading frame, retention of essential domains for oncogenic activity, and whether the genes had previously been described to be involved in oncogenic fusions in pancreatic cancer or other entities. Pfam protein domains were mapped from protein coordinates to genomic coordinates using the R/Bioconductor package PBase. Genomic coordinates of transmembrane domains were obtained from UniProt (The UniProt Consortium 2018). The most promising fusion candidates were visually inspected in IGV to identify potential alignment artifacts. Patient PCSI_0326 from the PACA-CA cohort carried a *TRIM24-BRAF* fusion. Arriba only reported a fusion transcript with a predicted frame shift. Closer inspection of soft-clipped reads in *BRAF* suggested that some reads linked exon nine of *TRIM24* to exon eight of *BRAF*, as revealed by the built-in BLAT utility of IGV. Presumably, STAR failed to align these reads because they included 20 bases from intron seven of *BRAF* (*Chr 7: 140,498,293–140,498,312*), which cannot be mapped uniquely to the human genome. These bases correct the reading frame to yield an in-frame fusion transcript.

The analysis of overrepresented genes by pathway was performed with the help of WebGestalt (Wang et al. 2017). We used all human protein-coding genes as background and pathways of the KEGG database (Kanehisa et al. 2017) as gene sets to be tested for overrepresentation.

### Lentiviral transduction

The MCF10A cell line was obtained from the American Type Culture Collection and cultured with DMEM medium supplemented with 5% horse serum, 0.5 mg/mL hydrocortisone, 100 ng/mL cholera toxin, 10 µg/mL insulin, and 20 ng/mL EGF. *TP53* knockout was performed by CRISPR-Cas9-mediated gene editing, and nine clones with confirmed homozygous knockout were pooled to obtain the *TP53*-deficient MCF10A cell line. The H6c7 cell line was obtained from Kerafast and cultured with Keratinocyte serum-free medium supplemented with 50 ng/mL bovine pituitary extract and 5 ng/mL EGF.

The fusion genes were synthesized by Trenzyme GmbH and cloned into the lentiviral expression vector pLenti6.2/V5-DEST (Invitrogen). Production of lentiviral particles and transduction of MCF10A and H6c7 cells was performed as previously described (Stolze et al. 2015). Transduced cells were selected with 10 µg/mL blasticidin to obtain cell lines with stable expression of the fusion genes or empty vector control.

### Quantitative RT-PCR

Total RNA was isolated using the RNeasy mini kit (Qiagen) and reverse-transcribed using TaqMan reverse transcription reagents (Applied Biosystems), and the expression of the fusion transcripts was measured by quantitative RT-PCR (Supplemental Fig. S11) using the following primers: *RRBP1-RAF1* forward (5′-CACCGGGACATGAAGTCCAA-3′), *RRBP1-RAF1* reverse (5′-GATCCTGTAGGCTGCTCGAC-3′), *RASGRP1-ATP1A1* forward (5′-CTATCTGGAACTCGGCGGAC-3′), *RASGRP1-ATP1A1* reverse (5′-ACGAAGCACAGGTTGTCGAT-3′). Fusion gene expression was calculated relative to endogenous peptidylprolyl isomerase B (*PPIB*) using the primers *PPIB* forward (5′-GAGGAAAGAGCATCTACGGTG-3′) and *PPIB* reverse (5′-GCTTCTCCACCTCGATCTTG-3′).

### Colony formation assays

For measurement of colony formation, 500 MCF10A cells were seeded in six-well plates in growth medium without EGF and cultured for 8 d, and 5000 H6c7 cells were cultured for 7 d in growth medium with EGF. Cells were subsequently fixed with 100% methanol and stained with 2.5% crystal violet solution. Quantification was performed using ImageJ/Fiji by determining the area covered by cells (Guzmán et al. 2014).

### Western blotting

Cell pellets were lysed with RIPA buffer (50 mM Tris-HCl, 150 mM NaCl, 0.1% SDS, 0.5% sodium deoxycholate, 1% Triton X-100, Halt protease and phosphatase inhibitor cocktail [1:100]). Protein extracts (50 μg) were subjected to SDS-PAGE and transferred to nitrocellulose membranes using the trans-blot turbo transfer system (Bio-Rad). Membranes were blocked with 5% dry milk in TBST, followed by incubation with primary and fluorescence-labeled secondary antibodies. Fluorescence signals were imaged using the Odyssey CLx western blot detection system (LI-COR). The following antibodies were used: anti-phospho-MEK1/2 (Cell Signaling Technology 9121), anti-MEK1/2 (Cell Signaling Technology 4694), anti-phospho-p44/42 MAPK (ERK1/2; Thr202/Tyr204; Cell Signaling Technology 4376), anti-p44/42 MAPK (ERK1/2; Cell Signaling Technology 4696), anti-HSP90 (Santa Cruz sc-7947), goat anti-rabbit IgG DyLight 680 Conjugate (Cell Signaling Technology 5366S), and anti-mouse IgG DyLight 800 4x PEG Conjugate (Cell Signaling Technology 5257S).

### Drug sensitivity

For dose-response curves, 2000 MCF10A cells were seeded in 96-well plates and treated with the indicated concentrations of the MAPK (ERK) inhibitor FR180204 (Hölzel Diagnostika) or the RAF1 inhibitor sorafenib (TargetMol) in EGF-depleted medium, and viability was assessed by CellTiter 96 Aqueous One Solution Cell Proliferation Assay (Promega) MTS assay after 48 h.

### Software availability

Arriba was written in C++ and R (R Core Team 2017). The most recent source code and precompiled binaries are available for the Linux operating system under the MIT and GPLv3 licenses at GitHub (https://github.com/suhrig/arriba). The Arriba version used in this work (1.0.0) is also available in Supplemental Code S1.

## Data access

All raw and processed sequencing data generated in this study have been submitted to the European Genome-phenome Archive (EGA; https://ega-archive.org/) under Study ID EGAS00001003554 (Dataset ID: EGAD00001005069).

## Competing interest statement

S.F. reports consulting or advisory board membership for Bayer and Roche and has received honoraria from Amgen, Eli Lilly, PharmaMar, and Roche as well as research funding from AstraZeneca, Pfizer, and PharmaMar and travel or accommodation expenses from Amgen, Eli Lilly, PharmaMar, and Roche. A.S. is a member of the advisory board/speaker's bureau of Astra Zeneca, AGCT, Bayer, BMS, Eli Lilly, Illumina, Janssen, MSD, Novartis, Pfizer, Roche, Seattle Genetics, Takeda, and Thermo Fisher Scientific and has received grants from Bayer, BMS, and Chugai. No potential conflicts of interest were disclosed by the other authors.

## Supplementary Material

Supplemental Material

## References

[GR257246UHRC1] An X, Tiwari AK, Sun Y, Ding PR, Ashby CR Jr, Chen ZS. 2010. BCR-ABL tyrosine kinase inhibitors in the treatment of Philadelphia chromosome positive chronic myeloid leukemia: a review. Leuk Res 34: 1255–1268. 10.1016/j.leukres.2010.04.016 20537386

[GR257246UHRC2] Aung KL, Fischer SE, Denroche RE, Jang GH, Dodd A, Creighton S, Southwood B, Liang SB, Chadwick D, Zhang A, 2018. Genomics-driven precision medicine for advanced pancreatic cancer: early results from the COMPASS trial. Clin Cancer Res 24: 1344–1354. 10.1158/1078-0432.CCR-17-2994 29288237 PMC5968824

[GR257246UHRC3] Barretina J, Caponigro G, Stransky N, Venkatesan K, Margolin AA, Kim S, Wilson CJ, Lehár J, Kryukov GV, Sonkin D, 2012. The cancer cell line encyclopedia enables predictive modelling of anticancer drug sensitivity. Nature 483: 603–607. 10.1038/nature11003 22460905 PMC3320027

[GR257246UHRC4] Beaulieu N, Zahedi B, Goulding RE, Tazmini G, Anthony KV, Omeis SL, de Jong DR, Kay RJ. 2007. Regulation of RasGRP1 by B cell antigen receptor requires cooperativity between three domains controlling translocation to the plasma membrane. Mol Biol Cell 18: 3156–3168. 10.1091/mbc.e06-10-0932 17567957 PMC1949348

[GR257246UHRC5] Bhattacharyya S, Pradhan K, Campbell N, Mazdo J, Vasantkumar A, Maqbool S, Bhagat TD, Gupta S, Suzuki M, Yu Y, 2017. Altered hydroxymethylation is seen at regulatory regions in pancreatic cancer and regulates oncogenic pathways. Genome Res 27: 1830–1842. 10.1101/gr.222794.117 28986391 PMC5668941

[GR257246UHRC6] Borad MJ, Gores GJ, Roberts LR. 2015. Fibroblast growth factor receptor 2 fusions as a target for treating cholangiocarcinoma. Curr Opin Gastroenterol 31: 264–268. 10.1097/MOG.0000000000000171 25763789 PMC4750878

[GR257246UHRC7] Bryant KL, Stalnecker CA, Zeitouni D, Klomp JE, Peng S, Tikunov AP, Gunda V, Pierobon M, Waters AM, George SD, 2019. Combination of ERK and autophagy inhibition as a treatment approach for pancreatic cancer. Nat Med 25: 628–640. 10.1038/s41591-019-0368-8 30833752 PMC6484853

[GR257246UHRC8] The Cancer Genome Atlas Research Network. 2017. Integrated genomic characterization of pancreatic ductal adenocarcinoma. Cancer cell 32: 185–203.e13. 10.1016/j.ccell.2017.07.007 28810144 PMC5964983

[GR257246UHRC9] Carugo A, Genovese G, Seth S, Nezi L, Rose JL, Bossi D, Cicalese A, Shah PK, Viale A, Pettazzoni PF, 2016. In vivo functional platform targeting patient-derived xenografts identifies WDR5-Myc association as a critical determinant of pancreatic cancer. Cell Rep 16: 133–147. 10.1016/j.celrep.2016.05.063 27320920

[GR257246UHRC10] Catic A, Kurtovic-Kozaric A, Johnson SH, Vasmatzis G, Pins MR, Kogan J. 2017. A novel cytogenetic and molecular characterization of renal metanephric adenoma: identification of partner genes involved in translocation t(9;15)(p24;q24). Cancer Genet 214-215: 9–15. 10.1016/j.cancergen.2017.03.001 28595733

[GR257246UHRC11] Conroy T, Hammel P, Hebbar M, Ben Abdelghani M, Wei AC, Raoul JL, Choné L, Francois E, Artru P, Biagi JJ, 2018. FOLFIRINOX or gemcitabine as adjuvant therapy for pancreatic cancer. N Engl J Med 379: 2395–2406. 10.1056/NEJMoa1809775 30575490

[GR257246UHRC12] Davidson NM, Majewski IJ, Oshlack A. 2015. JAFFA: high sensitivity transcriptome-focused fusion gene detection. Genome Med 7: 43. 10.1186/s13073-015-0167-x 26019724 PMC4445815

[GR257246UHRC13] Diaferia GR, Balestrieri C, Prosperini E, Nicoli P, Spaggiari P, Zerbi A, Natoli G. 2016. Dissection of transcriptional and *cis*-regulatory control of differentiation in human pancreatic cancer. EMBO J 35: 595–617. 10.15252/embj.201592404 26769127 PMC4801945

[GR257246UHRC14] Dobin A, Davis CA, Schlesinger F, Drenkow J, Zaleski C, Jha S, Batut P, Chaisson M, Gingeras TR. 2013. STAR: ultrafast universal RNA-seq aligner. Bioinformatics 29: 15–21. 10.1093/bioinformatics/bts635 23104886 PMC3530905

[GR257246UHRC15] Drilon A, Laetsch TW, Kummar S, DuBois SG, Lassen UN, Demetri GD, Nathenson M, Doebele RC, Farago AF, Pappo AS, 2018. Efficacy of larotrectinib in *TRK* fusion-positive cancers in adults and children. N Engl J Med 378: 731–739. 10.1056/NEJMoa1714448 29466156 PMC5857389

[GR257246UHRC16] Edgren H, Murumagi A, Kangaspeska S, Nicorici D, Hongisto V, Kleivi K, Rye IH, Nyberg S, Wolf M, Borresen-Dale AL, 2011. Identification of fusion genes in breast cancer by paired-end RNA-sequencing. Genome Biol 12: R6. 10.1186/gb-2011-12-1-r6 21247443 PMC3091304

[GR257246UHRC17] El-Gebali S, Mistry J, Bateman A, Eddy SR, Luciani A, Potter SC, Qureshi M, Richardson LJ, Salazar GA, Smart A, 2019. The Pfam protein families database in 2019. Nucleic Acids Res 47: D427–D432. 10.1093/nar/gky995 30357350 PMC6324024

[GR257246UHRC18] The ENCODE Project Consortium. 2012. An integrated encyclopedia of DNA elements in the human genome. Nature 489: 57–74. 10.1038/nature11247 22955616 PMC3439153

[GR257246UHRC19] Gao Q, Liang WW, Foltz SM, Mutharasu G, Jayasinghe RG, Cao S, Liao WW, Reynolds SM, Wyczalkowski MA, Yao L, 2018. Driver fusions and their implications in the development and treatment of human cancers. Cell Rep 23: 227–238.e3. 10.1016/j.celrep.2018.03.050 29617662 PMC5916809

[GR257246UHRC20] Gerhauser C, Favero F, Risch T, Simon R, Feuerbach L, Assenov Y, Heckmann D, Sidiropoulos N, Waszak SM, Hubschmann D, 2018. Molecular evolution of early-onset prostate cancer identifies molecular risk markers and clinical trajectories. Cancer Cell 34: 996–1011.e8. 10.1016/j.ccell.2018.10.016 30537516 PMC7444093

[GR257246UHRC21] Golan T, Hammel P, Reni M, Van Cutsem E, Macarulla T, Hall MJ, Park JO, Hochhauser D, Arnold D, Oh DY, 2019. Maintenance olaparib for germline *BRCA*-mutated metastatic pancreatic cancer. N Engl J Med 381: 317–327. 10.1056/NEJMoa1903387. 31157963 PMC6810605

[GR257246UHRC22] Guzmán C, Bagga M, Kaur A, Westermarck J, Abankwa D. 2014. Colonyarea: an imageJ plugin to automatically quantify colony formation in clonogenic assays. PLoS One 9: e92444. 10.1371/journal.pone.0092444 24647355 PMC3960247

[GR257246UHRC23] Haas BJ, Dobin A, Li B, Stransky N, Pochet N, Regev A. 2019. Accuracy assessment of fusion transcript detection via read-mapping and de novo fusion transcript assembly-based methods. Genome Biol 20: 213. 10.1186/s13059-019-1842-9 31639029 PMC6802306

[GR257246UHRC24] Heining C, Horak P, Uhrig S, Codo PL, Klink B, Hutter B, Fröhlich M, Bonekamp D, Richter D, Steiger K, 2018. *NRG1* fusions in *KRAS* wild-type pancreatic cancer. Cancer Discov 8: 1087–1095. 10.1158/2159-8290.CD-18-0036 29802158

[GR257246UHRC25] Honeyman JN, Simon EP, Robine N, Chiaroni-Clarke R, Darcy DG, Lim II, Gleason CE, Murphy JM, Rosenberg BR, Teegan L, 2014. Detection of a recurrent DNAJB1-PRKACA chimeric transcript in fibrolamellar hepatocellular carcinoma. Science 343: 1010–1014. 10.1126/science.1249484 24578576 PMC4286414

[GR257246UHRC26] Horak P, Klink B, Heining C, Gröschel S, Hutter B, Fröhlich M, Uhrig S, Hübschmann D, Schlesner M, Eils R, 2017. Precision oncology based on omics data: the NCT Heidelberg experience. Int J Cancer 141: 877–886. 10.1002/ijc.30828 28597939

[GR257246UHRC27] Jia W, Qiu K, He M, Song P, Zhou Q, Zhou F, Yu Y, Zhu D, Nickerson ML, Wan S, 2013. SOAPfuse: an algorithm for identifying fusion transcripts from paired-end RNA-Seq data. Genome Biol 14: R12. 10.1186/gb-2013-14-2-r12 23409703 PMC4054009

[GR257246UHRC28] Kanehisa M, Furumichi M, Tanabe M, Sato Y, Morishima K. 2017. KEGG: new perspectives on genomes, pathways, diseases and drugs. Nucleic Acids Res 45: D353–D361. 10.1093/nar/gkw1092 27899662 PMC5210567

[GR257246UHRC29] Kawamura-Saito M, Yamazaki Y, Kaneko K, Kawaguchi N, Kanda H, Mukai H, Gotoh T, Motoi T, Fukayama M, Aburatani H, 2006. Fusion between CIC and DUX4 up-regulates PEA3 family genes in Ewing-like sarcomas with t(4;19)(q35;q13) translocation. Hum Mol Genet 15: 2125–2137. 10.1093/hmg/ddl136 16717057

[GR257246UHRC30] Kirby MK, Ramaker RC, Gertz J, Davis NS, Johnston BE, Oliver PG, Sexton KC, Greeno EW, Christein JD, Heslin MJ, 2016. RNA sequencing of pancreatic adenocarcinoma tumors yields novel expression patterns associated with long-term survival and reveals a role for *ANGPTL4*. Mol Oncol 10: 1169–1182. 10.1016/j.molonc.2016.05.004 27282075 PMC5423196

[GR257246UHRC31] Li H. 2011. A statistical framework for SNP calling, mutation discovery, association mapping and population genetical parameter estimation from sequencing data. Bioinformatics 27: 2987–2993. 10.1093/bioinformatics/btr509 21903627 PMC3198575

[GR257246UHRC32] Li Y, Zhou S, Schwartz DC, Ma J. 2016. Allele-Specific quantification of structural variations in cancer genomes. Cell Syst 3: 21–34. 10.1016/j.cels.2016.05.007 27453446 PMC4965314

[GR257246UHRC33] Lier A, Penzel R, Heining C, Horak P, Fröhlich M, Uhrig S, Budczies J, Kirchner M, Volckmar A-L, Hutter B, 2018. Validating comprehensive next-generation sequencing results for precision oncology: the NCT/DKTK molecularly aided stratification for tumor eradication research experience. JCO Precision Oncology 10.1200/po.18.00171 35135162

[GR257246UHRC34] Lin IH, Chen DT, Chang YF, Lee YL, Su CH, Cheng C, Tsai YC, Ng SC, Chen HT, Lee MC, 2015. Hierarchical clustering of breast cancer methylomes revealed differentially methylated and expressed breast cancer genes. PLoS One 10: e0118453. 10.1371/journal.pone.0118453 25706888 PMC4338251

[GR257246UHRC35] Lin J, Wu YJ, Liang X, Ji M, Ying HM, Wang XY, Sun X, Shao CH, Zhan LX, Zhang Y. 2019. Network-based integration of mRNA and miRNA profiles reveals new target genes involved in pancreatic cancer. Mol Carcinog 58: 206–218. 10.1002/mc.22920 30294829

[GR257246UHRC36] Lomberk G, Blum Y, Nicolle R, Nair A, Gaonkar KS, Marisa L, Mathison A, Sun Z, Yan H, Elarouci N, 2018. Distinct epigenetic landscapes underlie the pathobiology of pancreatic cancer subtypes. Nat Commun 9: 1978. 10.1038/s41467-018-04383-6 29773832 PMC5958058

[GR257246UHRC37] Maurer C, Holmstrom SR, He J, Laise P, Su T, Ahmed A, Hibshoosh H, Chabot JA, Oberstein PE, Sepulveda AR, 2019. Experimental microdissection enables functional harmonisation of pancreatic cancer subtypes. Gut 68: 1034–1043. 10.1136/gutjnl-2018-317706 30658994 PMC6509007

[GR257246UHRC38] McEvoy CR, Xu H, Smith K, Etemadmoghadam D, San Leong H, Choong DY, Byrne DJ, Iravani A, Beck S, Mileshkin L, 2019. Profound MEK inhibitor response in a cutaneous melanoma harboring a GOLGA4-RAF1 fusion. J Clin Invest 129: 1940–1945. 10.1172/JCI123089 30835257 PMC6486352

[GR257246UHRC39] McPherson A, Hormozdiari F, Zayed A, Giuliany R, Ha G, Sun MG, Griffith M, Heravi Moussavi A, Senz J, Melnyk N, 2011. Defuse: an algorithm for gene fusion discovery in tumor RNA-Seq data. PLoS Comput Biol 7: e1001138. 10.1371/journal.pcbi.1001138 21625565 PMC3098195

[GR257246UHRC40] Ng PK, Li J, Jeong KJ, Shao S, Chen H, Tsang YH, Sengupta S, Wang Z, Bhavana VH, Tran R, 2018. Systematic functional annotation of somatic mutations in cancer. Cancer Cell 33: 450–462.e10. 10.1016/j.ccell.2018.01.021 29533785 PMC5926201

[GR257246UHRC41] Nicolle R, Blum Y, Marisa L, Loncle C, Gayet O, Moutardier V, Turrini O, Giovannini M, Bian B, Bigonnet M, 2017. Pancreatic adenocarcinoma therapeutic targets revealed by tumor-stroma cross-talk analyses in patient-derived xenografts. Cell Rep 21: 2458–2470. 10.1016/j.celrep.2017.11.003 29186684 PMC6082139

[GR257246UHRC42] Nicorici D, Satalan M, Edgren H, Kangaspeska S, Murumagi A, Kallioniemi O, Virtanen S, Kilkku O. 2014. FusionCatcher : a tool for finding somatic fusion genes in paired-end RNA-sequencing data. bioRxiv 10.1101/011650

[GR257246UHRC43] Okonechnikov K, Imai-Matsushima A, Paul L, Seitz A, Meyer TF, Garcia-Alcalde F. 2016. Infusion: advancing discovery of fusion genes and chimeric transcripts from deep RNA-sequencing data. PLoS One 11: e0167417. 10.1371/journal.pone.0167417 27907167 PMC5132003

[GR257246UHRC44] R Core Team. 2017. R: a language and environment for statistical computing. R Foundation for Statistical Computing, Vienna. https://www.R-project.org/.

[GR257246UHRC45] Roadmap Epigenomics Consortium, Kundaje A, Meuleman W, Ernst J, Bilenky M, Yen A, Heravi-Moussavi A, Kheradpour P, Zhang Z, Wang J, 2015. Integrative analysis of 111 reference human epigenomes. Nature 518: 317–330. 10.1038/nature14248 25693563 PMC4530010

[GR257246UHRC46] Ross JS, Wang K, Chmielecki J, Gay L, Johnson A, Chudnovsky J, Yelensky R, Lipson D, Ali SM, Elvin JA, 2016. The distribution of *BRAF* gene fusions in solid tumors and response to targeted therapy. Int J Cancer 138: 881–890. 10.1002/ijc.29825 26314551 PMC5049644

[GR257246UHRC47] Roychowdhury S, Iyer MK, Robinson DR, Lonigro RJ, Wu YM, Cao X, Kalyana-Sundaram S, Sam L, Balbin OA, Quist MJ, 2011. Personalized oncology through integrative high-throughput sequencing: a pilot study. Sci Transl Med 3: 111ra121. 10.1126/scitranslmed.3003161 PMC3476478 22133722

[GR257246UHRC48] Schmitz R, Wright GW, Huang DW, Johnson CA, Phelan JD, Wang JQ, Roulland S, Kasbekar M, Young RM, Shaffer AL, 2018. Genetics and pathogenesis of diffuse large B-cell lymphoma. N Engl J Med 378: 1396–1407. 10.1056/NEJMoa1801445 29641966 PMC6010183

[GR257246UHRC49] Schram AM, Chang MT, Jonsson P, Drilon A. 2017. Fusions in solid tumours: diagnostic strategies, targeted therapy, and acquired resistance. Nat Rev Clin Oncol 14: 735–748. 10.1038/nrclinonc.2017.127 28857077 PMC10452928

[GR257246UHRC50] Seshagiri S, Stawiski EW, Durinck S, Modrusan Z, Storm EE, Conboy CB, Chaudhuri S, Guan Y, Janakiraman V, Jaiswal BS, 2012. Recurrent R-spondin fusions in colon cancer. Nature 488: 660–664. 10.1038/nature11282 22895193 PMC3690621

[GR257246UHRC51] Shaw AT, Ou SH, Bang YJ, Camidge DR, Solomon BJ, Salgia R, Riely GJ, Varella-Garcia M, Shapiro GI, Costa DB, 2014. Crizotinib in *ROS1*-rearranged non-small-cell lung cancer. N Engl J Med 371: 1963–1971. 10.1056/NEJMoa1406766 25264305 PMC4264527

[GR257246UHRC52] Stolze B, Reinhart S, Bulllinger L, Fröhling S, Scholl C. 2015. Comparative analysis of KRAS codon 12, 13, 18, 61, and 117 mutations using human MCF10A isogenic cell lines. Sci Rep 5: 8535. 10.1038/srep08535 25705018 PMC4336936

[GR257246UHRC53] Subbiah V, Gainor JF, Rahal R, Brubaker JD, Kim JL, Maynard M, Hu W, Cao Q, Sheets MP, Wilson D, 2018. Precision targeted therapy with BLU-667 for *RET*-driven cancers. Cancer Discov 8: 836–849. 10.1158/2159-8290.CD-18-0338 29657135

[GR257246UHRC54] Suehara Y, Arcila M, Wang L, Hasanovic A, Ang D, Ito T, Kimura Y, Drilon A, Guha U, Rusch V, 2012. Identification of *KIF5B-RET* and *GOPC-ROS1* fusions in lung adenocarcinomas through a comprehensive mRNA-based screen for tyrosine kinase fusions. Clin Cancer Res 18: 6599–6608. 10.1158/1078-0432.CCR-12-0838 23052255 PMC4234119

[GR257246UHRC55] Tannenbaum-Dvir S, Glade Bender JL, Church AJ, Janeway KA, Harris MH, Mansukhani MM, Nagy PL, Andrews SJ, Murty VV, Kadenhe-Chiweshe A, 2015. Characterization of a novel fusion gene *EML4*-*NTRK3* in a case of recurrent congenital fibrosarcoma. Cold Spring Harb Mol Case Stud 1: a000471. 10.1101/mcs.a000471 27148571 PMC4850889

[GR257246UHRC56] Tembe WD, Pond SJ, Legendre C, Chuang HY, Liang WS, Kim NE, Montel V, Wong S, McDaniel TK, Craig DW, 2014. Open-access synthetic spike-in mRNA-seq data for cancer gene fusions. BMC Genomics 15: 824. 10.1186/1471-2164-15-824 25266161 PMC4190330

[GR257246UHRC57] Thorvaldsdottir H, Robinson JT, Mesirov JP. 2013. Integrative Genomics Viewer (IGV): high-performance genomics data visualization and exploration. Brief Bioinform 14: 178–192. 10.1093/bib/bbs017 22517427 PMC3603213

[GR257246UHRC58] Torres-García W, Zheng S, Sivachenko A, Vegesna R, Wang Q, Yao R, Berger MF, Weinstein JN, Getz G, Verhaak RG. 2014. PRADA: pipeline for RNA sequencing data analysis. Bioinformatics 30: 2224–2226. 10.1093/bioinformatics/btu169 24695405 PMC4103589

[GR257246UHRC59] Uhlen M, Fagerberg L, Hallstrom BM, Lindskog C, Oksvold P, Mardinoglu A, Sivertsson A, Kampf C, Sjostedt E, Asplund A, 2015. Proteomics. tissue-based map of the human proteome. Science 347: 1260419. 10.1126/science.1260419 25613900

[GR257246UHRC60] The UniProt Consortium. 2018. UniProt: the universal protein knowledgebase. Nucleic Acids Res 46: 2699. 10.1093/nar/gky092 29425356 PMC5861450

[GR257246UHRC061] Untergasser A, Cutcutache I, Koressaar T, Ye J, Faircloth BC, Remm M, Rozen SG. 2012. Primer3—new capabilities and interfaces. Nucleic Acids Res 40: e115. 10.1093/nar/gks596 22730293 PMC3424584

[GR257246UHRC61] Wang K, Li M, Hakonarson H. 2010. ANNOVAR: functional annotation of genetic variants from high-throughput sequencing data. Nucleic Acids Res 38: e164. 10.1093/nar/gkq603 20601685 PMC2938201

[GR257246UHRC62] Wang J, Vasaikar S, Shi Z, Greer M, Zhang B. 2017. WebGestalt 2017: a more comprehensive, powerful, flexible and interactive gene set enrichment analysis toolkit. Nucleic Acids Res 45: W130–W137. 10.1093/nar/gkx356 28472511 PMC5570149

[GR257246UHRC63] Witkiewicz AK, Balaji U, Eslinger C, McMillan E, Conway W, Posner B, Mills GB, O'Reilly EM, Knudsen ES. 2016. Integrated patient-derived models delineate individualized therapeutic vulnerabilities of pancreatic cancer. Cell Rep 16: 2017–2031. 10.1016/j.celrep.2016.07.023 27498862 PMC5287055

[GR257246UHRC64] Worst BC, van Tilburg CM, Balasubramanian GP, Fiesel P, Witt R, Freitag A, Boudalil M, Previti C, Wolf S, Schmidt S, 2016. Next-generation personalised medicine for high-risk paediatric cancer patients: the INFORM pilot study. Eur J Cancer 65: 91–101. 10.1016/j.ejca.2016.06.009 27479119

