# Supplementary material for: Accurate and efficient detection of gene fusions from RNA sequencing data
Source: Genome Res. 2021 Mar;31(3):448–60. doi: 10.1101/gr.257246.119 (PMC7919457; doi:10.1101/gr.257246.119)
Supplement: Supplemental Material [file supp_31_3_448__index.html]

Accurate and efficient detection of gene fusions from RNA sequencing data — Accurate and efficient detection of gene fusions from RNA sequencing data — Supplemental Material 

# Accurate and efficient detection of gene fusions from RNA sequencing data

## Supplemental Material

- Supplemental\_Figure\_S1.pdf
- Supplemental\_Figure\_S2.pdf
- Supplemental\_Figure\_S3.pdf
- Supplemental\_Figure\_S4.pdf
- Supplemental\_Figure\_S5.pdf
- Supplemental\_Figure\_S6.pdf
- Supplemental\_Figure\_S7.pdf
- Supplemental\_Figure\_S8.pdf
- Supplemental\_Figure\_S9.pdf
- Supplemental\_Figure\_S10.pdf
- Supplemental\_Figure\_S11.pdf
- Supplemental\_Table\_S1.xlsx
- Supplemental\_Table\_S2.xlsx
- Supplemental\_Table\_S3.xlsx
- Supplemental\_Table\_S4.xlsx
- Supplemental\_Table\_S5.xlsx
- Supplemental\_Code\_S1.zip
