# Supplementary material for: Accurate and efficient detection of gene fusions from RNA sequencing data
Source: Genome Res. 2021 Mar;31(3):448–60. doi: 10.1101/gr.257246.119 (PMC7919457; doi:10.1101/gr.257246.119)
Supplement: Supplemental Material [file supp_gr.257246.119_Supplemental_Figure_S1.pdf]

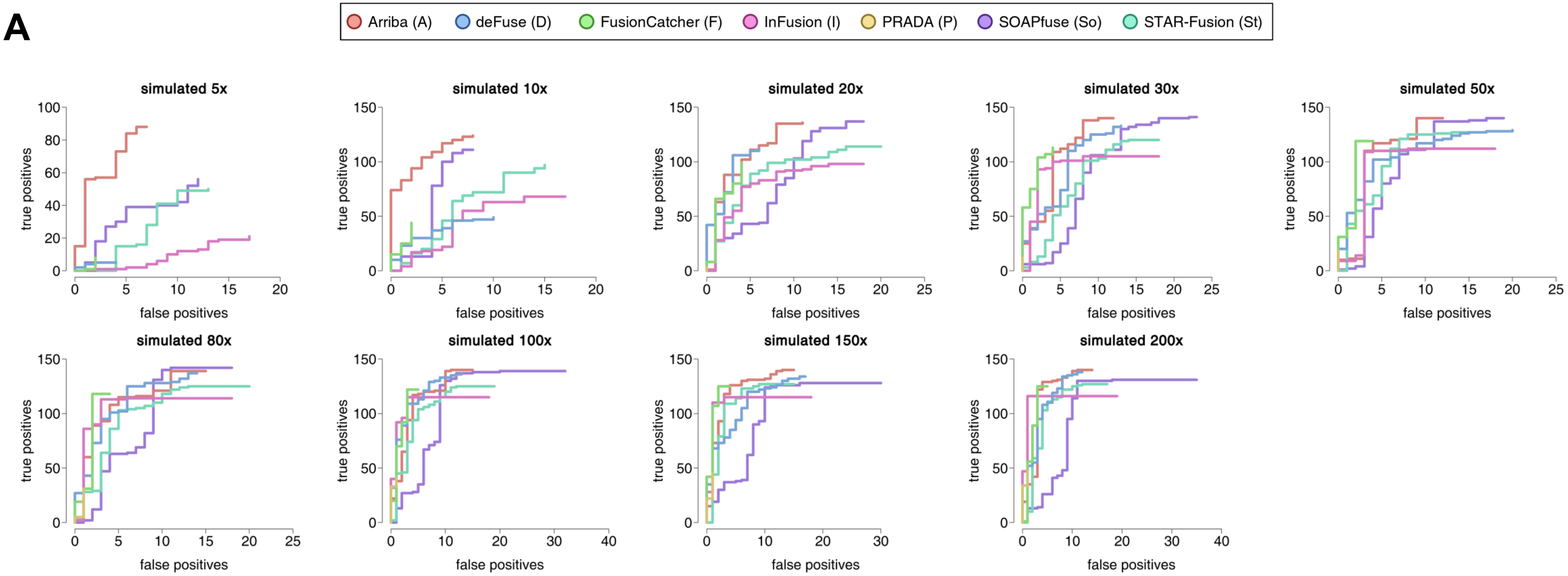
A
Arriba (A)
deFuse (D)
FusionCatcher (F)
InFusion (I)
PRADA (P)
SOAPfuse (So)
STAR-Fusion (St)
simulated 5x
simulated 10x
simulated 20x
simulated 30x
simulated 50x
simulated 80x
simulated 100x
simulated 150x
simulated 200x
true positives
false positives

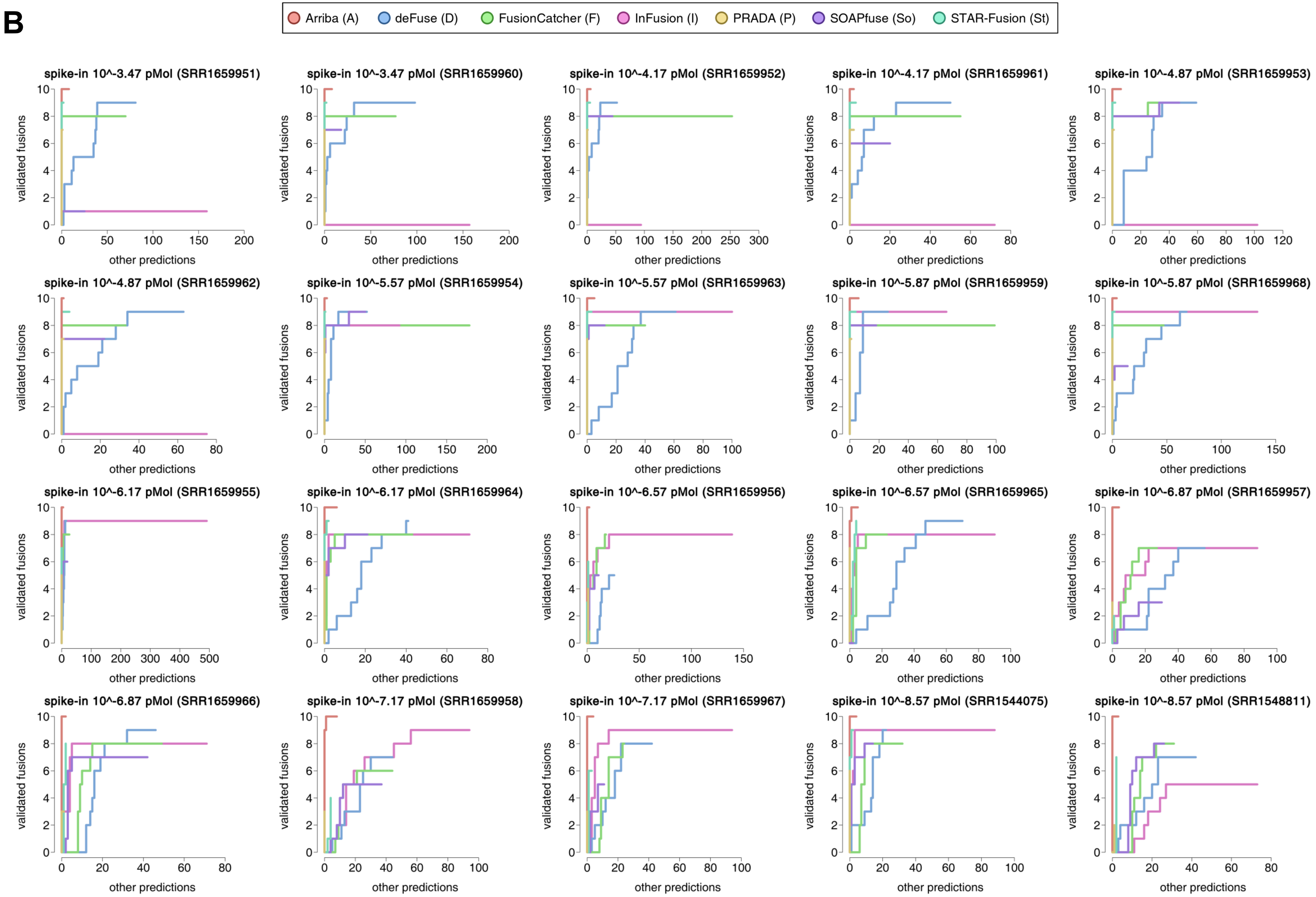
B
Arriba (A)
deFuse (D)
FusionCatcher (F)
InFusion (I)
PRADA (P)
SOAPfuse (So)
STAR-Fusion (St)
spike-in 10^-3.47 pMol (SRR1659951)
spike-in 10^-3.47 pMol (SRR1659960)
spike-in 10^-4.17 pMol (SRR1659952)
spike-in 10^-4.17 pMol (SRR1659961)
spike-in 10^-4.87 pMol (SRR1659953)
spike-in 10^-4.87 pMol (SRR1659962)
spike-in 10^-5.57 pMol (SRR1659954)
spike-in 10^-5.57 pMol (SRR1659963)
spike-in 10^-5.87 pMol (SRR1659959)
spike-in 10^-5.87 pMol (SRR1659968)
spike-in 10^-6.17 pMol (SRR1659955)
spike-in 10^-6.17 pMol (SRR1659964)
spike-in 10^-6.57 pMol (SRR1659956)
spike-in 10^-6.57 pMol (SRR1659965)
spike-in 10^-6.87 pMol (SRR1659957)
spike-in 10^-6.87 pMol (SRR1659966)
spike-in 10^-7.17 pMol (SRR1659958)
spike-in 10^-7.17 pMol (SRR1659967)
spike-in 10^-8.57 pMol (SRR1544075)
spike-in 10^-8.57 pMol (SRR1548811)
validated fusions
other predictions

C

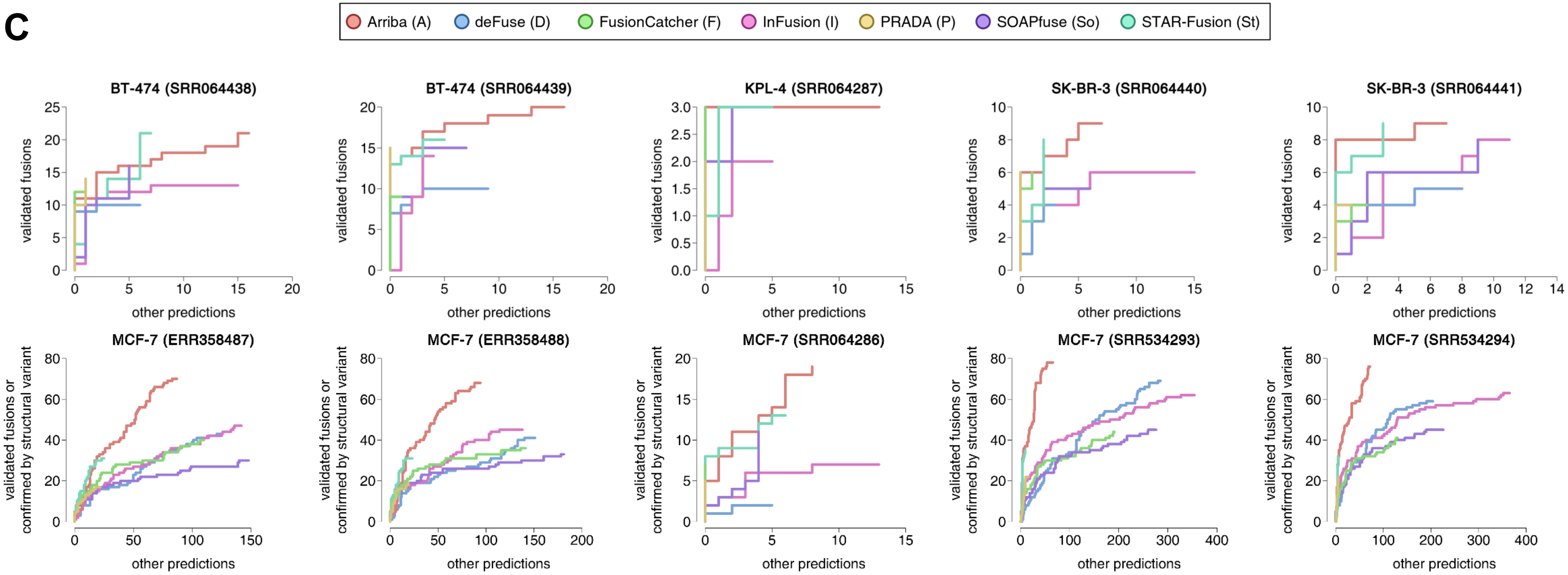


**Supplemental Figure S1: Accuracy of all tools on all benchmark datasets.**
The sensitivity/specificity trade-off is depicted using receiver operating characteristic (ROC)-like curves. The vertical axis indicates the number of true positives; the horizontal axis indicates the number of false positives (simulated dataset) or non-validated predictions (spike-in and cell line datasets). Only gene-to-gene fusions were considered. (A) Simulated data. (B) Spike-ins of synthetic fusions. (C) Cell lines.
