# Supplementary material for: Accurate and efficient detection of gene fusions from RNA sequencing data
Source: Genome Res. 2021 Mar;31(3):448–60. doi: 10.1101/gr.257246.119 (PMC7919457; doi:10.1101/gr.257246.119)
Supplement: Supplemental Material [file supp_gr.257246.119_Supplemental_Figure_S2.pdf]

A

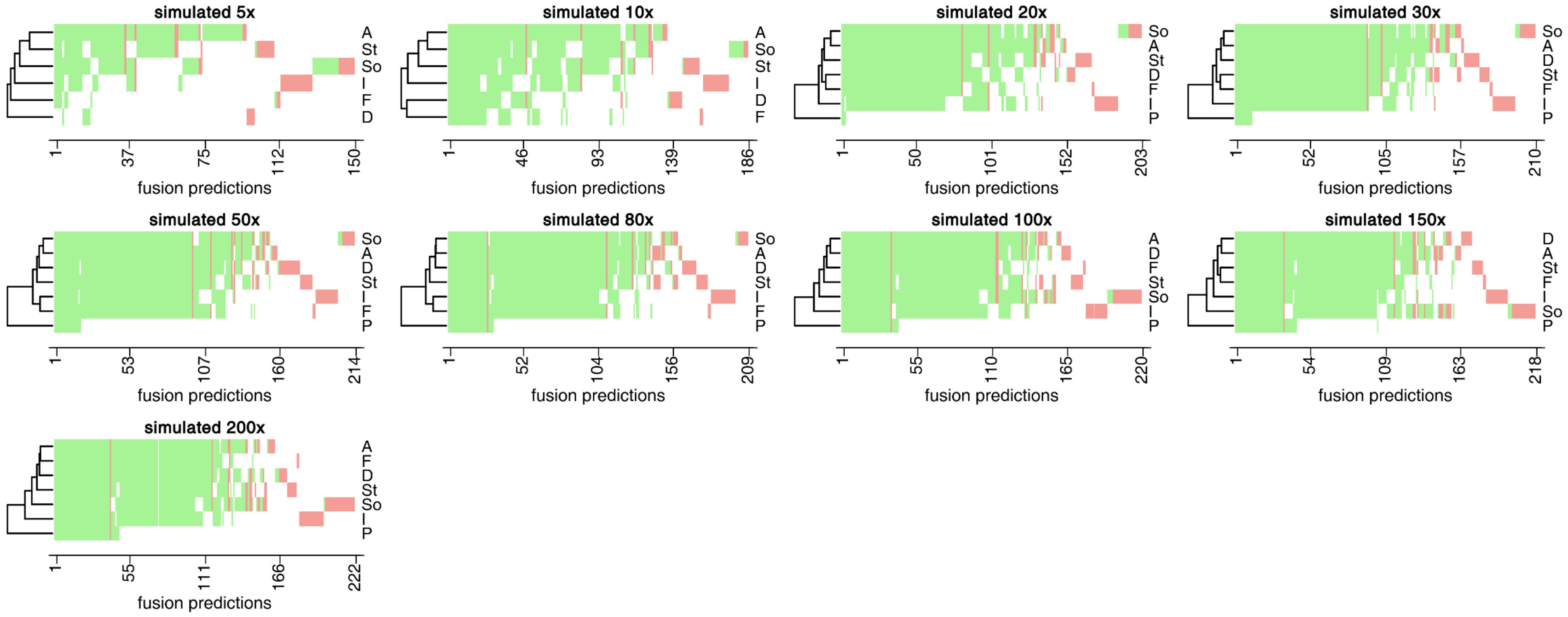
simulated 5x
A St So I F D
1 37 75 112 150
fusion predictions
simulated 10x
A So St I D F
1 46 93 139 186
fusion predictions
simulated 20x
So A St D F I P
1 50 101 152 203
fusion predictions
simulated 30x
So A D St F I P
1 52 105 157 210
fusion predictions
simulated 50x
So A D St I F P
1 53 107 160 214
fusion predictions
simulated 80x
So A D St I F P
1 52 104 156 209
fusion predictions
simulated 100x
A D F St So I P
1 55 110 165 220
fusion predictions
simulated 150x
D A St F I So P
1 54 109 163 218
fusion predictions
simulated 200x
A F D St So I P
1 55 111 166 222
fusion predictions

B

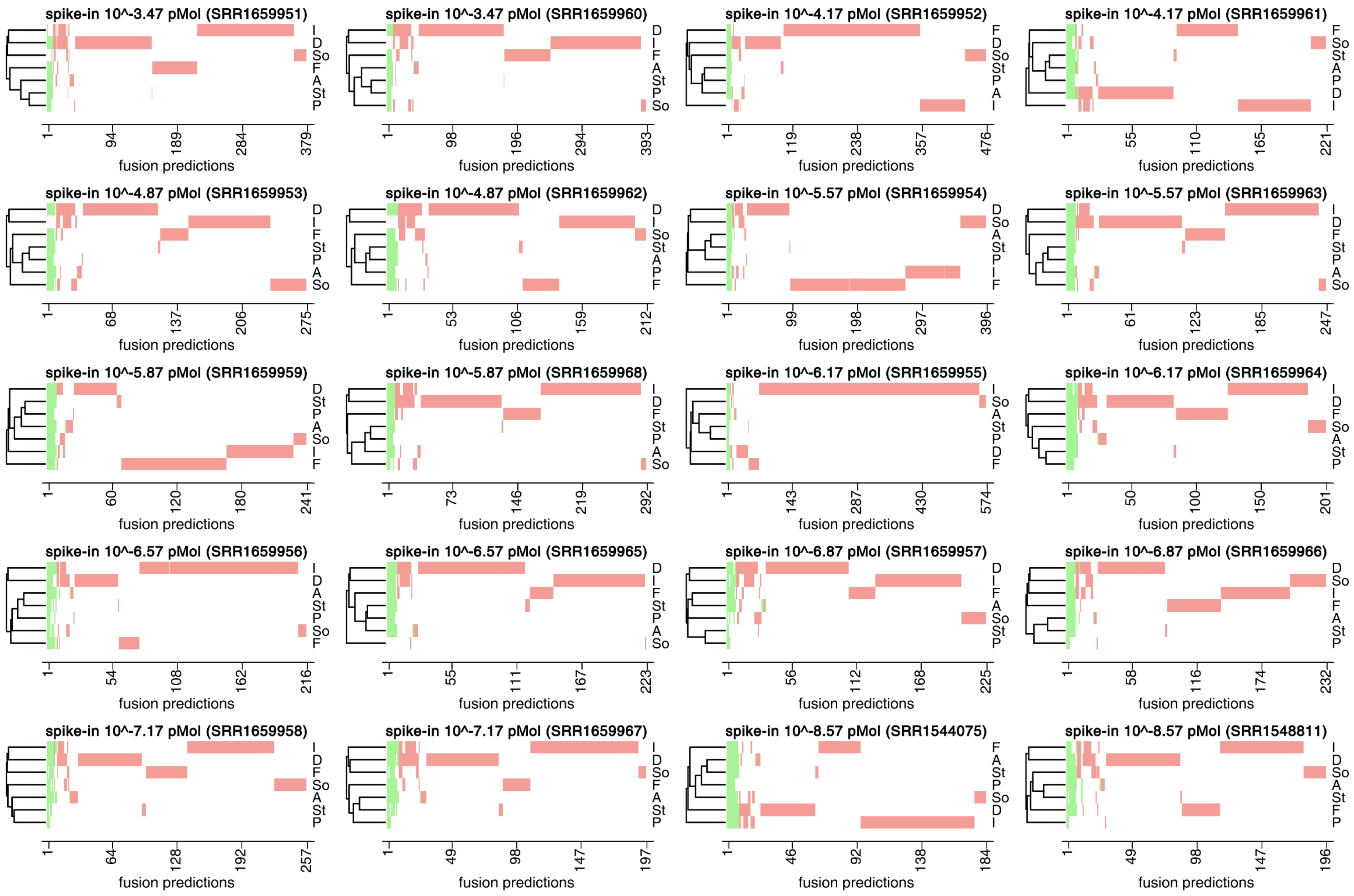

spike-in 10^-3.47 pMol (SRR1659951)
spike-in 10^-3.47 pMol (SRR1659960)
spike-in 10^-4.17 pMol (SRR1659952)
spike-in 10^-4.17 pMol (SRR1659961)
spike-in 10^-4.87 pMol (SRR1659953)
spike-in 10^-4.87 pMol (SRR1659962)
spike-in 10^-5.57 pMol (SRR1659954)
spike-in 10^-5.57 pMol (SRR1659963)
spike-in 10^-5.87 pMol (SRR1659959)
spike-in 10^-5.87 pMol (SRR1659968)
spike-in 10^-6.17 pMol (SRR1659955)
spike-in 10^-6.17 pMol (SRR1659964)
spike-in 10^-6.57 pMol (SRR1659956)
spike-in 10^-6.57 pMol (SRR1659965)
spike-in 10^-6.87 pMol (SRR1659957)
spike-in 10^-6.87 pMol (SRR1659966)
spike-in 10^-7.17 pMol (SRR1659958)
spike-in 10^-7.17 pMol (SRR1659967)
spike-in 10^-8.57 pMol (SRR1544075)
spike-in 10^-8.57 pMol (SRR1548811)
fusion predictions

C

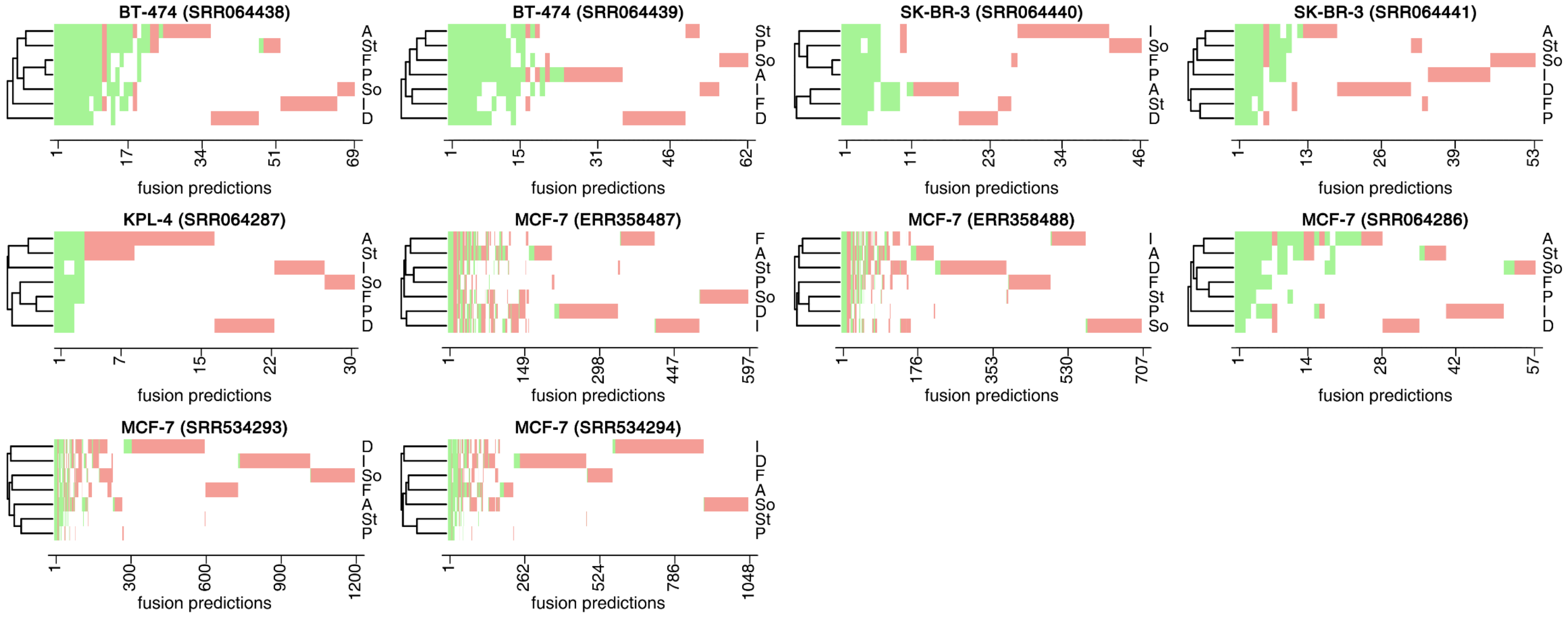

Supplemental Figure S2: Fusion predictions shared between the methods.
The fusion predictions are sorted in descending order from left to right by the number of methods that reported them. True positives are shown in green; false positives (simulated dataset) or non-validated predictions (spike-in and cell line datasets) are shown in red. Only gene-to-gene fusions were considered. A, Arriba; D, deFuse; F, FusionCatcher; I, InFusion; P, PRADA; So, SOAPfuse; St, STAR-Fusion. (A) Simulated data. (B) Spike-ins of synthetic fusions. (C) Cell lines.
