# Supplementary material for: Accurate and efficient detection of gene fusions from RNA sequencing data
Source: Genome Res. 2021 Mar;31(3):448–60. doi: 10.1101/gr.257246.119 (PMC7919457; doi:10.1101/gr.257246.119)
Supplement: Supplemental Material [file supp_gr.257246.119_Supplemental_Figure_S3.pdf]

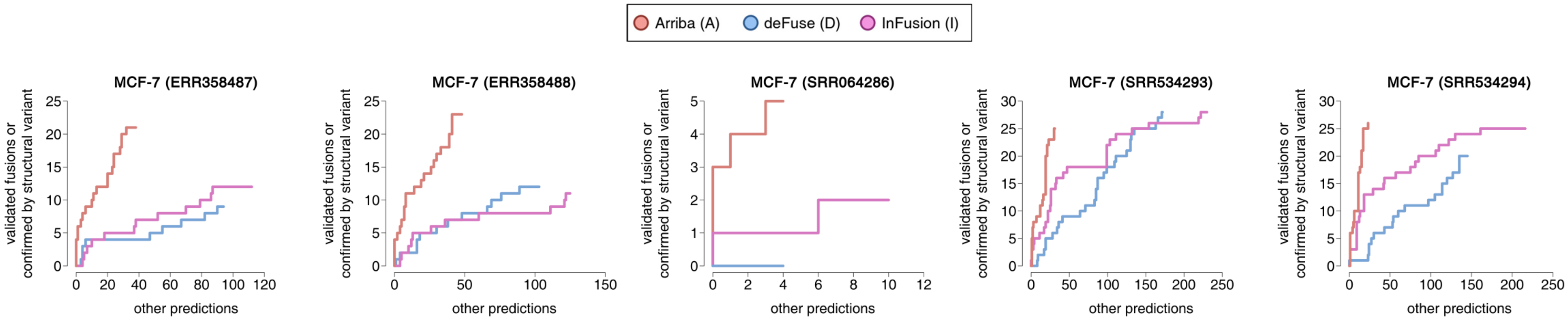

Supplemental Figure S3: Accuracy of predictions of fusions with intergenic breakpoints.
The sensitivity/specificity trade-off is depicted using receiver operating characteristic (ROC)-like curves. The vertical axis indicates the number of predictions confirmed by wet-lab experiments or structural variants; the horizontal axis indicates the number of unconfirmed predictions. Only tools that are capable of detecting fusions with intergenic breakpoints were included (Arriba, deFuse, InFusion).
