# Supplementary material for: Accurate and efficient detection of gene fusions from RNA sequencing data
Source: Genome Res. 2021 Mar;31(3):448–60. doi: 10.1101/gr.257246.119 (PMC7919457; doi:10.1101/gr.257246.119)
Supplement: Supplemental Material [file supp_gr.257246.119_Supplemental_Figure_S6.pdf]

# CFPAC-1 (9fc5da43-4f04-4bdc-a5bb-59a1ec219926)

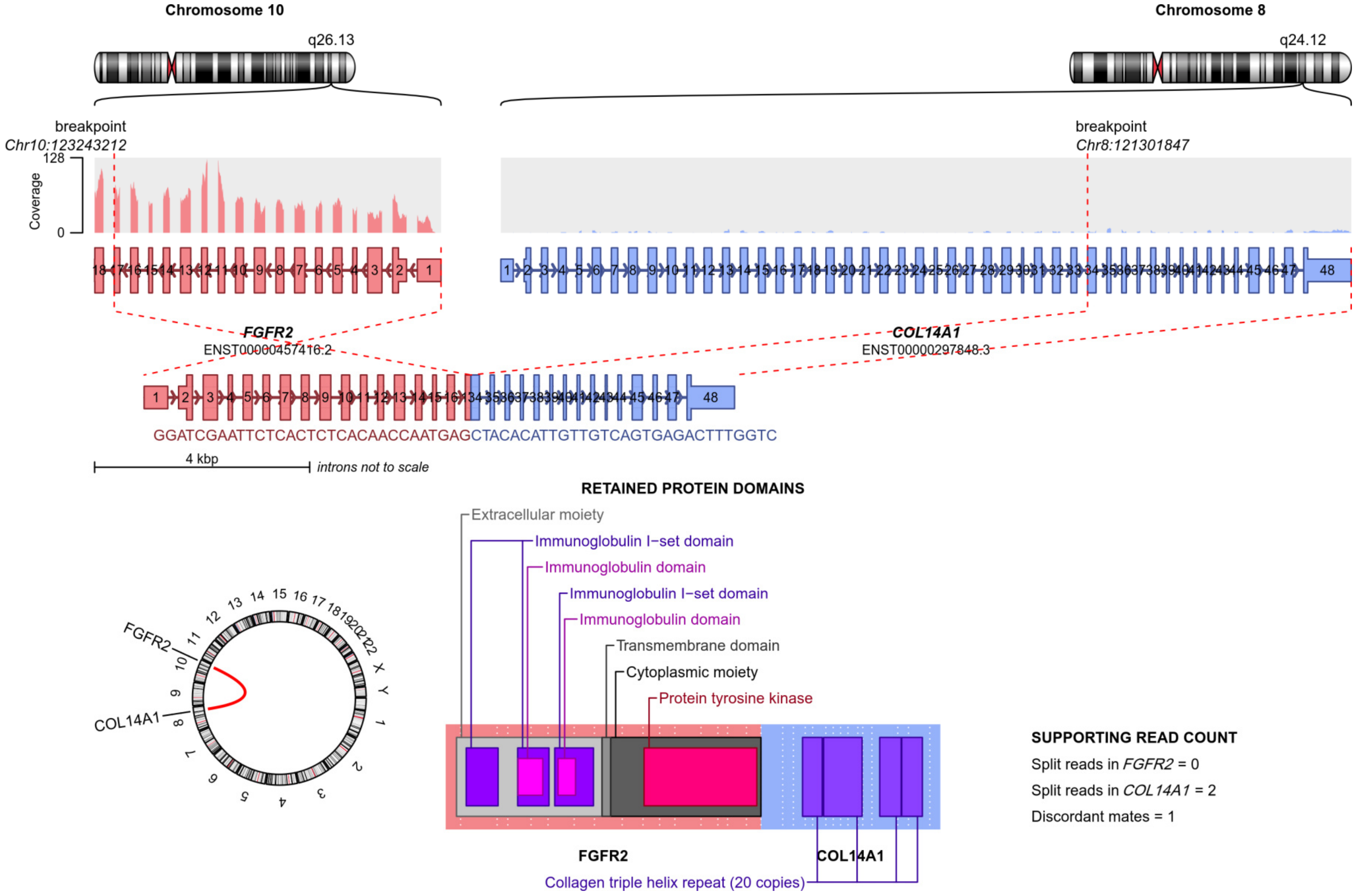

# HPAC (59b5010b-df6b-4d72-9538-350c4cd6062a)

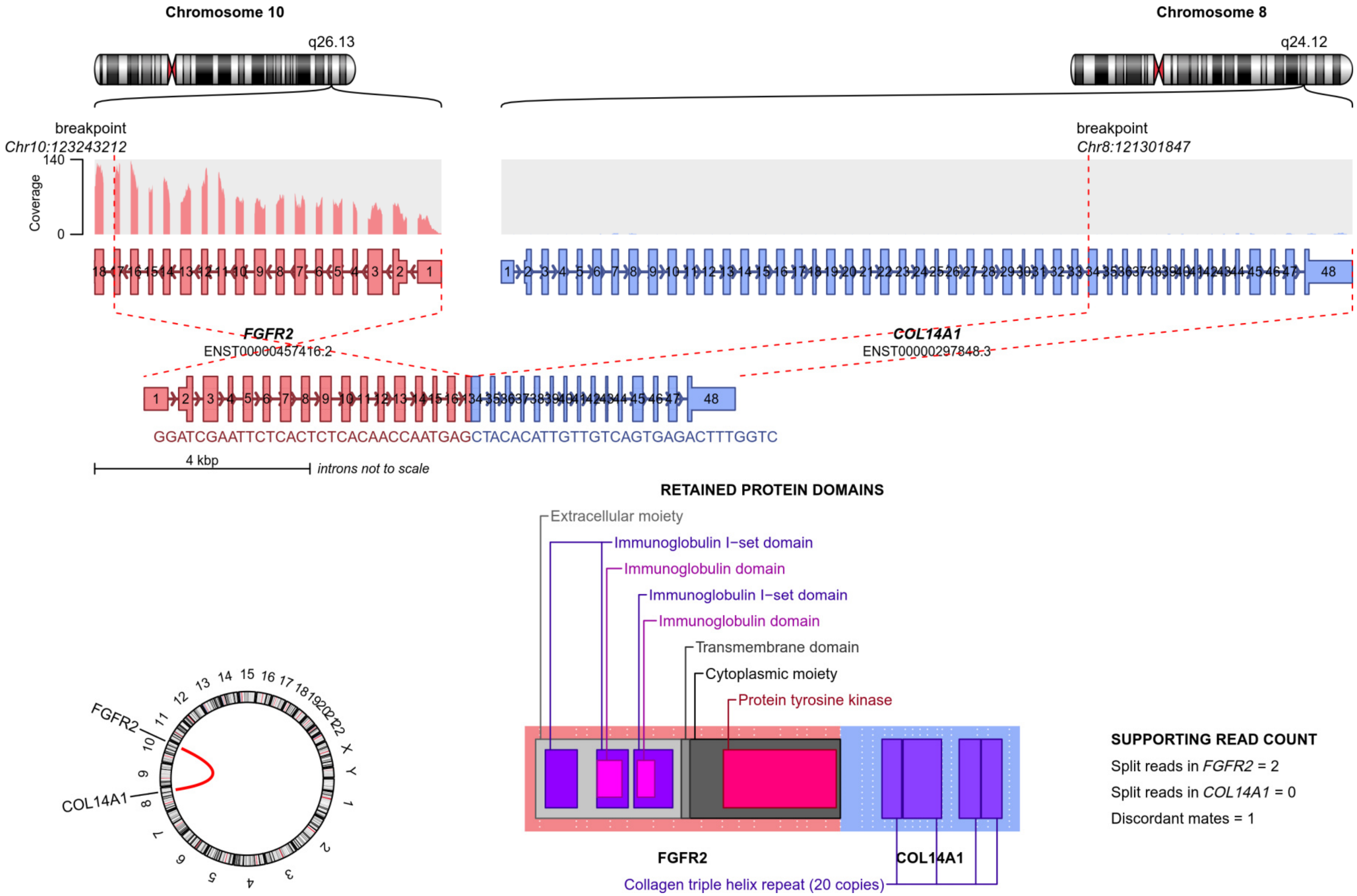


**SUPPORTING READ COUNT**

Split reads in *FGFR2* = 2

Split reads in *COL14A1* = 0

Discordant mates = 1

## SNU-324 (13924031-19ce-4fcb-b023-5b2e3aefdf77)

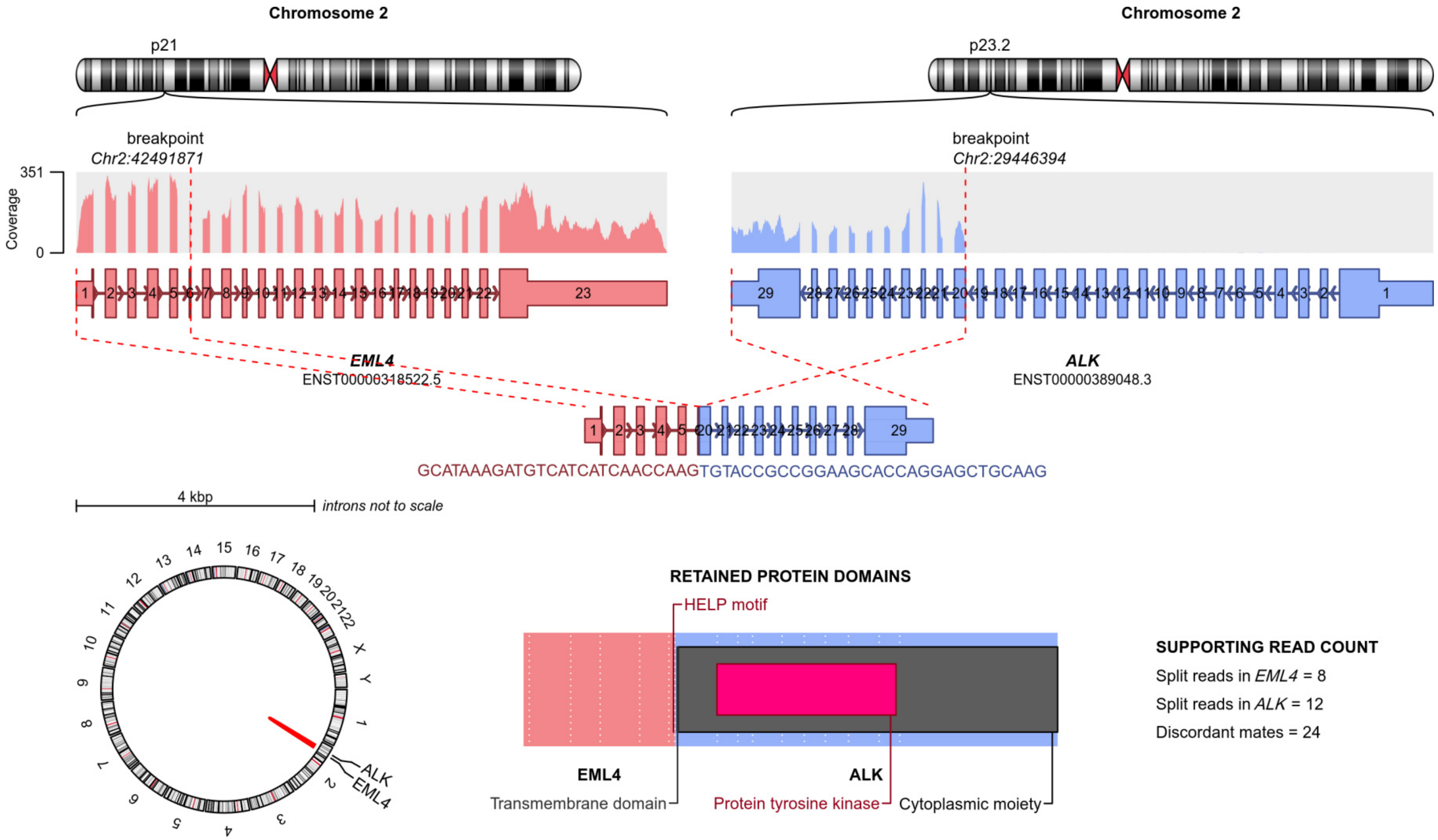


**SUPPORTING READ COUNT**

Split reads in *EML4* = 8

Split reads in *ALK* = 12

Discordant mates = 24

## COMP-0002 (EGAF00001725478, EGAF00001745325, EGAF00001745326)

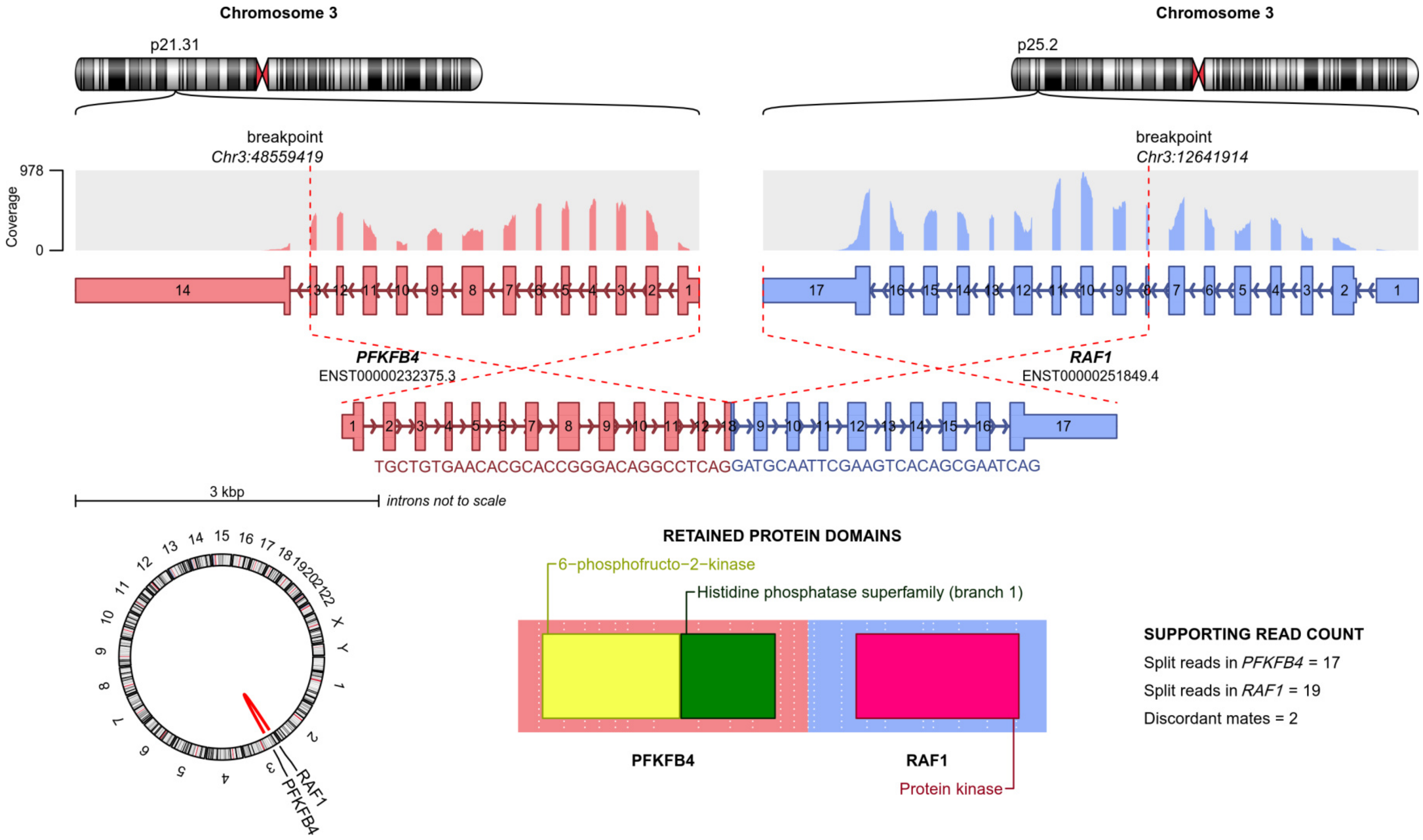


**SUPPORTING READ COUNT**

Split reads in *PFKFB4* = 17

Split reads in *RAF1* = 19

Discordant mates = 2

## COMP-0023 (EGAF00001725495, EGAF00001745371, EGAF00001745372)

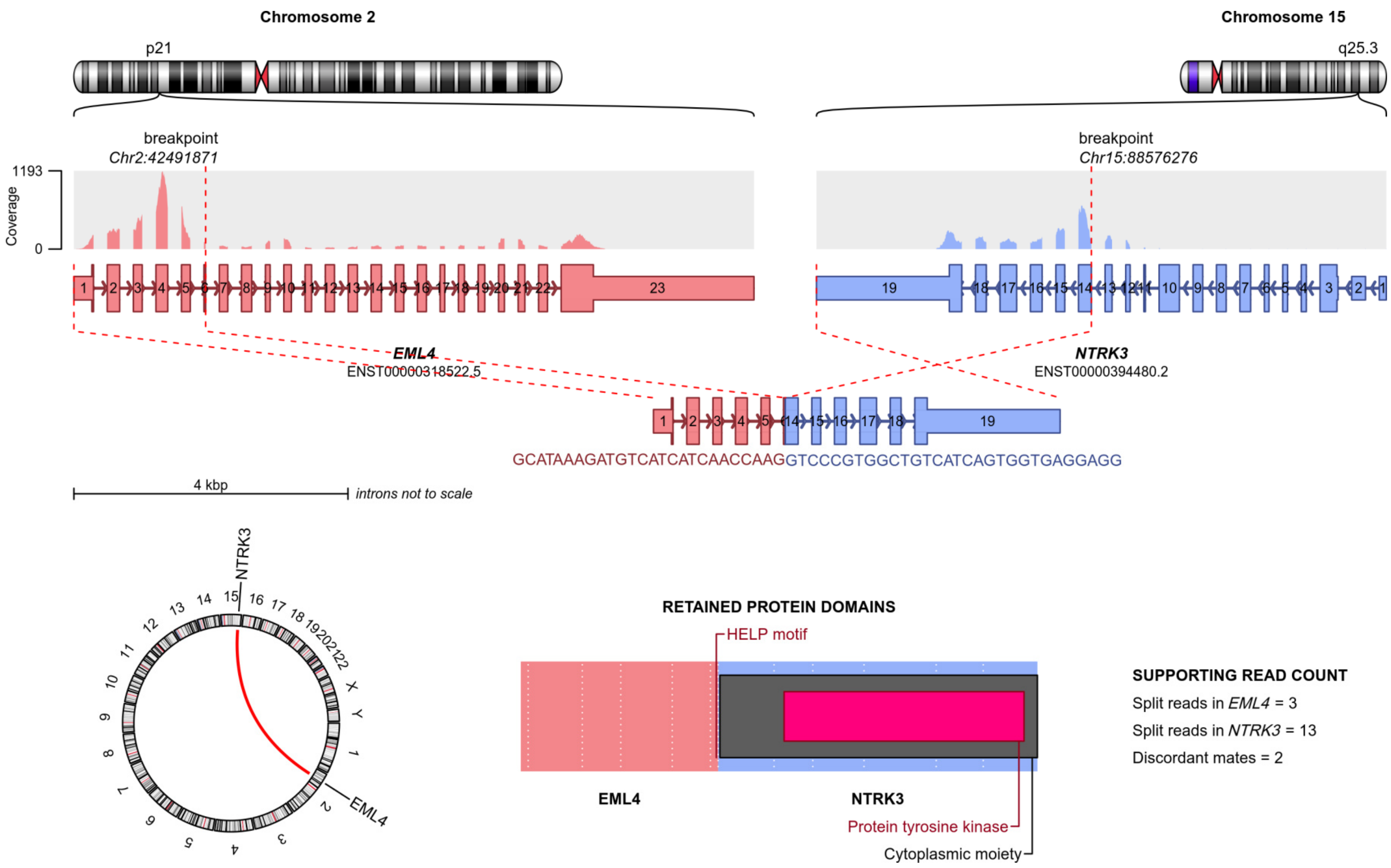


**SUPPORTING READ COUNT**

Split reads in *EML4* = 3

Split reads in *NTRK3* = 13

Discordant mates = 2

## COMP-0028 (EGAF00001725498, EGAF00001745377, EGAF00001745378)

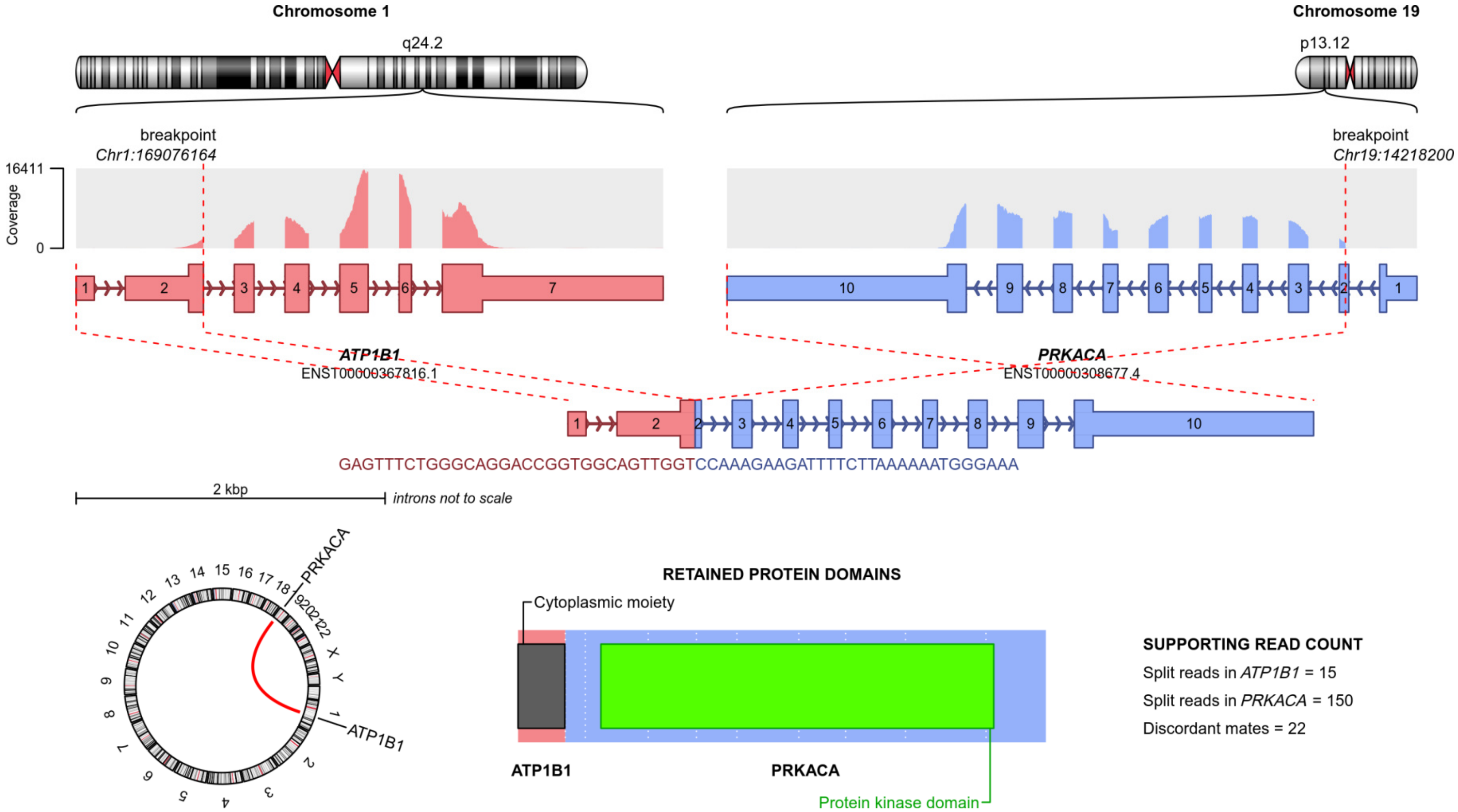

# COMP-0055 (EGAF00001725520, EGAF00001745345, EGAF00001745346)

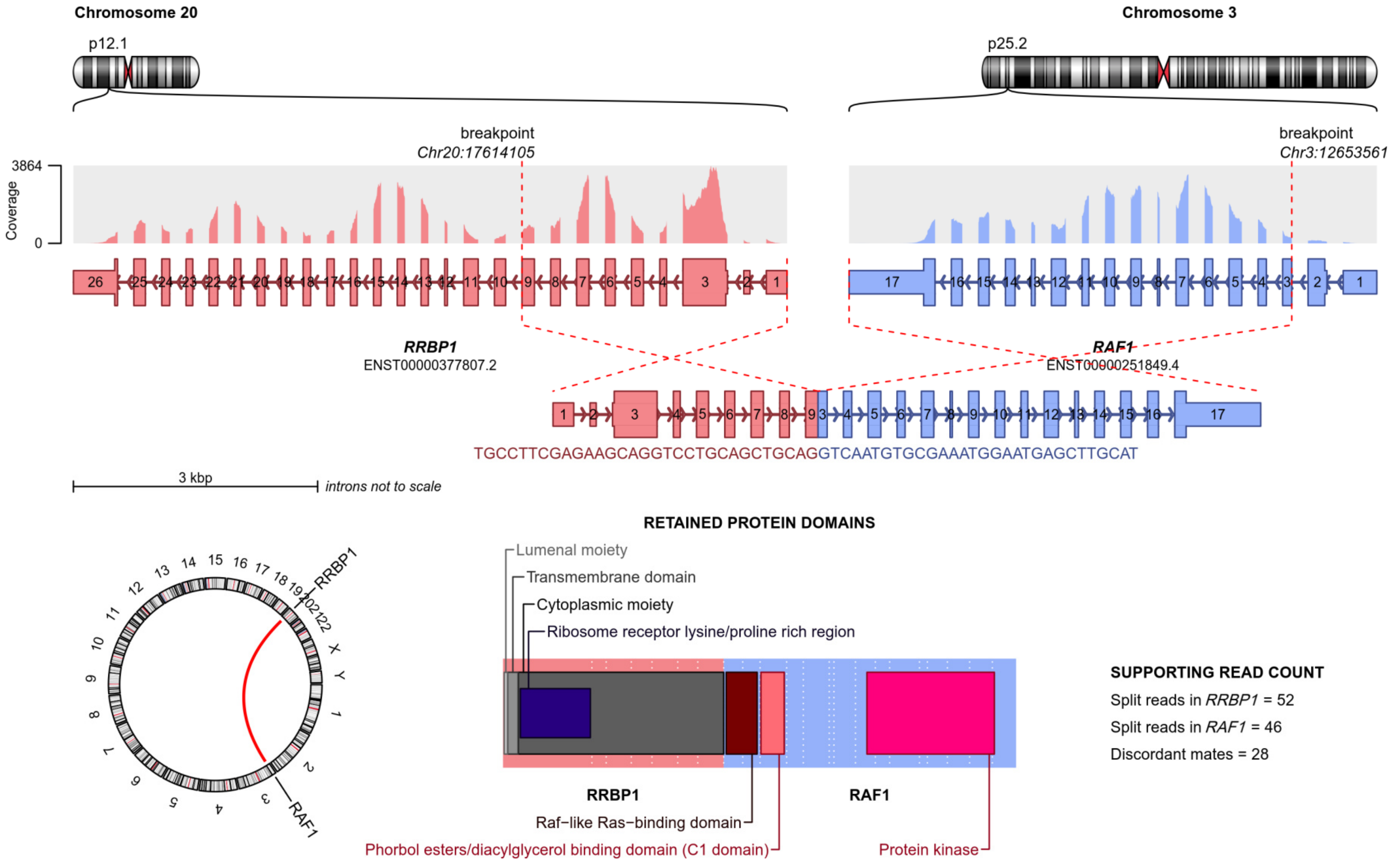

# T_05_08_A230b (SRR3308904)

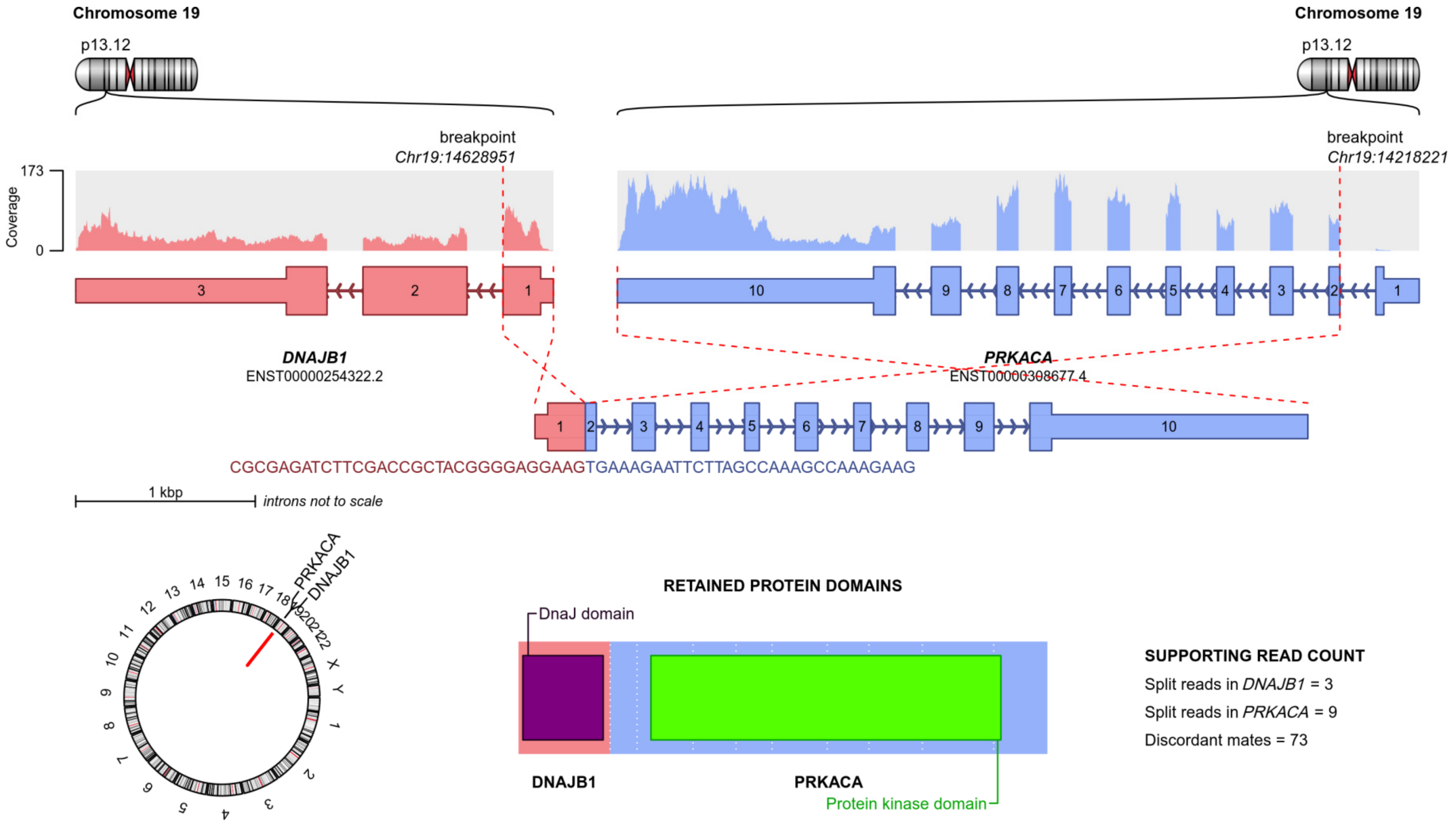

## T_07_06_A323a (SRR3308914)

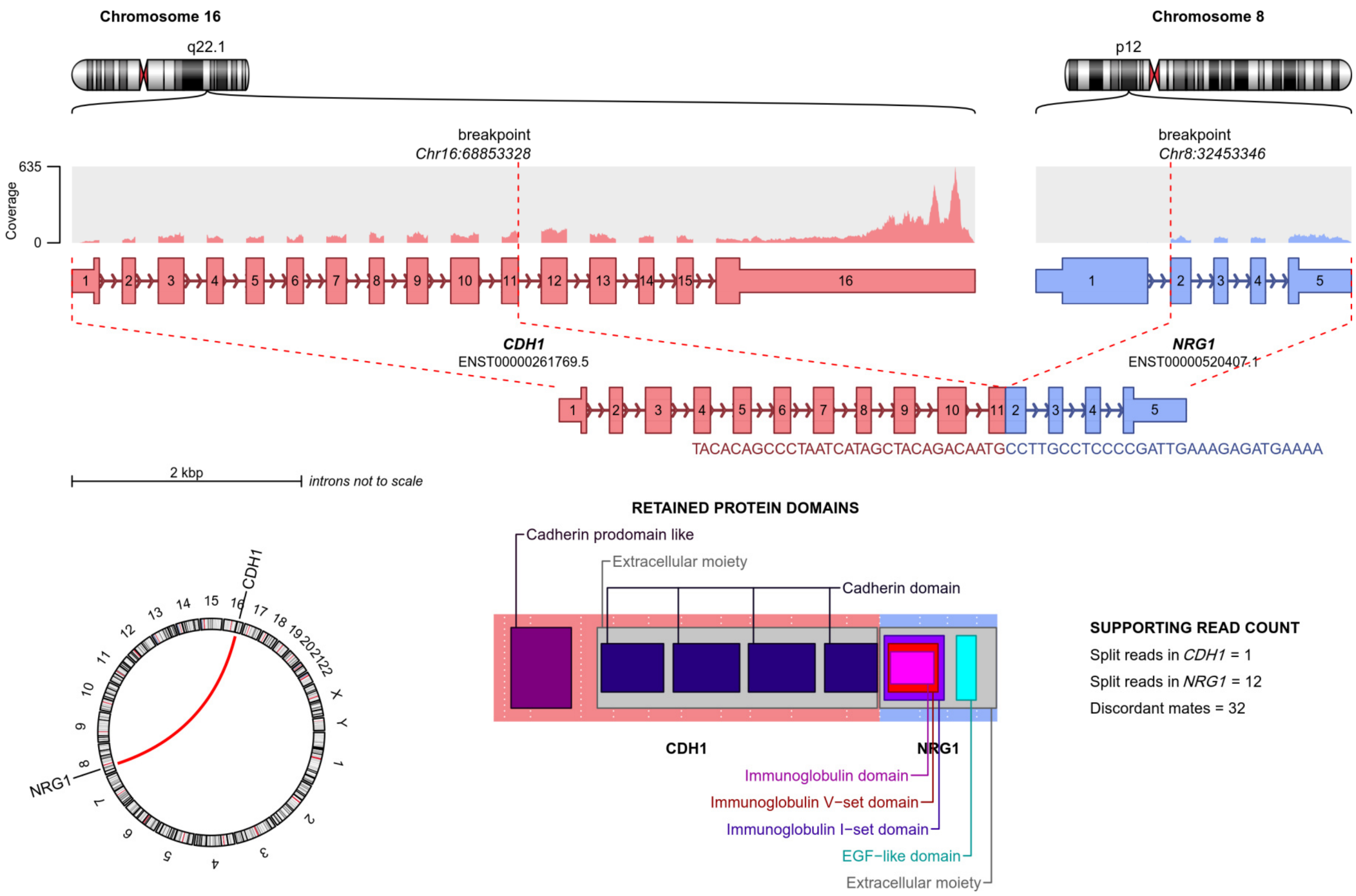


**SUPPORTING READ COUNT**

Split reads in *CDH1* = 1

Split reads in *NRG1* = 12

Discordant mates = 32

## T_07_11_A090a (SRR3308887)

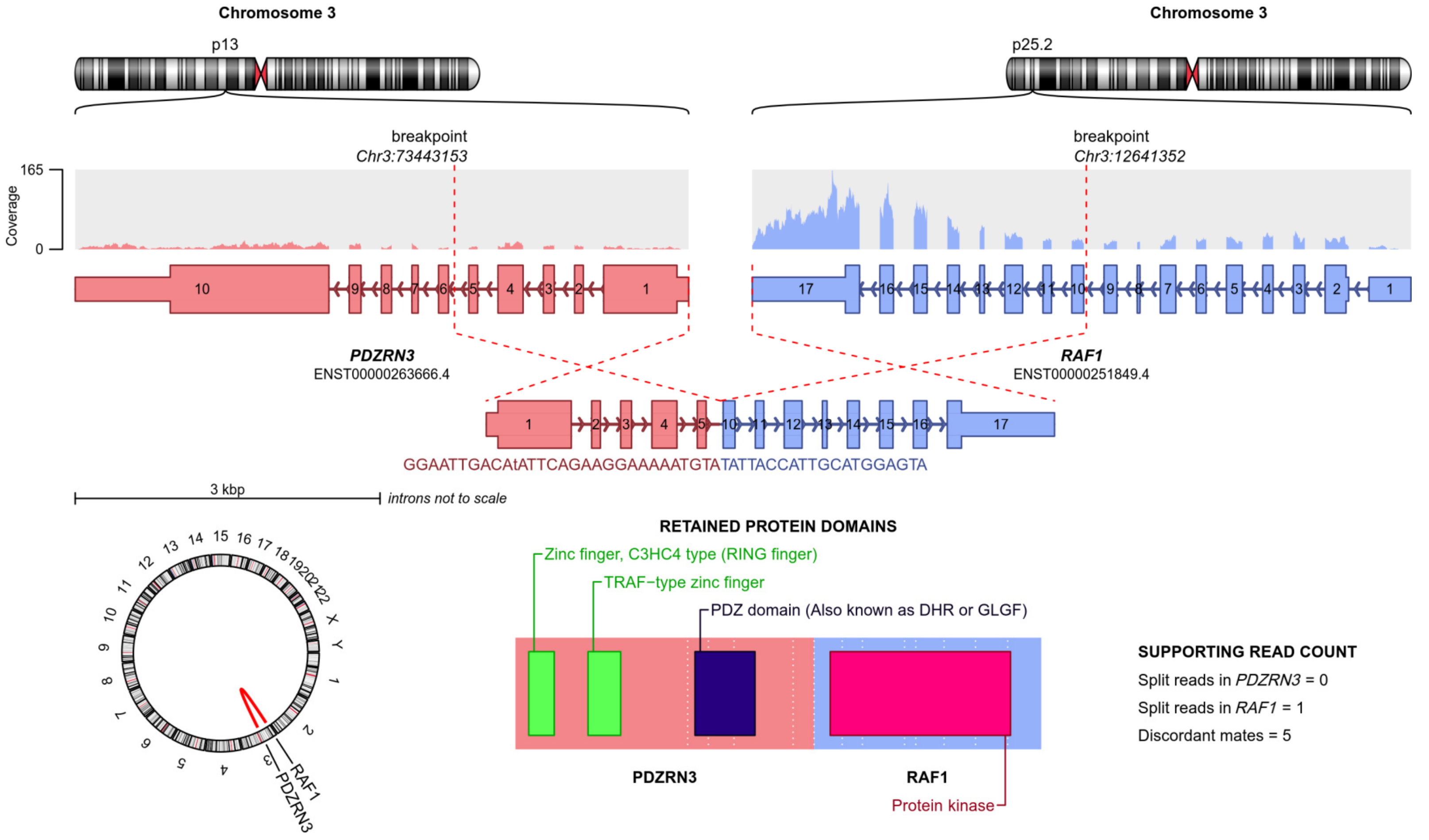

# Patient 15 (EGAF00001984744)

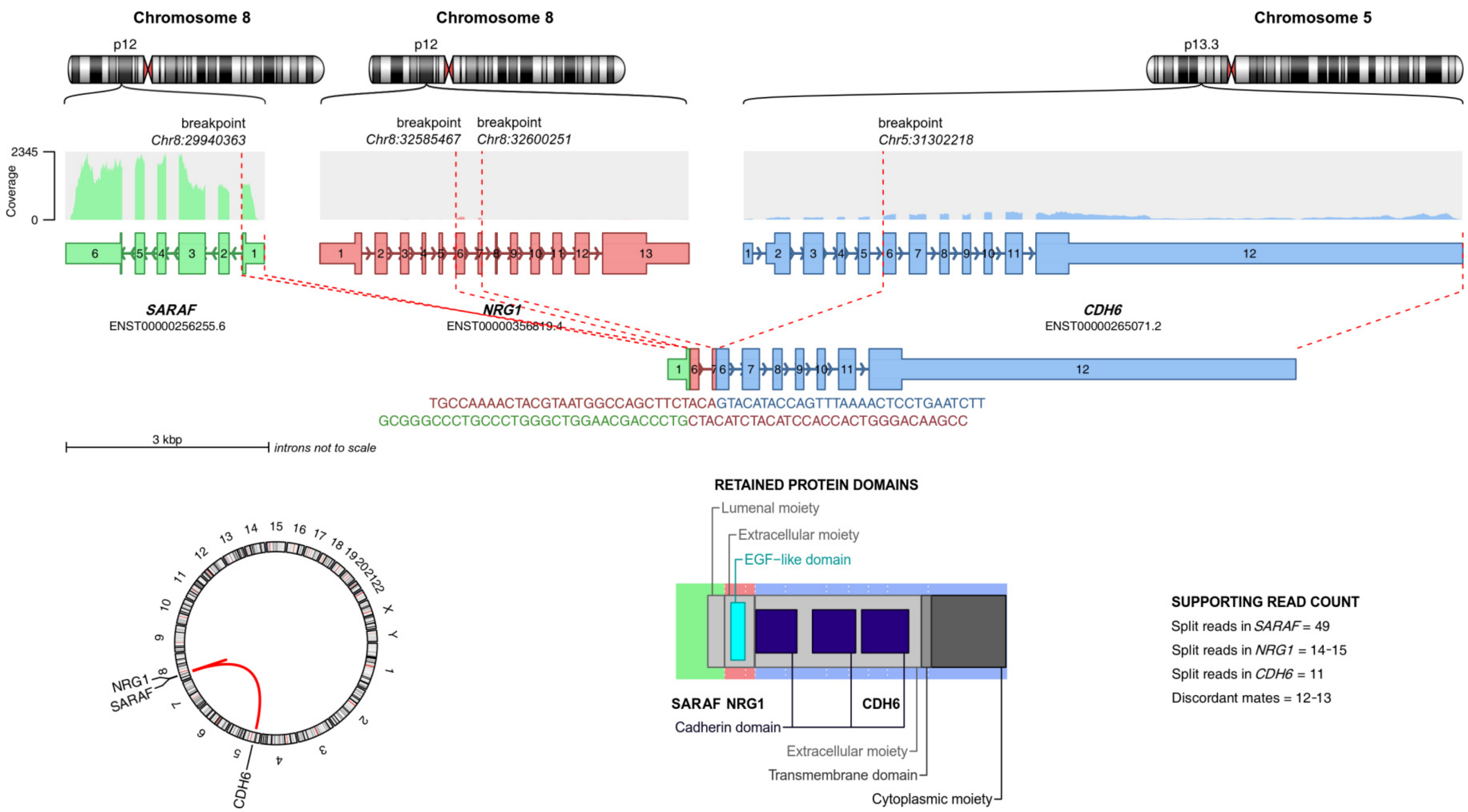


**SUPPORTING READ COUNT**

Split reads in *SARAF* = 49

Split reads in *NRG1* = 14-15

Split reads in *CDH6* = 11

Discordant mates = 12-13

## Patient 14 (EGAF00001984739)

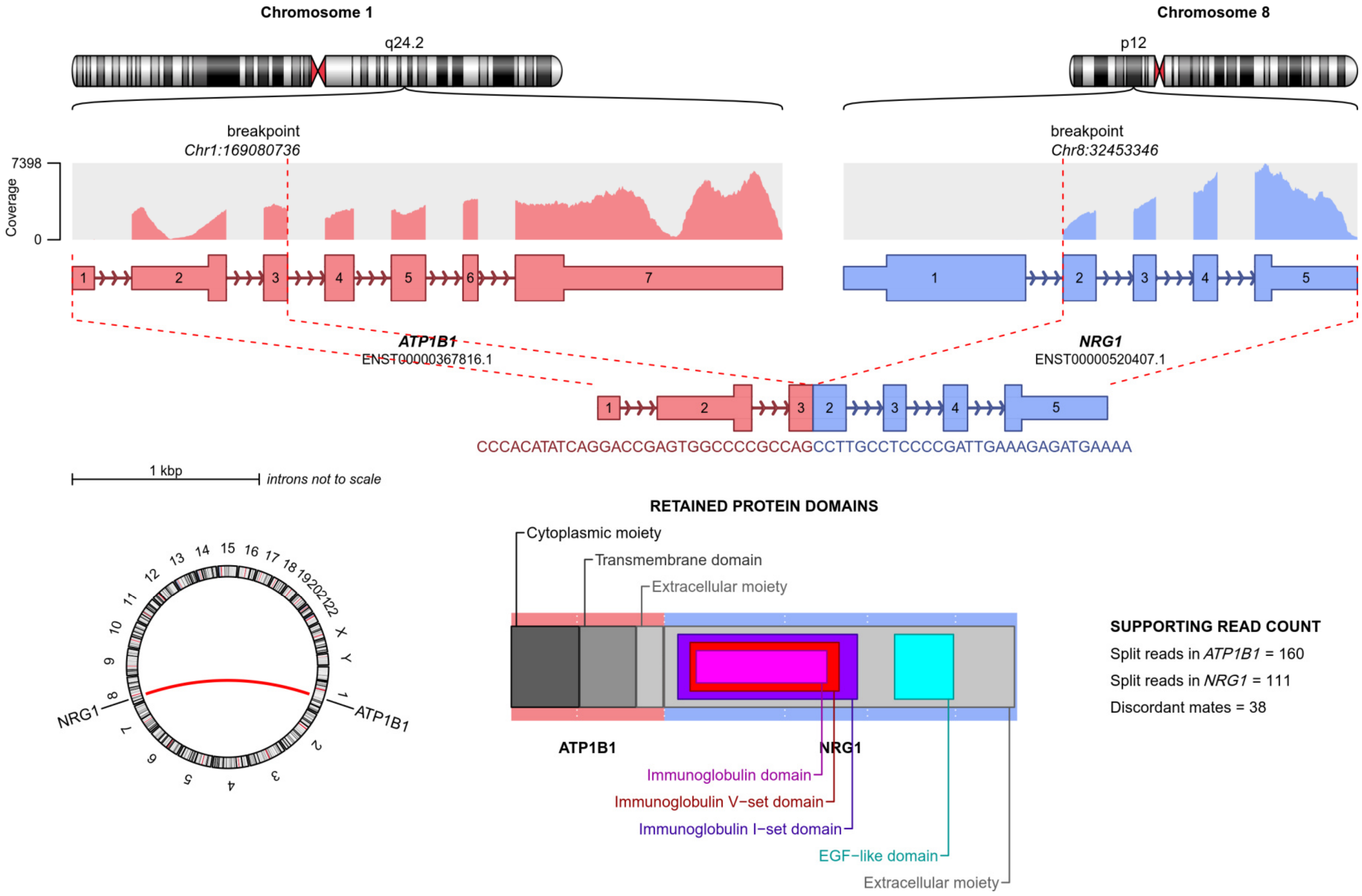

# Patient 17 (EGAF00001984730)

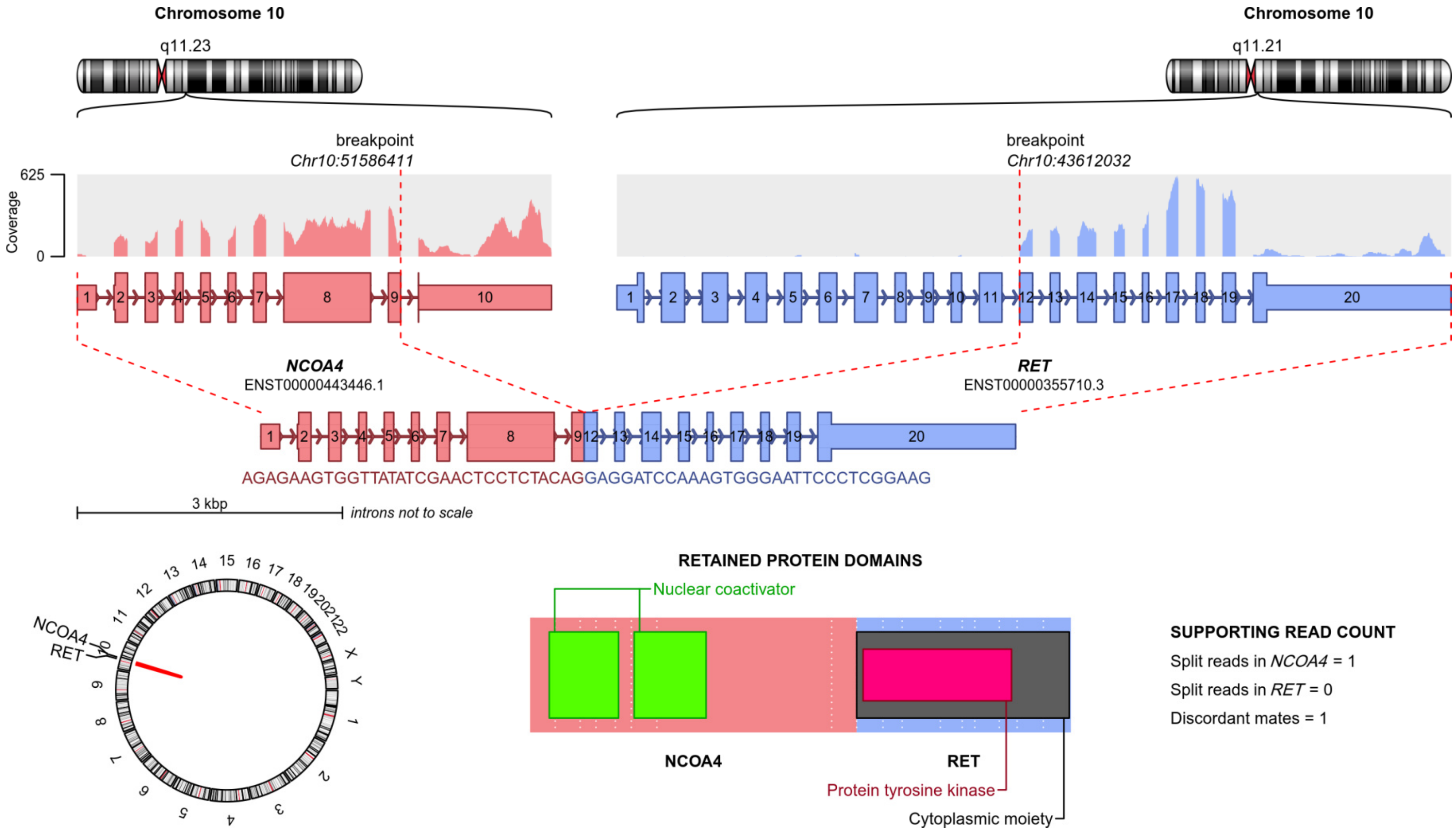

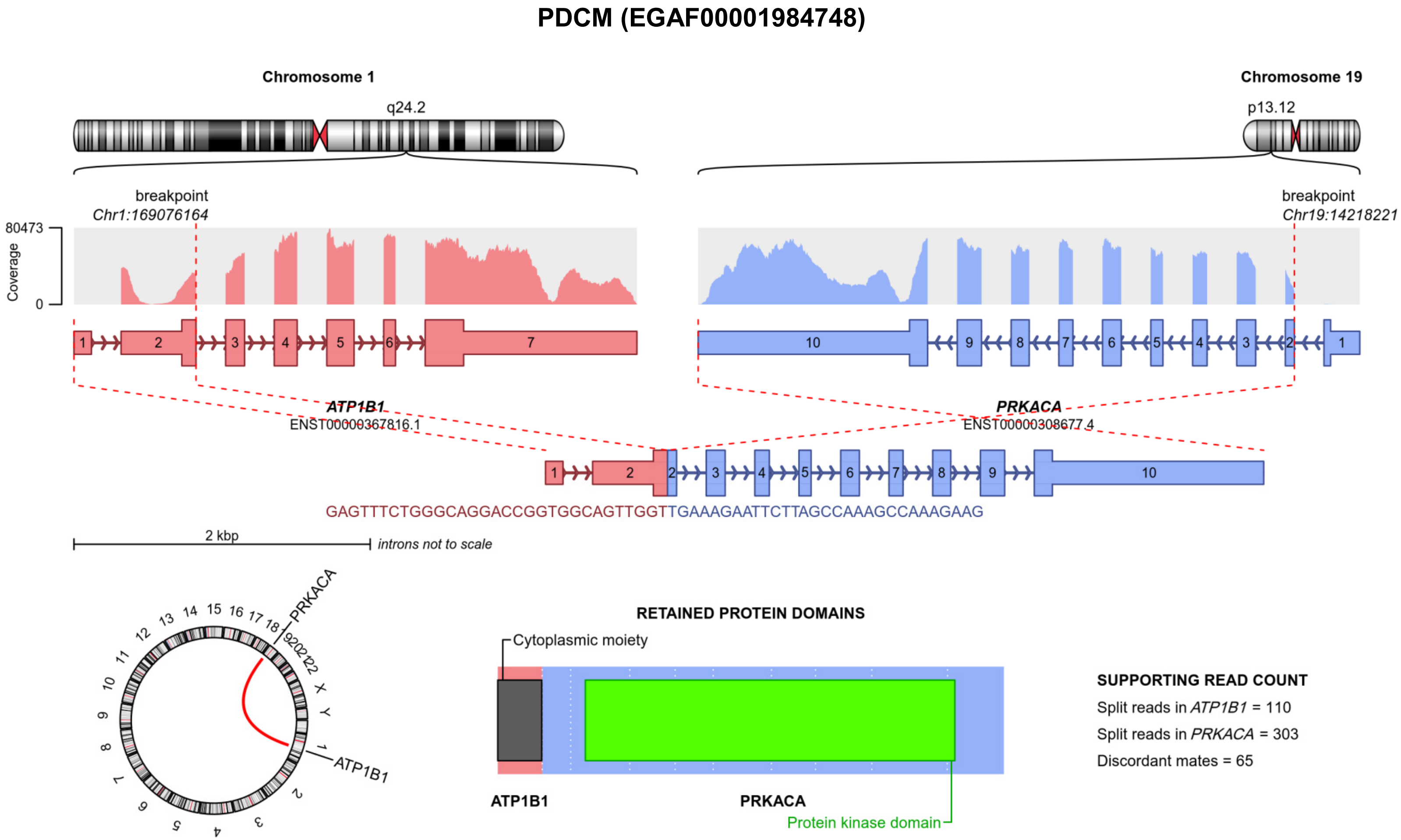
PDCM (EGAF00001984748)
Chromosome 1
q24.2
Chromosome 19
p13.12
breakpoint
Chr1:169076164
breakpoint
Chr19:14218221
80473
Coverage
0
ATP1B1
ENST00000367816.1
PRKACA
ENST00000308677.4
GAGTTTCTGGGCAGGACCGGTGGCAGTTGGTTGAAAGAATTCTTAGCCAAAGCCAAAGAAG
2 kbp
introns not to scale
PRKACA
ATP1B1
RETAINED PROTEIN DOMAINS
Cytoplasmic moiety
ATP1B1
PRKACA
Protein kinase domain
SUPPORTING READ COUNT
Split reads in ATP1B1 = 110
Split reads in PRKACA = 303
Discordant mates = 65

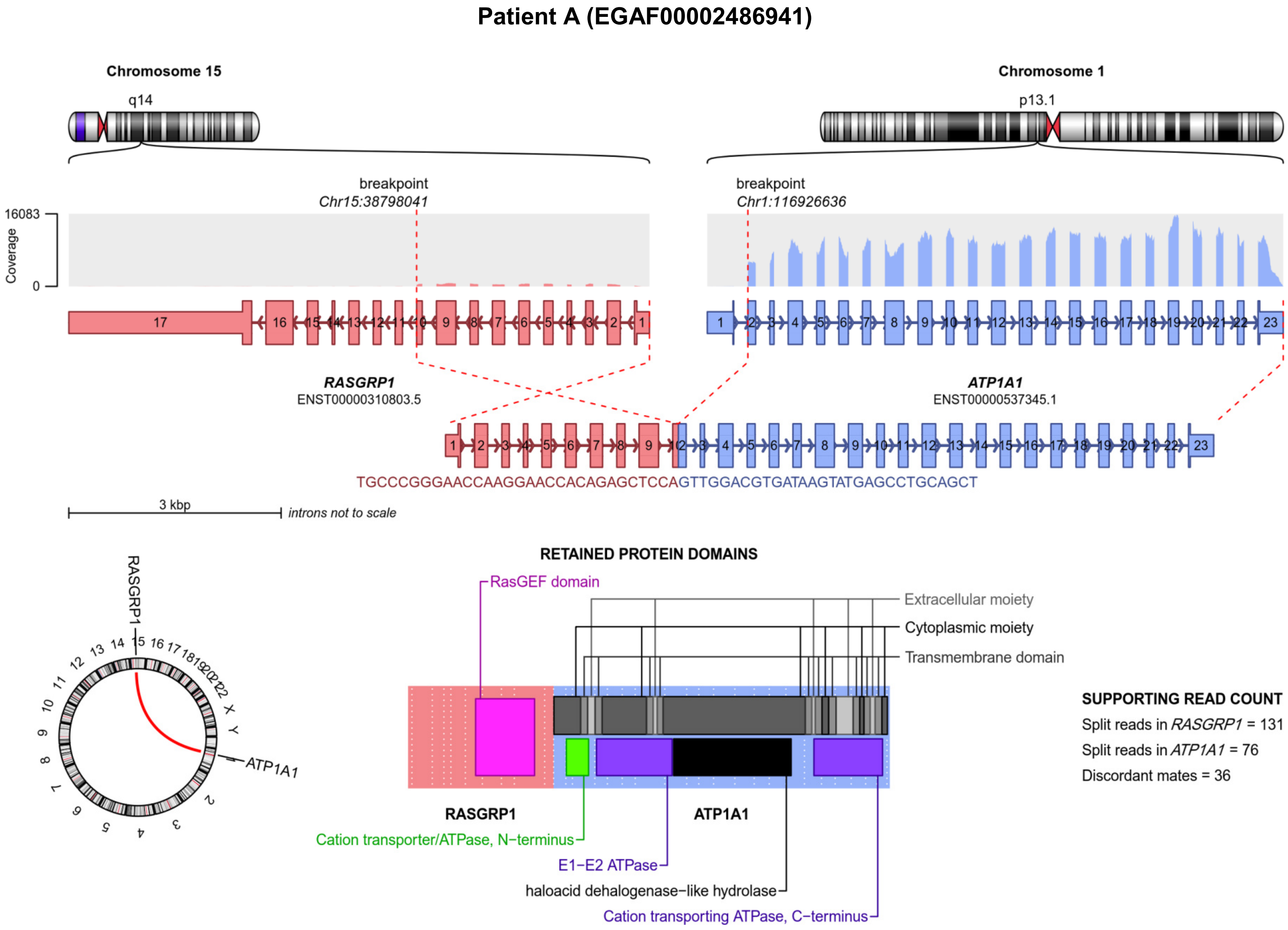
Patient A (EGAF00002486941)
Chromosome 15
q14
Chromosome 1
p13.1
breakpoint
Chr15:38798041
breakpoint
Chr1:116926636
16083
Coverage
0
RASGRP1
ENST00000310803.5
ATP1A1
ENST00000537345.1
TGCCCGGGAACCAAGGAACCACAGAGCTCCAGTTGGACGTGATAAGTATGAGCCTGCAGCT
3 kbp
introns not to scale
RETAINED PROTEIN DOMAINS
RasGEF domain
Extracellular moiety
Cytoplasmic moiety
Transmembrane domain
RASGRP1
ATP1A1
Cation transporter/ATPase, N−terminus
E1−E2 ATPase
haloacid dehalogenase−like hydrolase
Cation transporting ATPase, C−terminus
SUPPORTING READ COUNT
Split reads in RASGRP1 = 131
Split reads in ATP1A1 = 76
Discordant mates = 36

## PDAC009T (ERR1880131, ERR1880132)

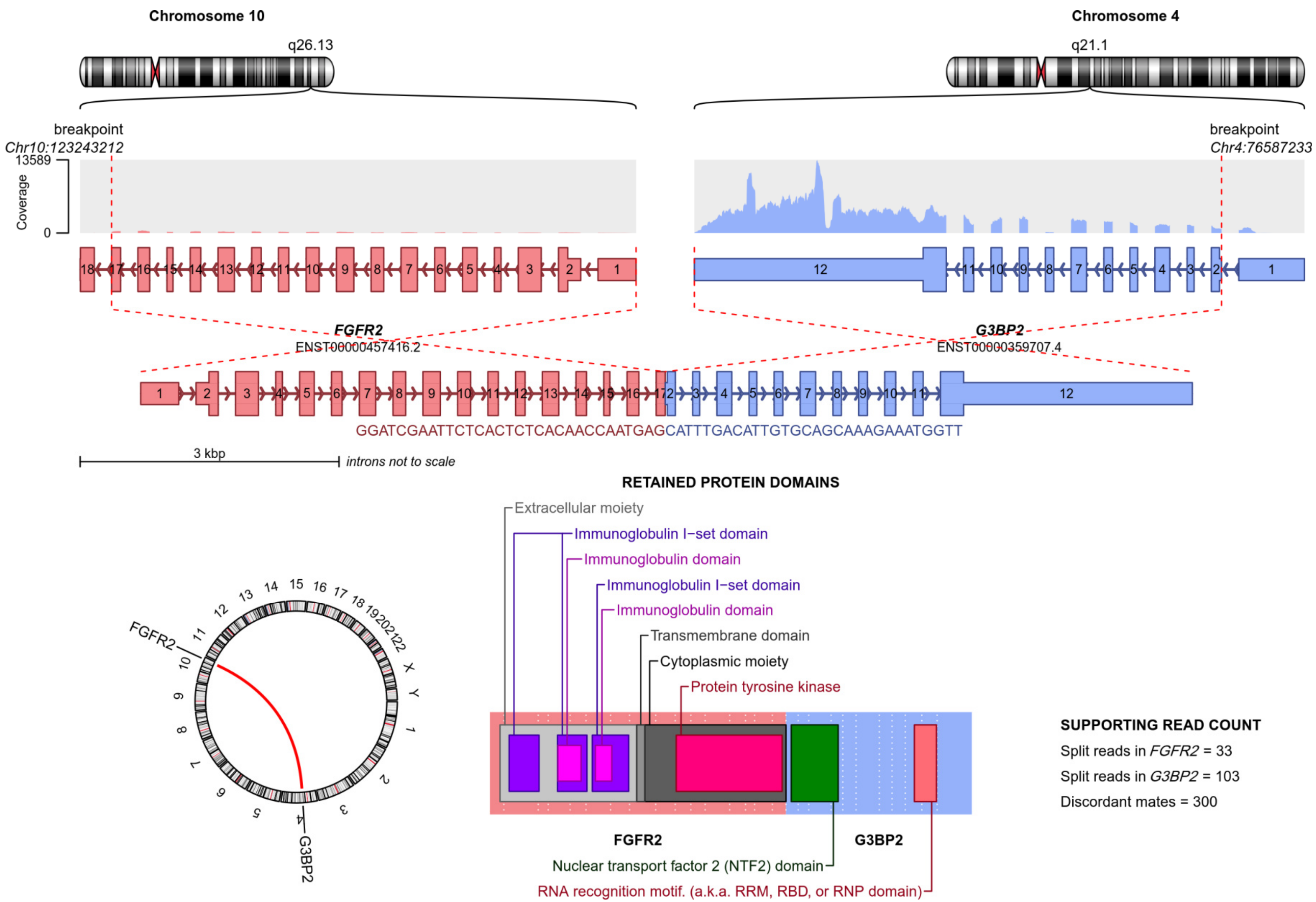

## ICGC_0338 (EGAF00001682709)

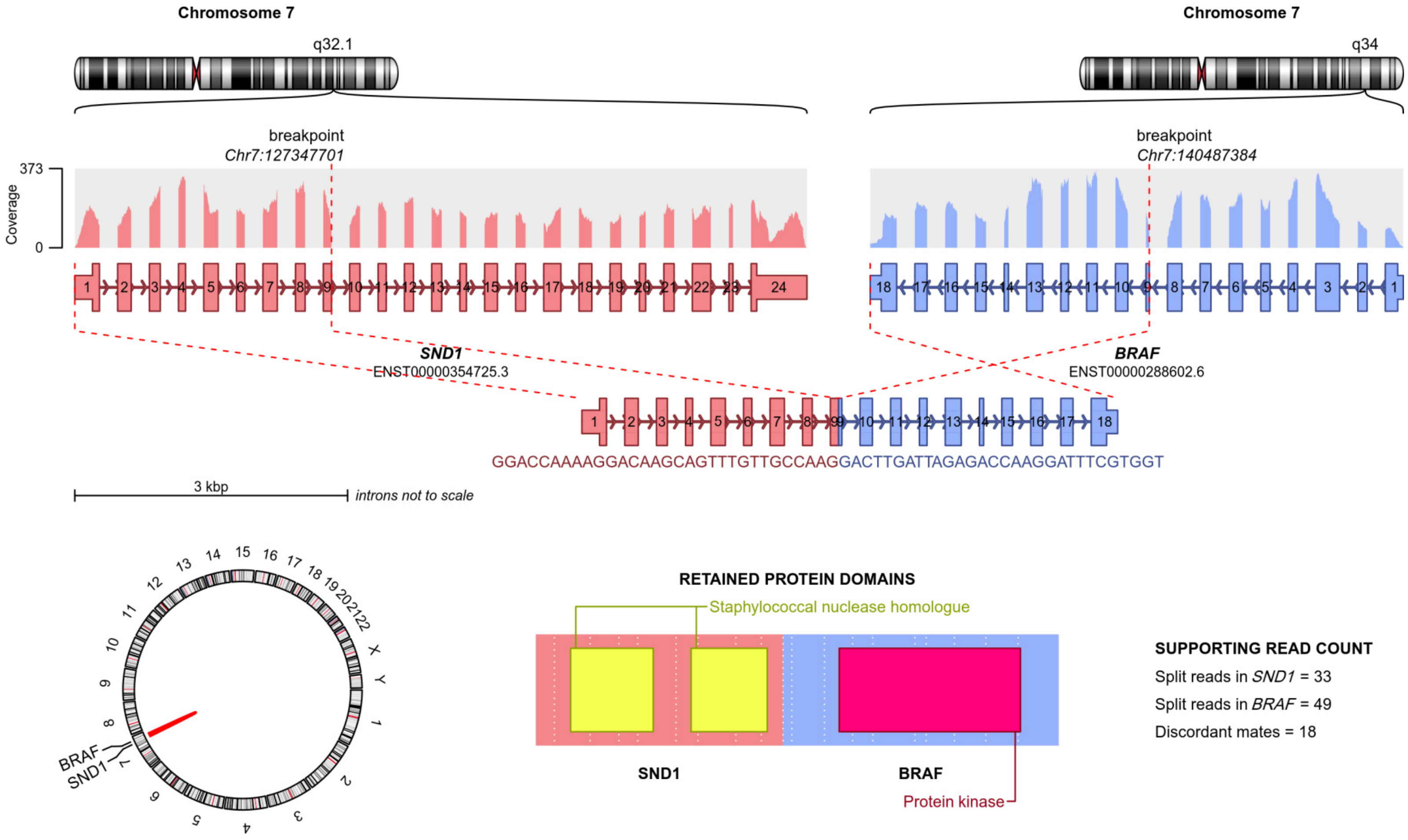

## ICGC_0391 (EGAF00001682868)

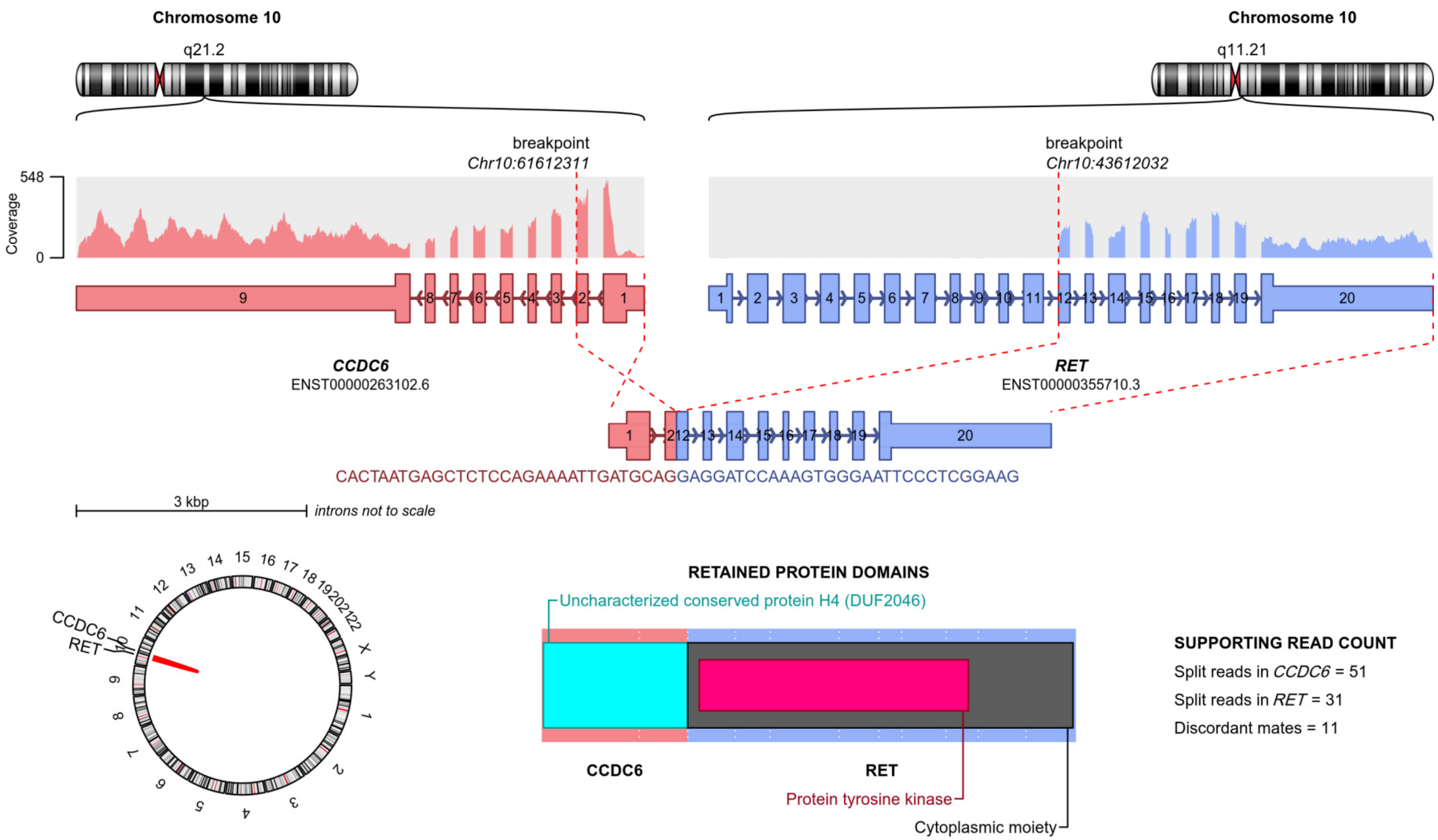

# PCSI_0572 (EGAF00001844274, EGAF00001879250, EGAF00001879251)

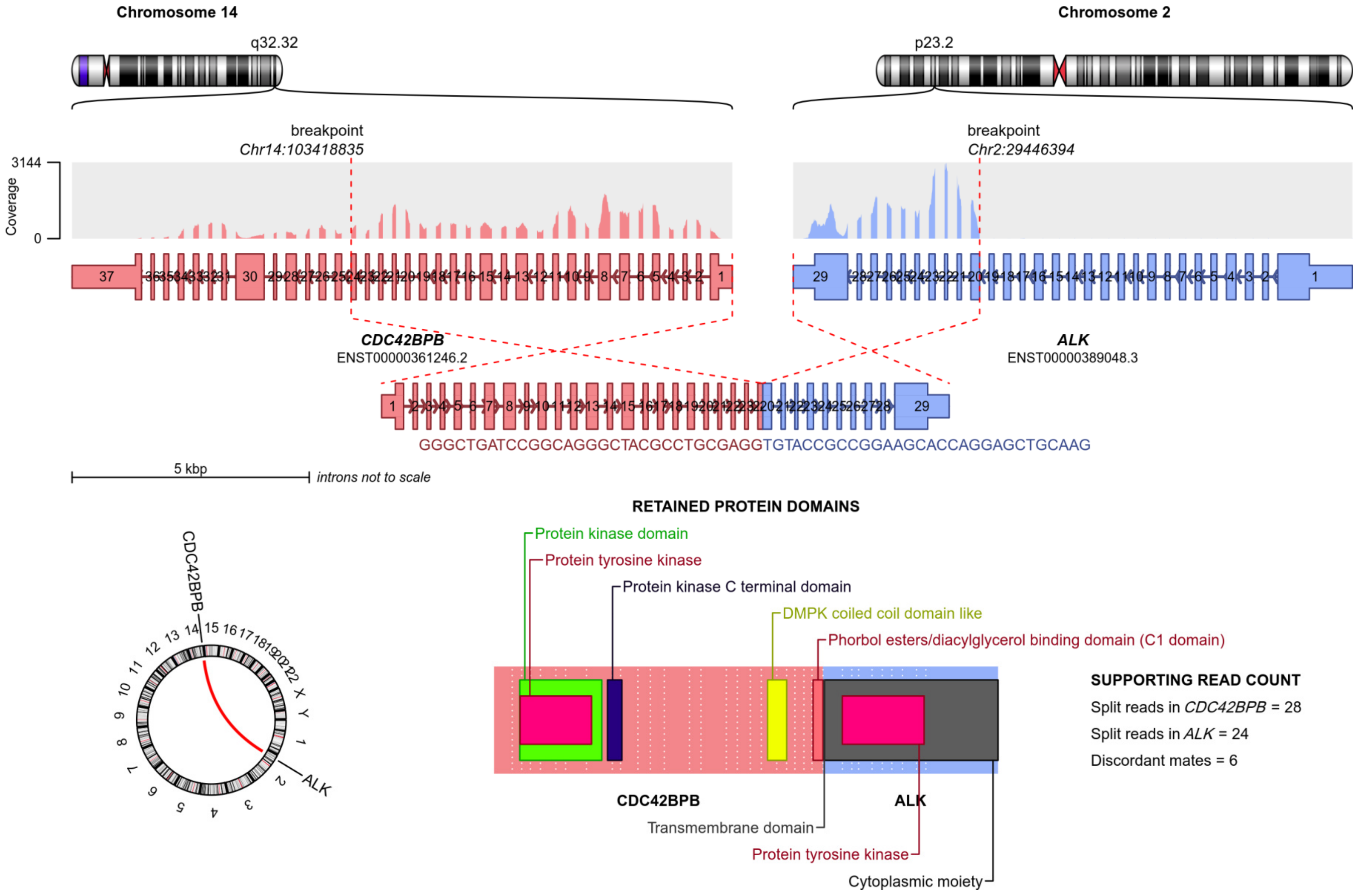

## PCSI_0108 (EGAF00001844288, EGAF00001878986, EGAF00001878987)

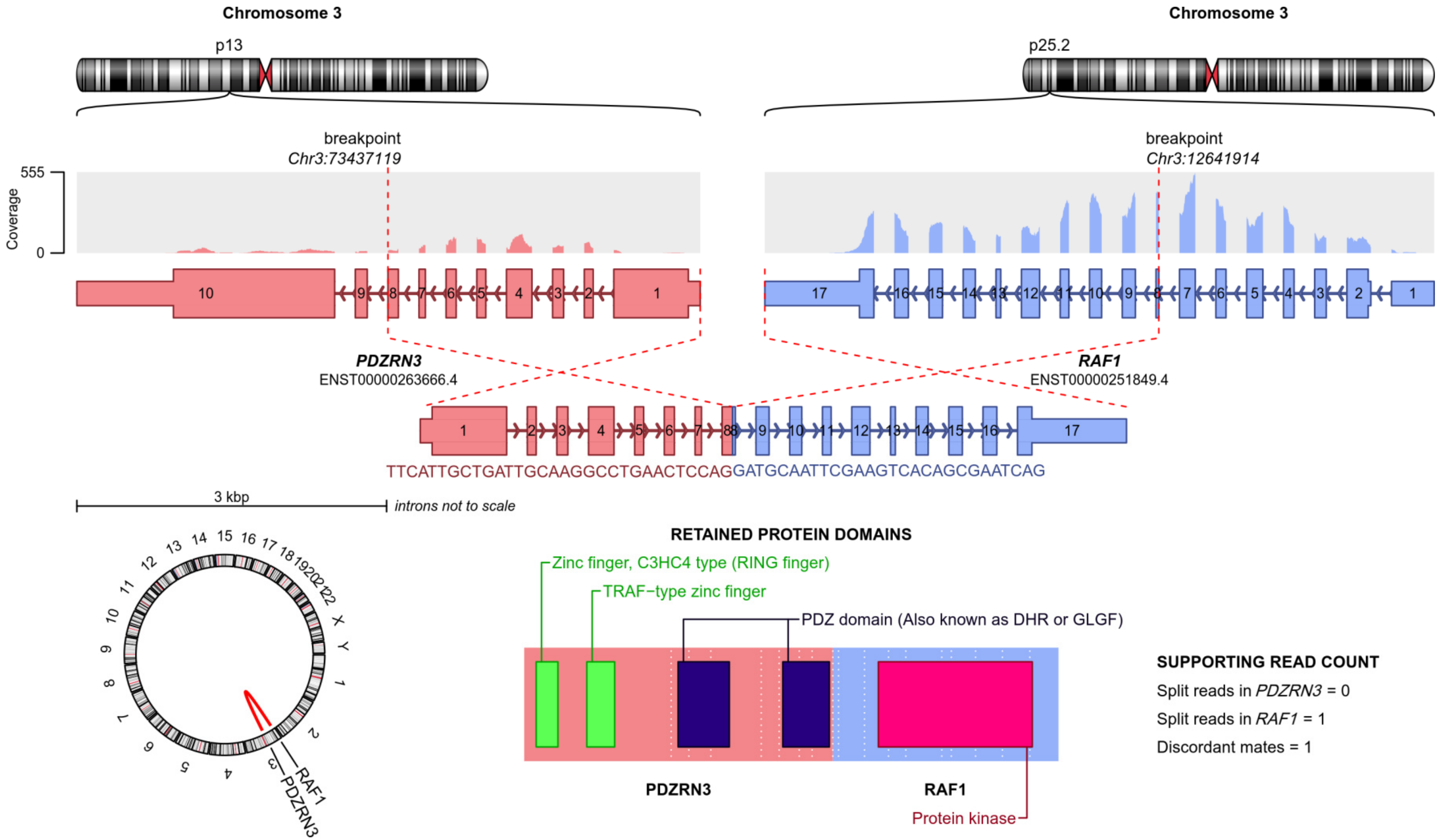

## PCSI_0279 (EGAF00001844342, EGAF00001879056, EGAF00001879057)

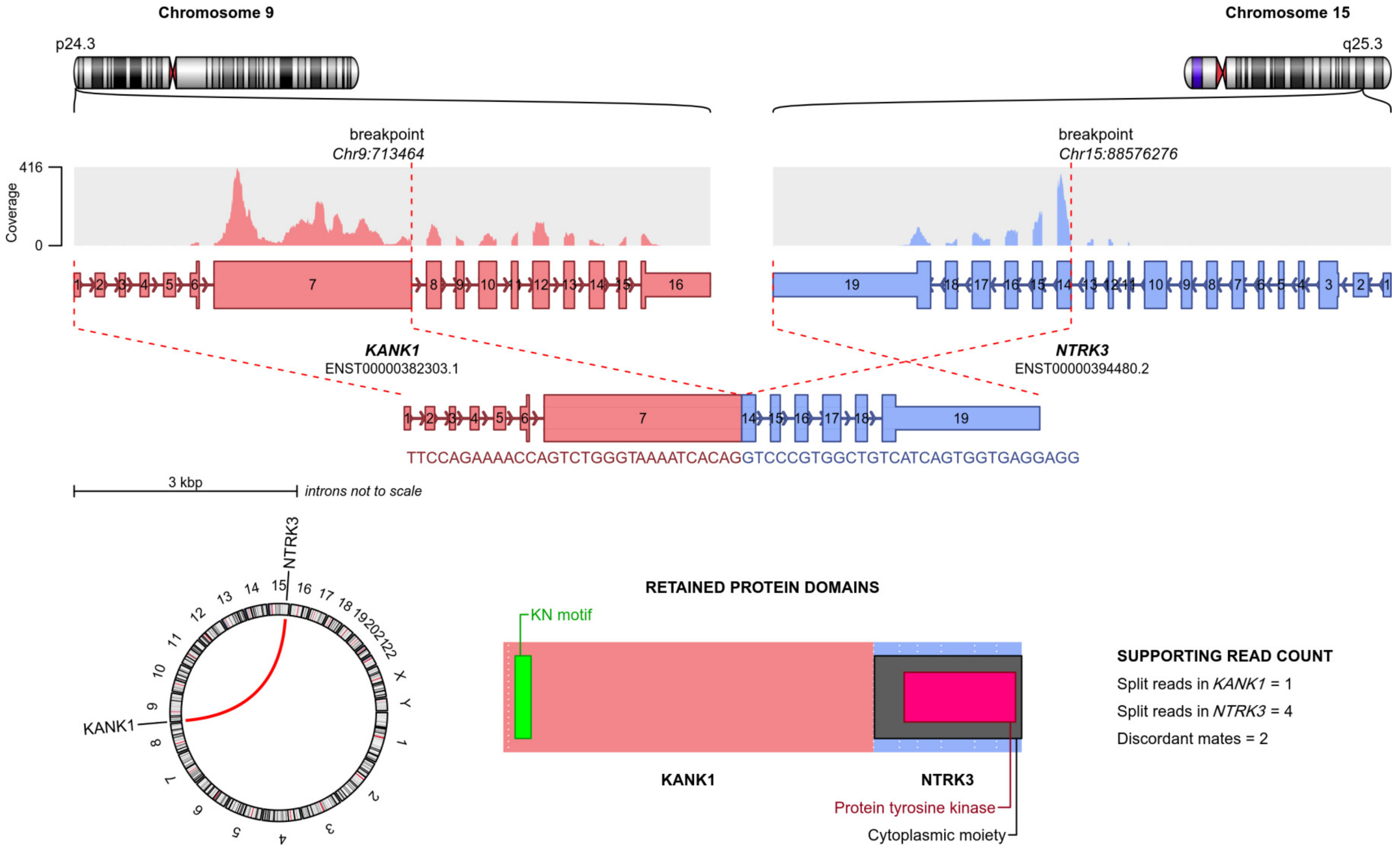

**PCSI_0305 (EGAF00001844367, EGAF00001879088, EGAF00001879089)**

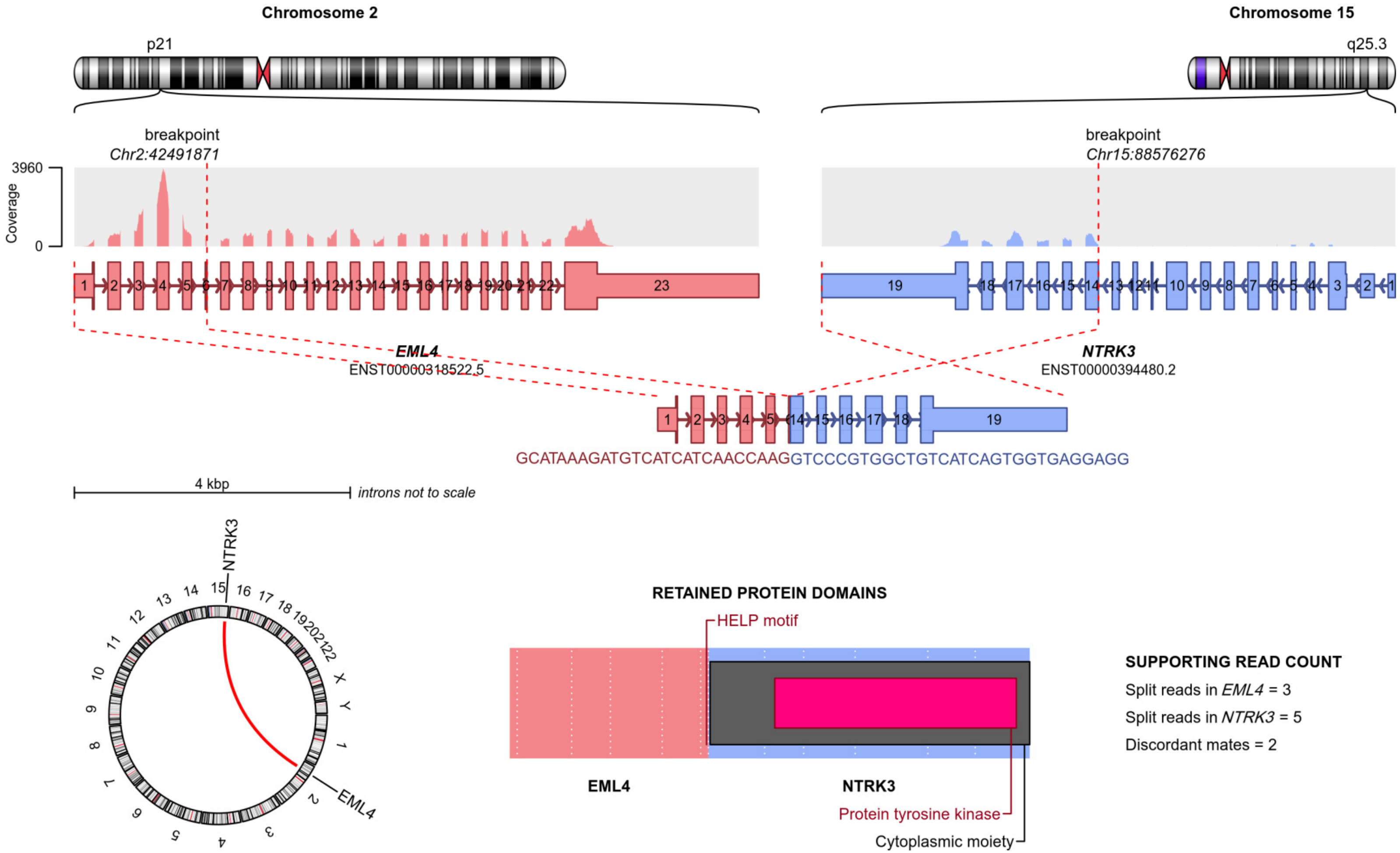

## PCSI_0326 (EGAF00001844548, EGAF00001879104, EGAF00001879105)

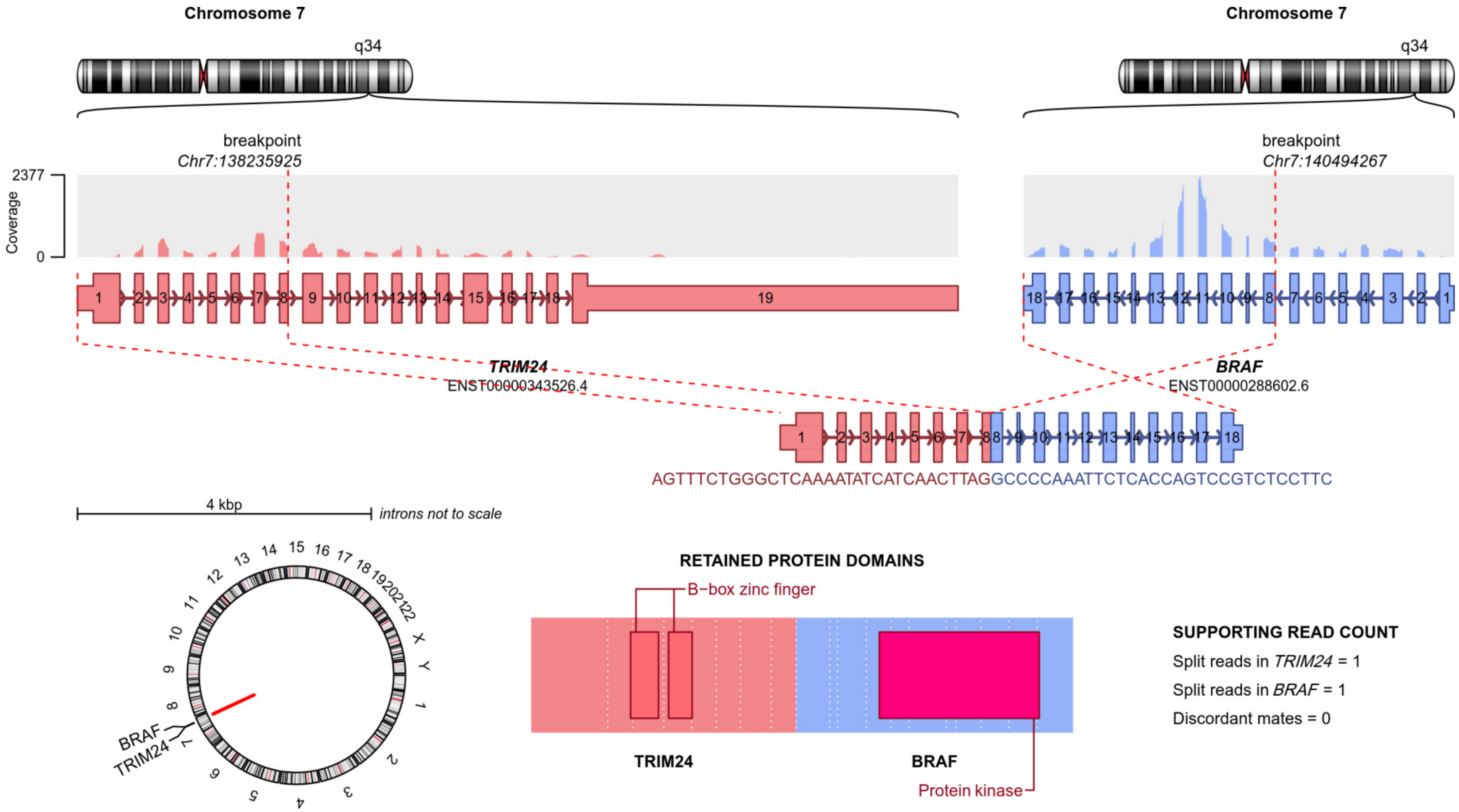

## PCSI_0330 (EGAF00001844551, EGAF00001879110, EGAF00001879111)

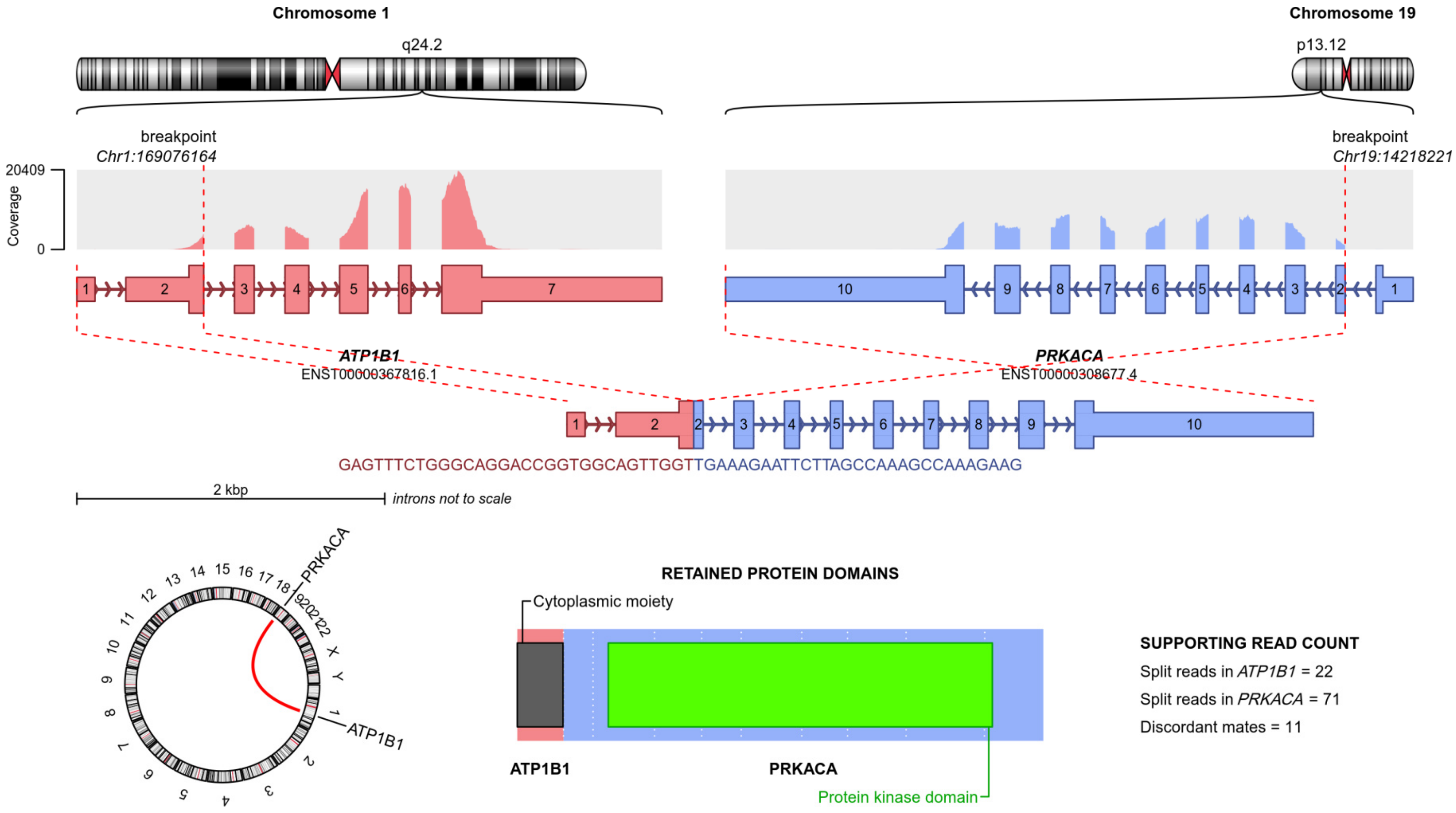

## PCSI_0347 (EGAF00001844561, EGAF00001879130, EGAF00001879131)

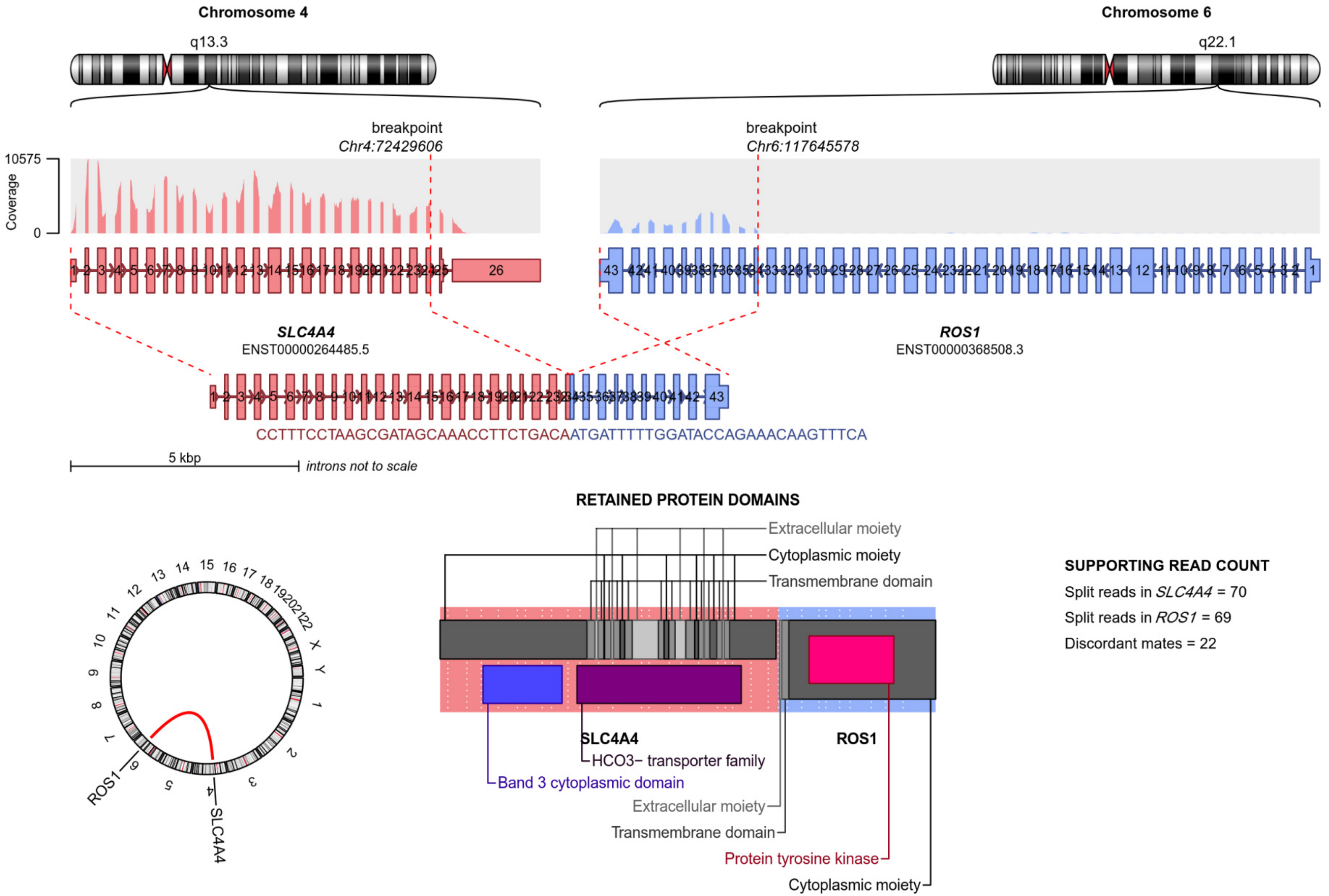

## PCSI_0458 (EGAF00001844592, EGAF00001879192, EGAF00001879193)

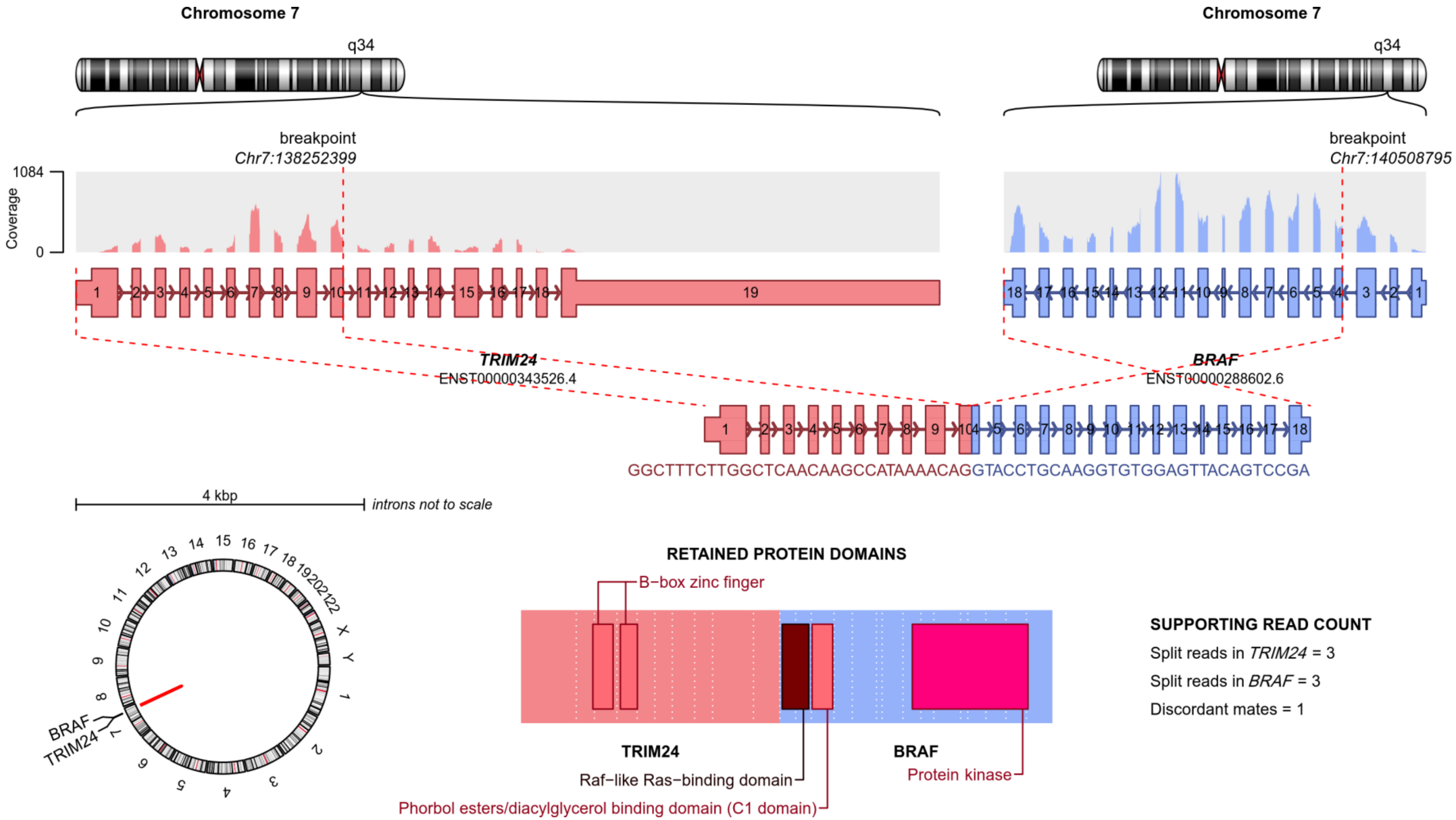

# TCGA-3A-A9I5 (163427ab-f1e7-4d63-91a0-66a0513ac795)

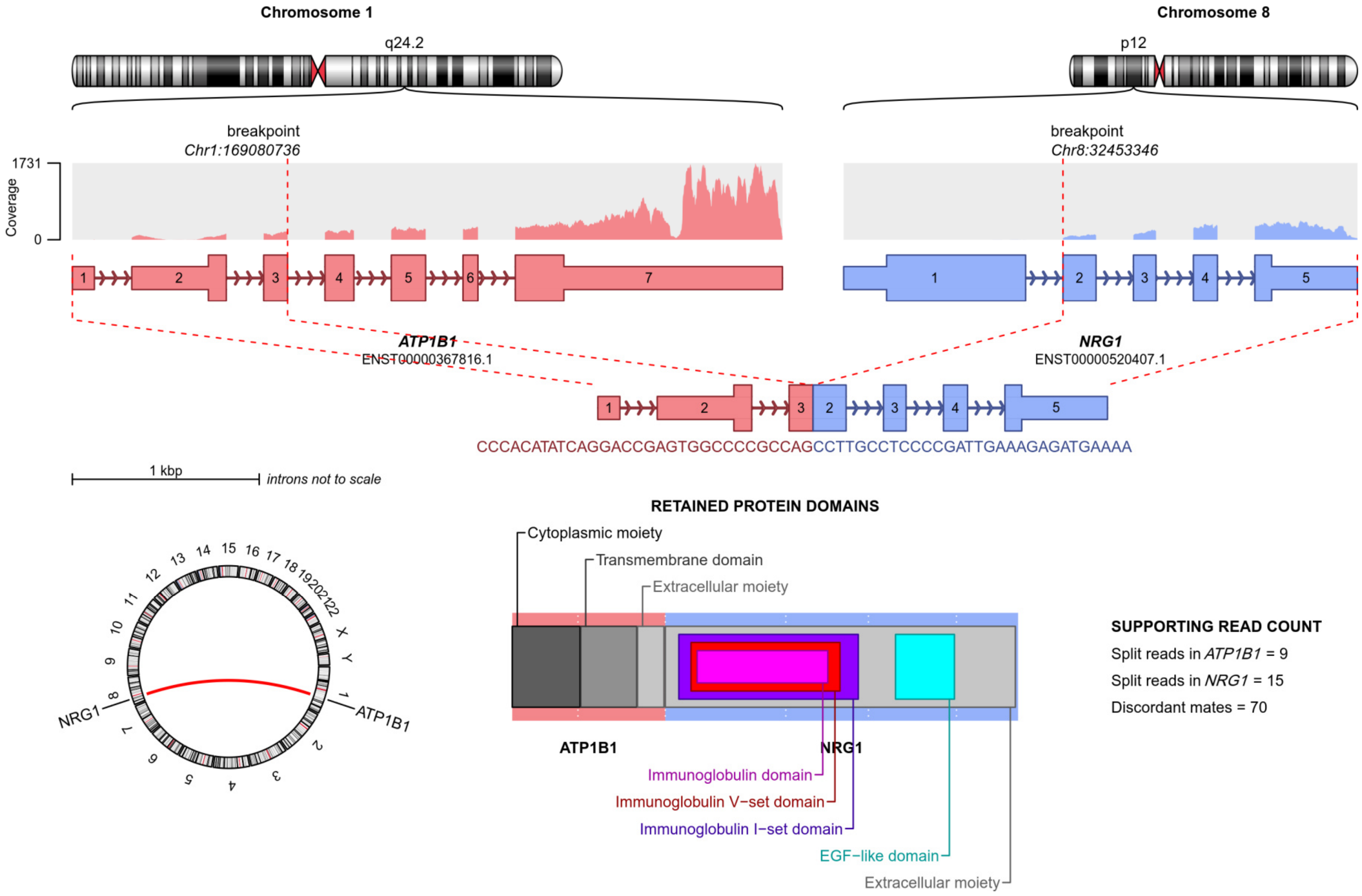


**SUPPORTING READ COUNT**

Split reads in *ATP1B1* = 9

Split reads in *NRG1* = 15

Discordant mates = 70

## TCGA-F2-A44H (65d99cd1-7601-4dec-a9dd-e6d28880164d)

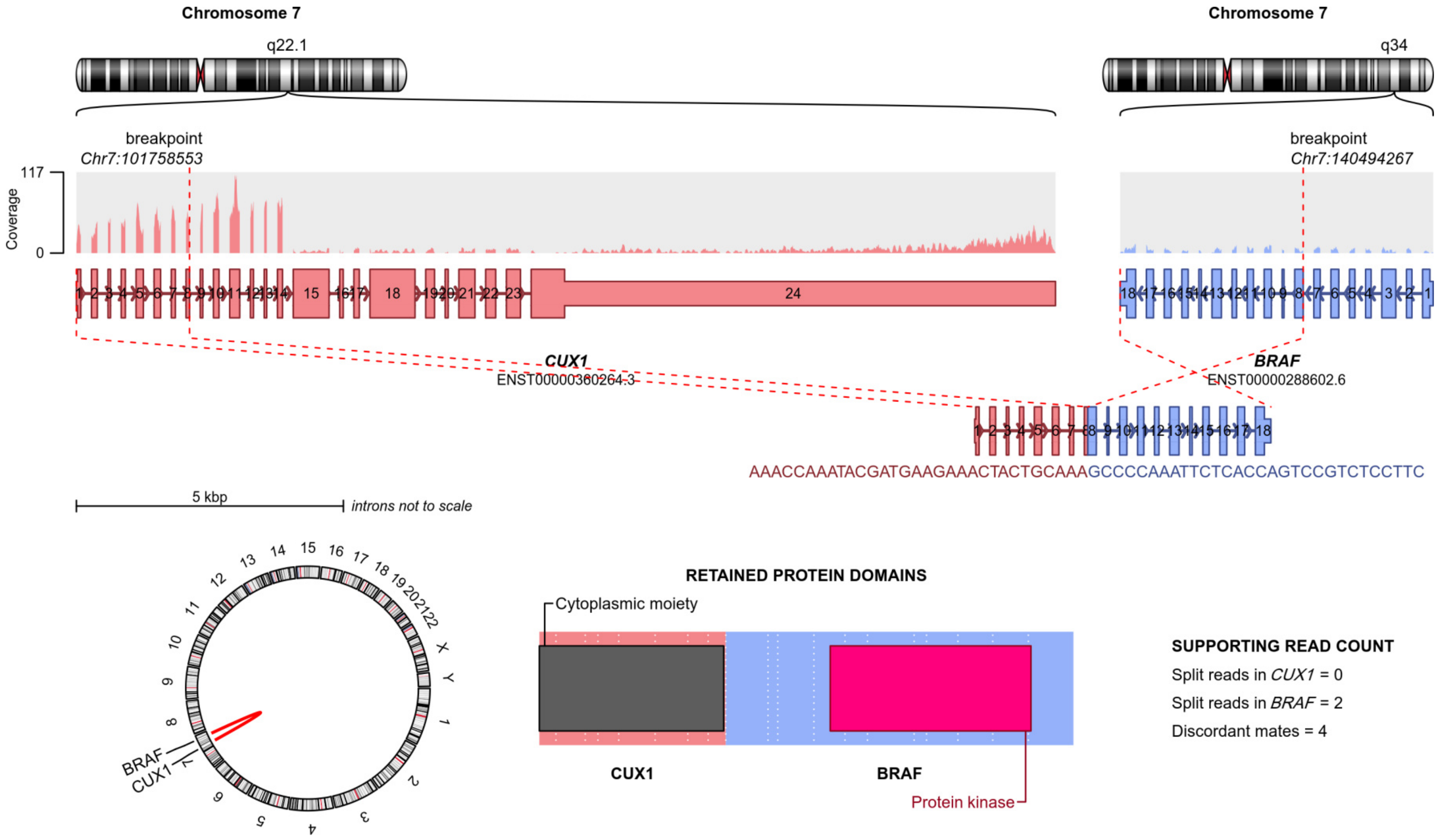

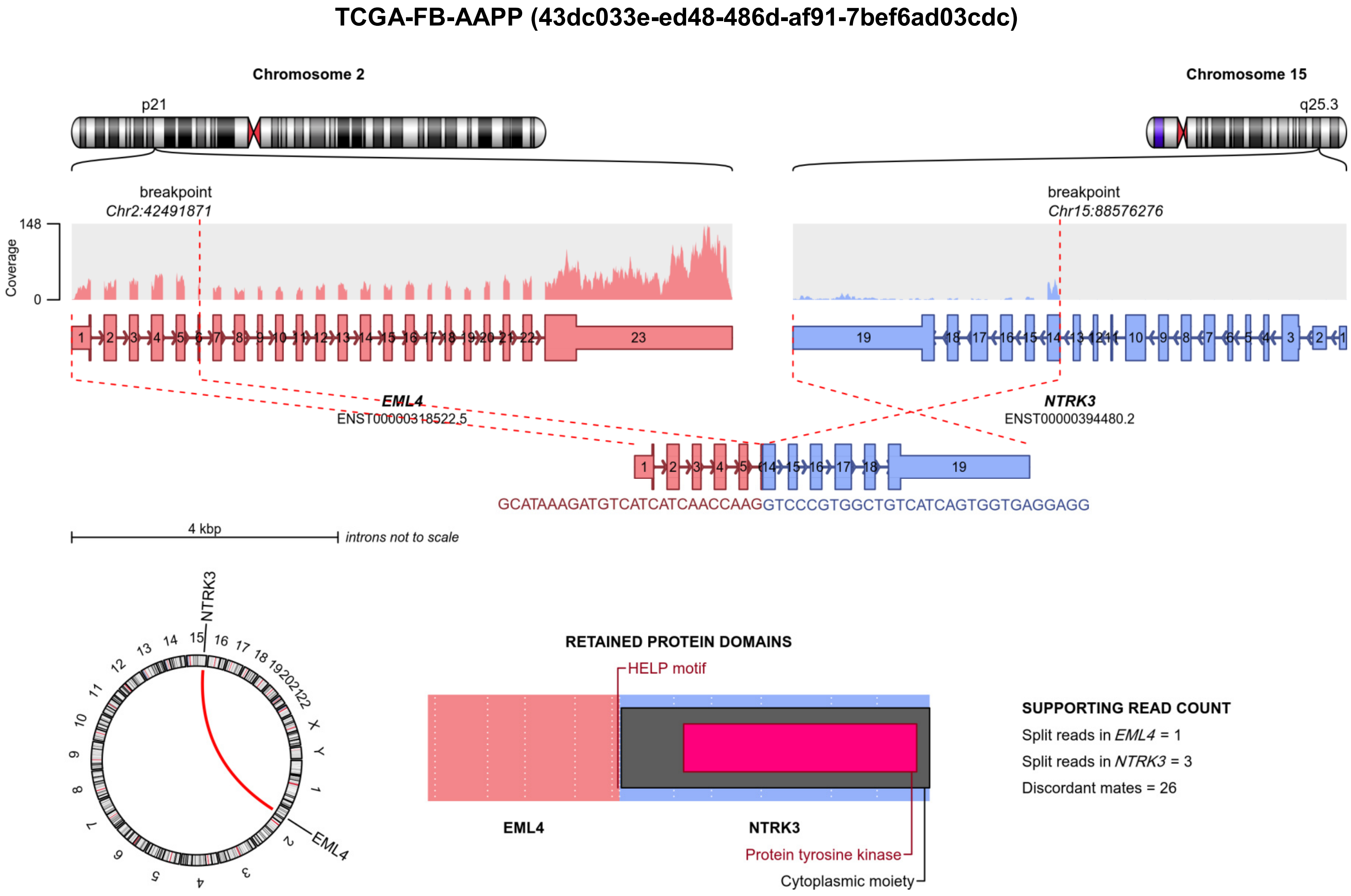
TCGA-FB-AAPP (43dc033e-ed48-486d-af91-7bef6ad03cdc)
Chromosome 2
p21
Chromosome 15
q25.3
breakpoint
Chr2:42491871
breakpoint
Chr15:88576276
Coverage
148
0
EML4
ENST00000318522.5
NTRK3
ENST00000394480.2
GCATAAAGATGTCATCATCAACCAAGGTCCCGTGGCTGTCATCAGTGGTGAGGAGG
4 kbp
introns not to scale
NTRK3
EML4
RETAINED PROTEIN DOMAINS
HELP motif
EML4
NTRK3
Protein tyrosine kinase
Cytoplasmic moiety
SUPPORTING READ COUNT
Split reads in EML4 = 1
Split reads in NTRK3 = 3
Discordant mates = 26

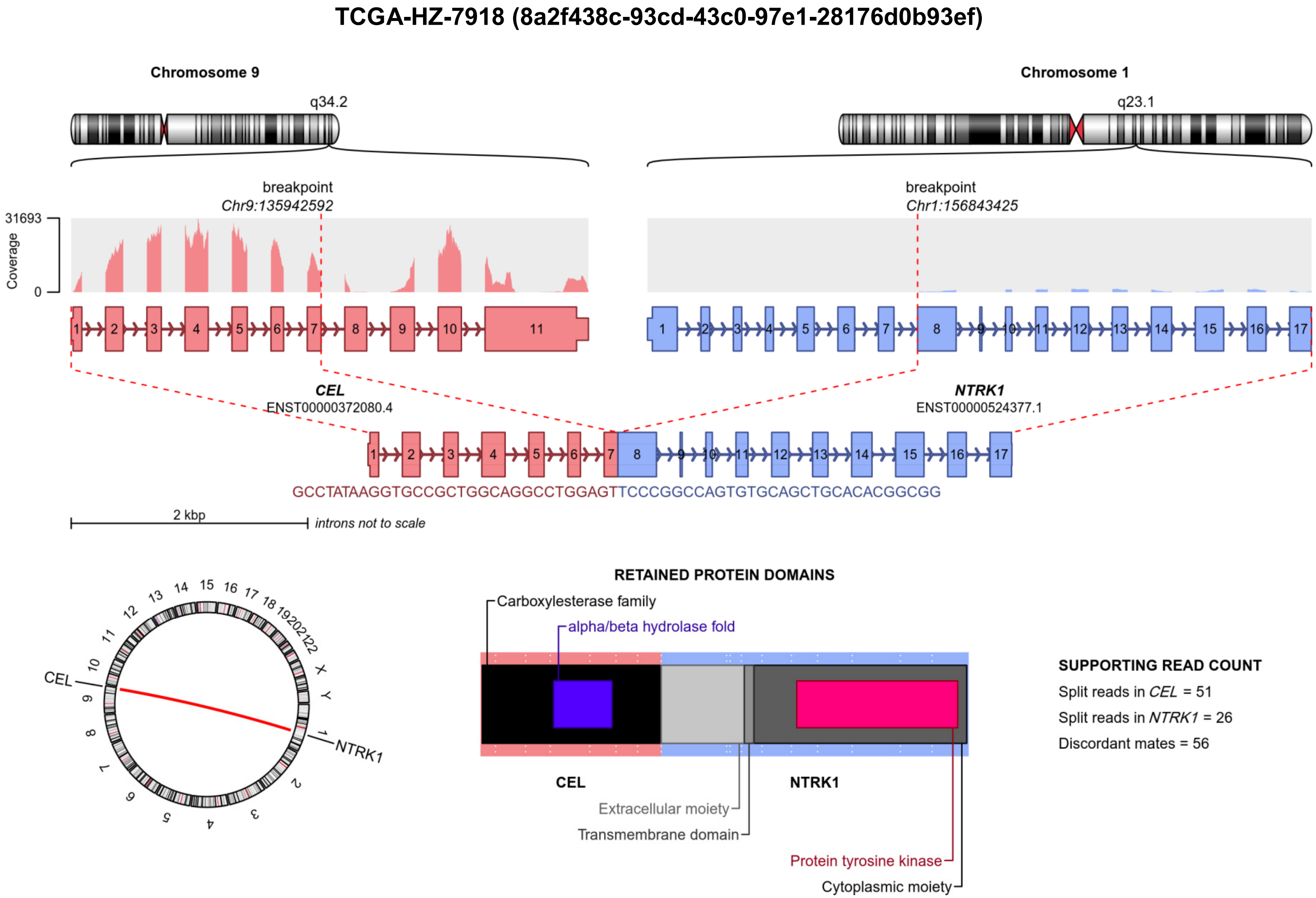


**Supplemental Figure S6: Putative driver fusions identified by Arriba in pancreatic cancer samples.**
In 30 of 803 RNA-Seq samples from pancreatic cancer patients Arriba identified a potential oncogenic fusion.
