# Supplementary material for: Accurate and efficient detection of gene fusions from RNA sequencing data
Source: Genome Res. 2021 Mar;31(3):448–60. doi: 10.1101/gr.257246.119 (PMC7919457; doi:10.1101/gr.257246.119)
Supplement: Supplemental Material [file supp_gr.257246.119_Supplemental_Figure_S7.pdf]

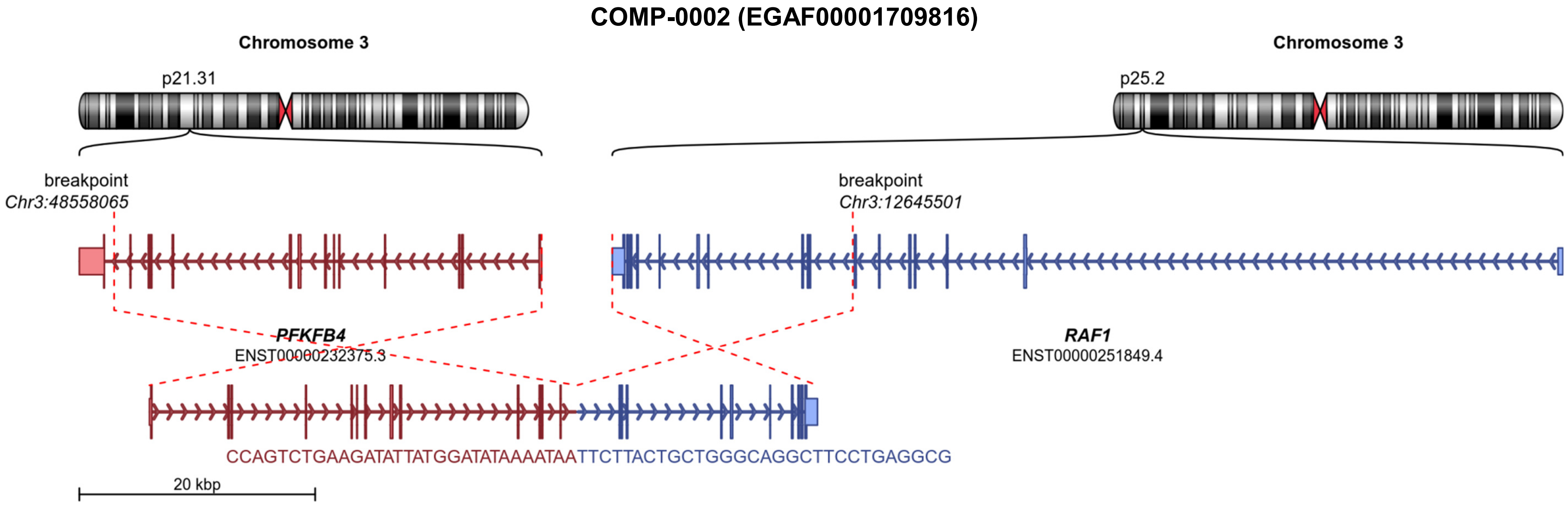
COMP-0002 (EGAF00001709816)
Chromosome 3
p21.31
Chromosome 3
p25.2
breakpoint
Chr3:48558065
breakpoint
Chr3:12645501
PFKFB4
ENST00000232375.3
RAF1
ENST00000251849.4
CCAGTCTGAAGATATTATGGATATAAAATAATTCTTACTGCTGGGCAGGCTTCCTGAGGCG
20 kbp

COMP-0023 (EGAF00001709871)

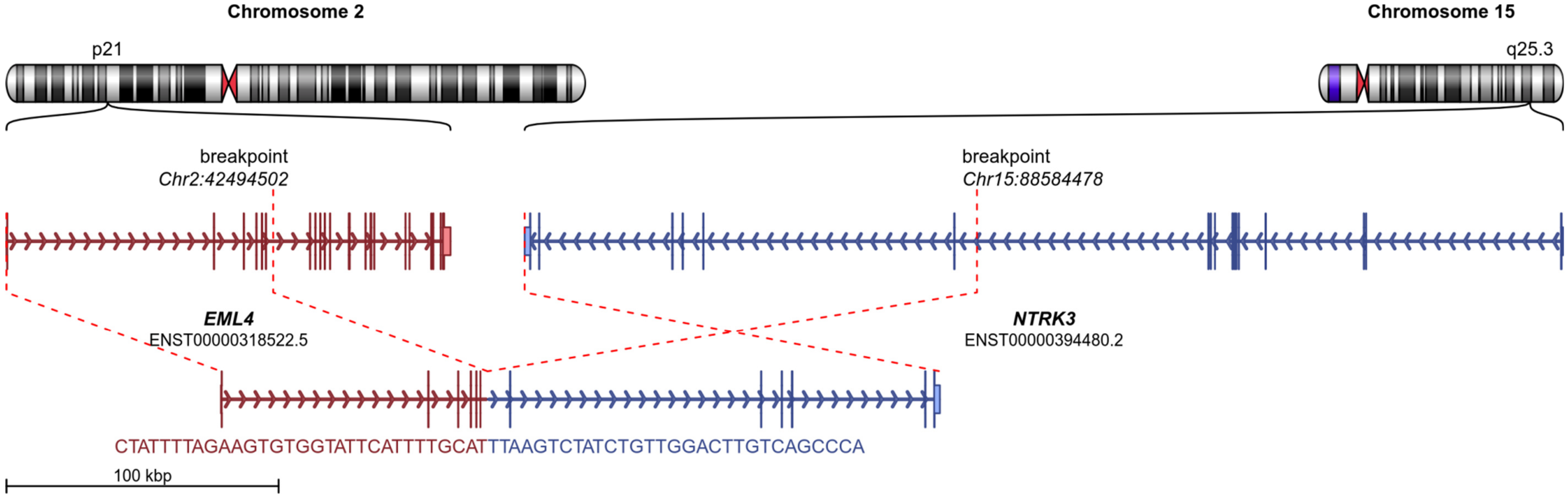

COMP-0028 (EGAF00001709880)

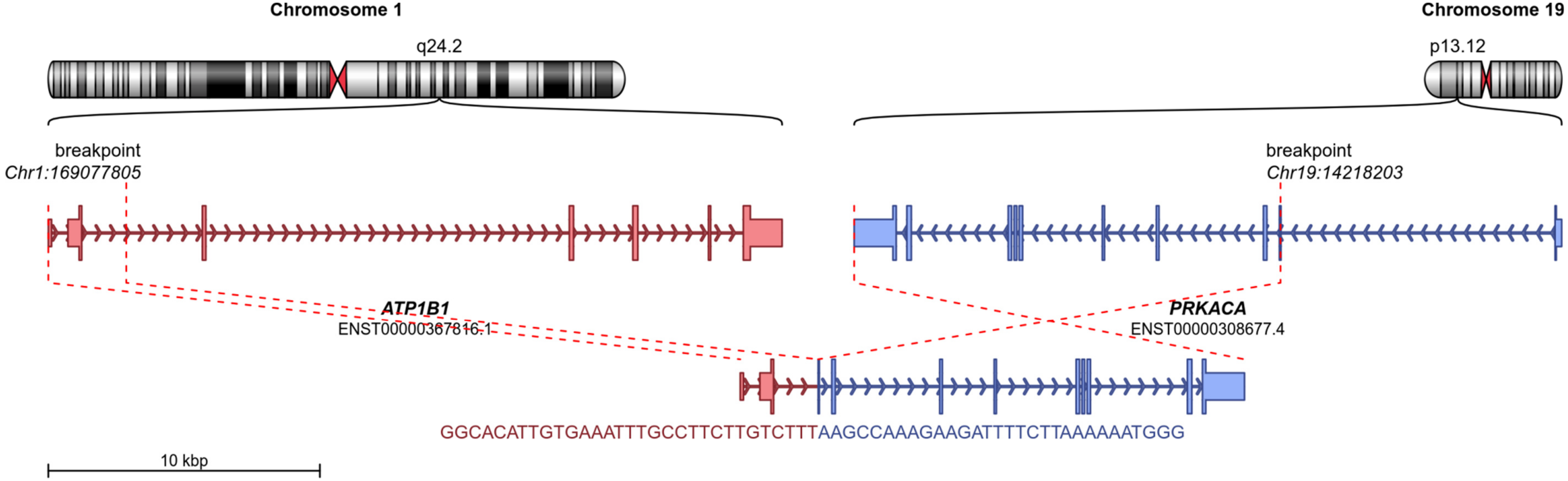

COMP-0055 (EGAF00001720843)

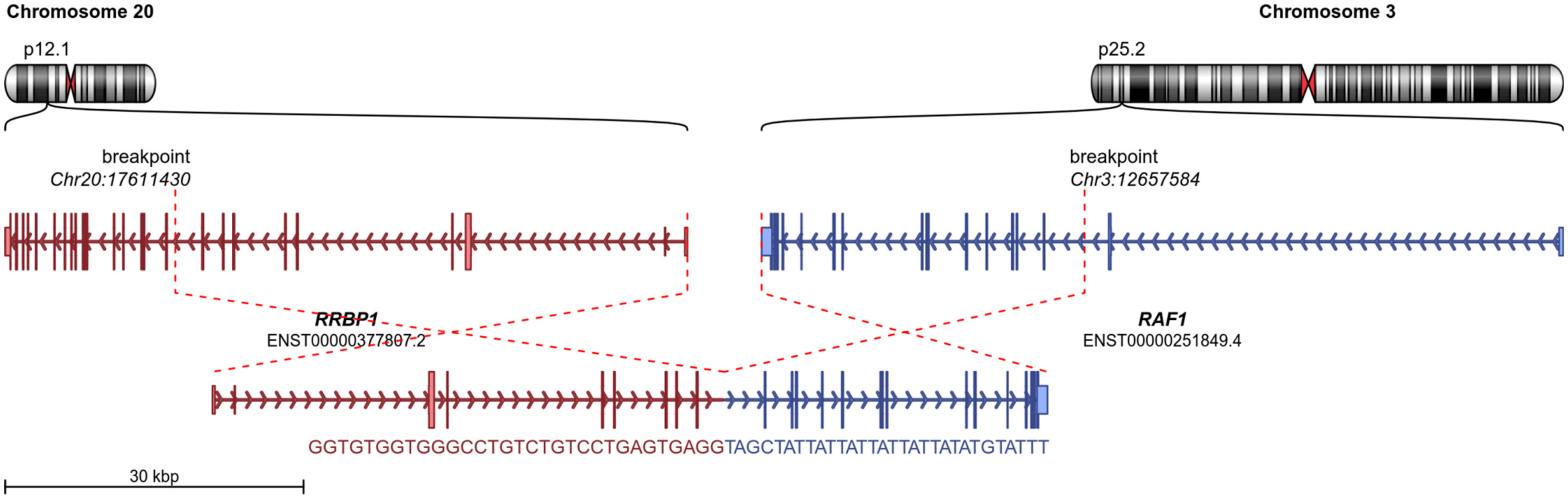

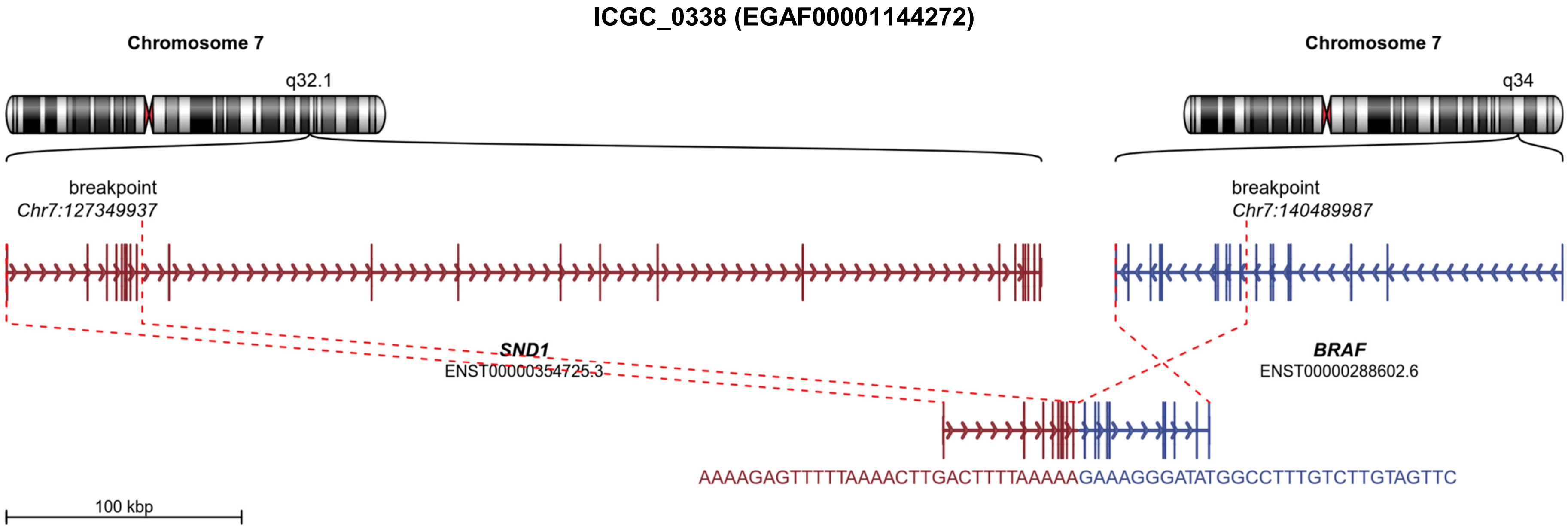
ICGC_0338 (EGAF00001144272)
Chromosome 7
q32.1
Chromosome 7
q34
breakpoint
Chr7:127349937
breakpoint
Chr7:140489987
SND1
ENST00000354725.3
BRAF
ENST00000288602.6
AAAAGAGTTTTTAAAACTTGACTTTTAAAAAGAAAGGGATATGGCCTTTGTCTTGTAGTTC
100 kbp

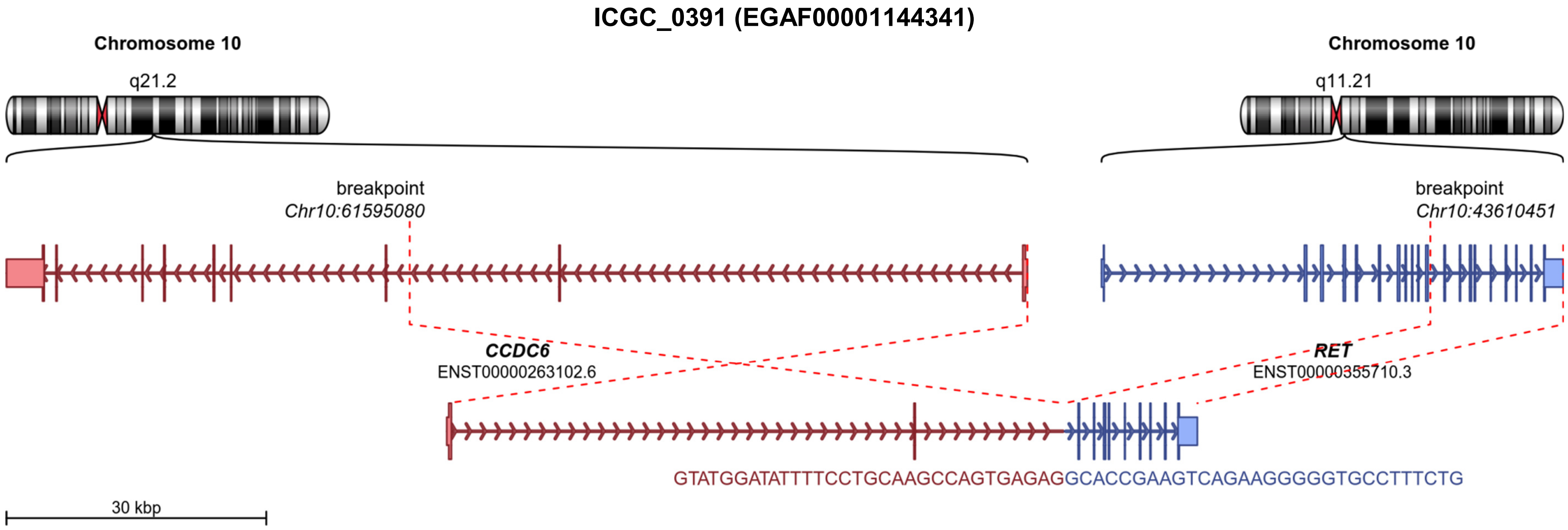
ICGC_0391 (EGAF00001144341)
Chromosome 10
q21.2
Chromosome 10
q11.21
breakpoint
Chr10:61595080
breakpoint
Chr10:43610451
CCDC6
ENST00000263102.6
RET
ENST00000355710.3
GTATGGATATTTTCCTGCAAGCCAGTGAGAGGCACCGAAGTCAGAAGGGGGTGCCTTTCTG
30 kbp

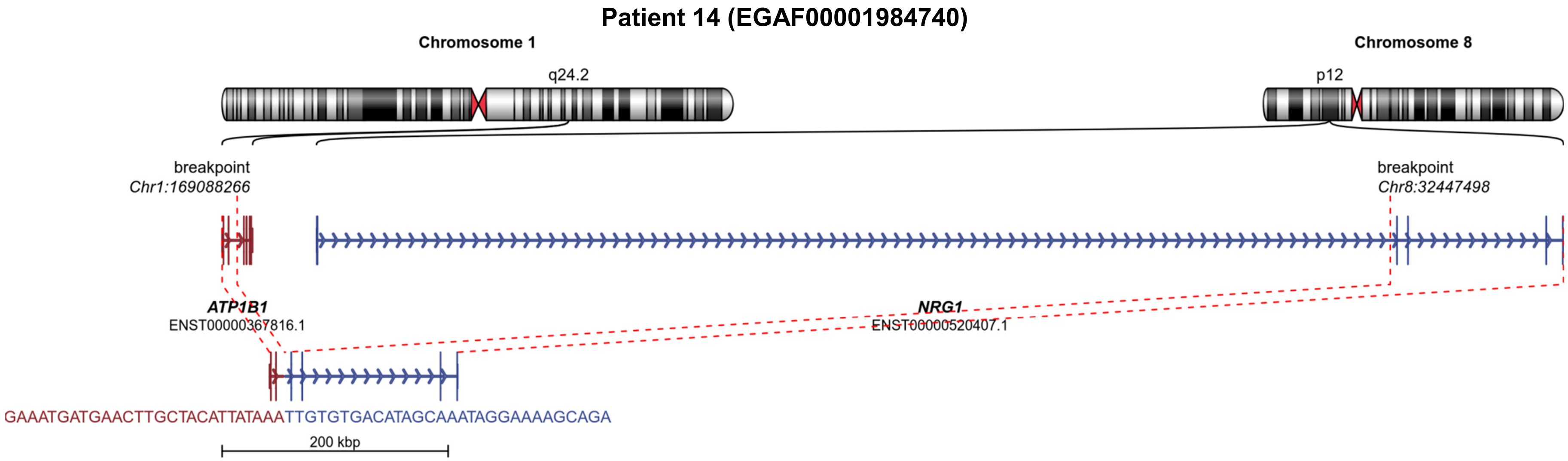

Patient 14 (EGAF00001984740)
Chromosome 1
Chromosome 8
q24.2
p12
breakpoint
Chr1:169088266
breakpoint
Chr8:32447498
ATP1B1
ENST00000367816.1
NRG1
ENST00000520407.1
GAAATGATGAACTTGCTACATTATAAATTGTGTGACATAGCAAATAGGAAAAGCAGA
200 kbp

Patient 17 (EGAF00001984732)

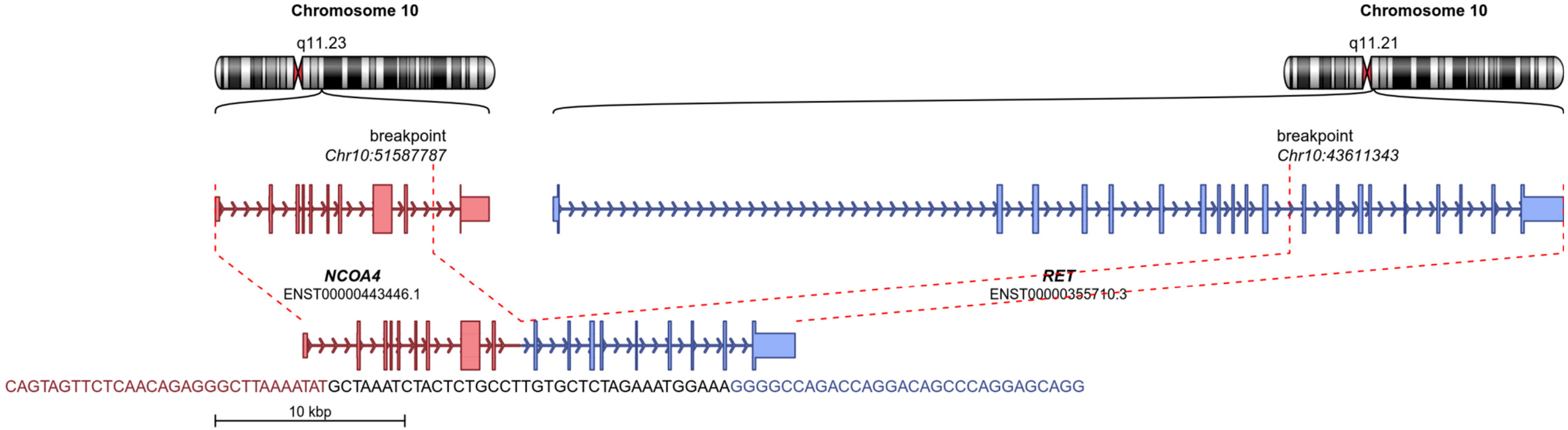

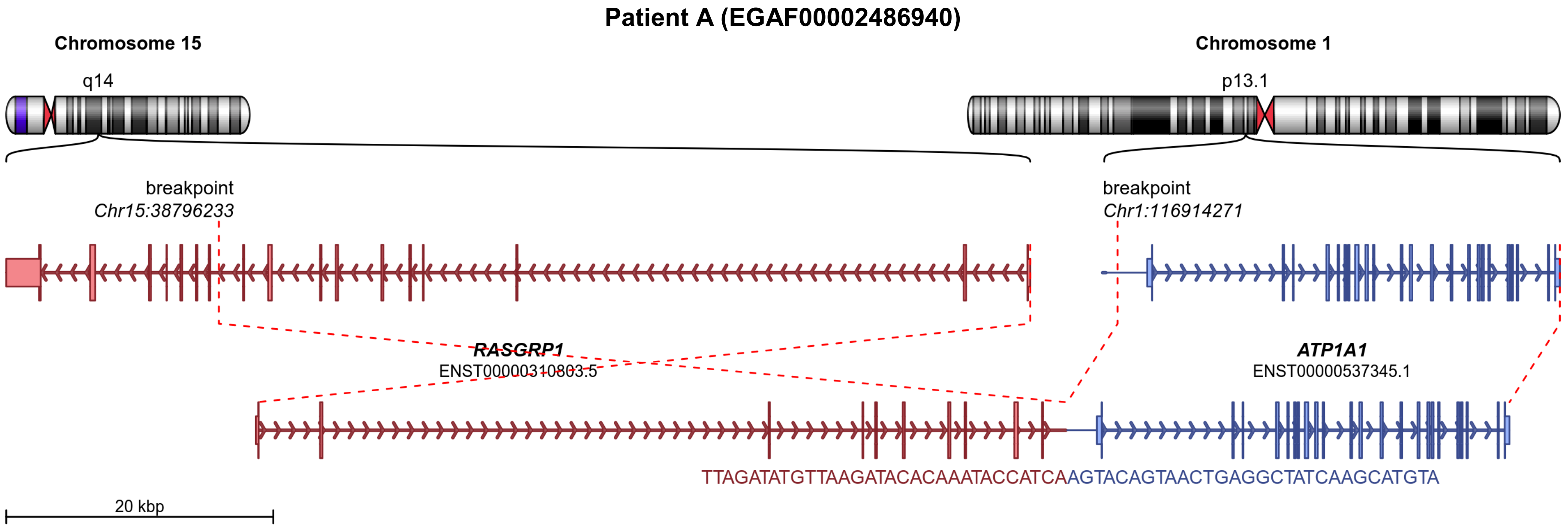

Patient A (EGAF00002486940)
Chromosome 15
Chromosome 1
q14
p13.1
breakpoint
Chr15:38796233
breakpoint
Chr1:116914271
RASGRP1
ENST00000310803.5
ATP1A1
ENST00000537345.1
TTAGATATGTTAAGATACACAAATACCATCAAGTACAGTAACTGAGGCTATCAAGCATGTA
20 kbp

PCSI_0108 (EGAF00001556794)

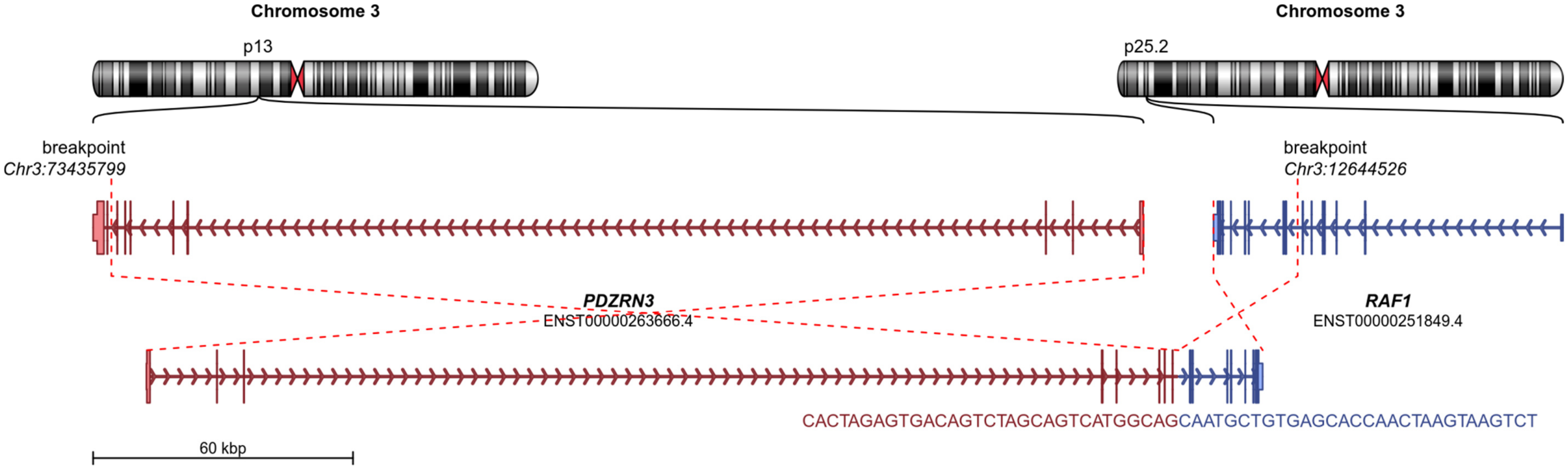

PCSI_0279 (EGAF00001556830)

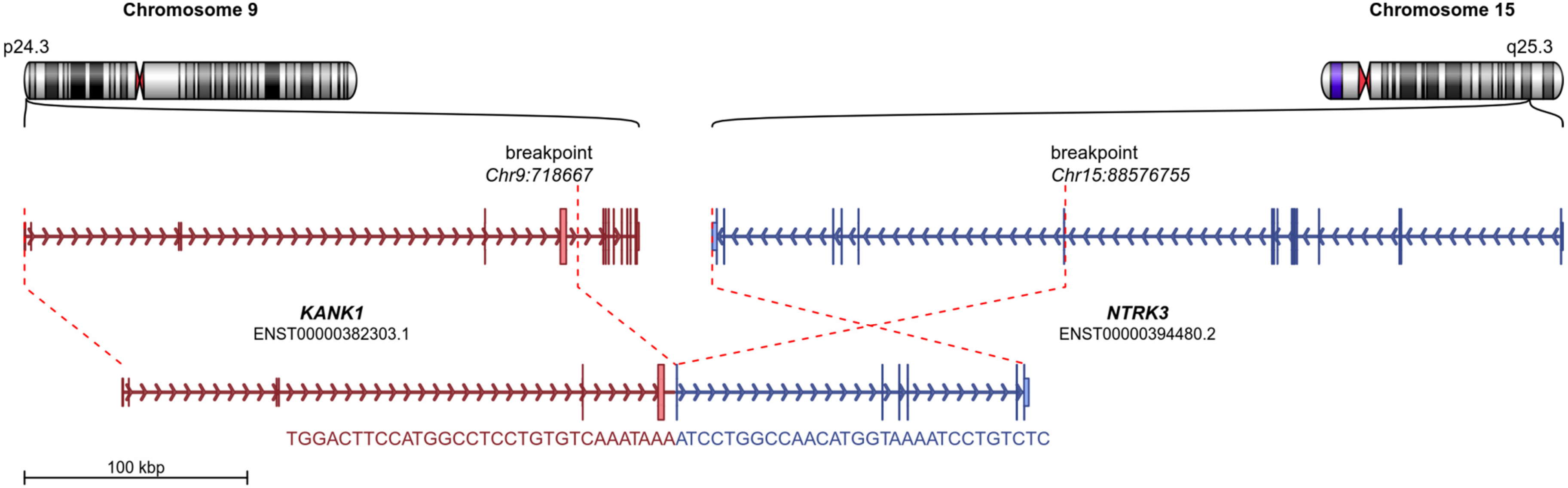

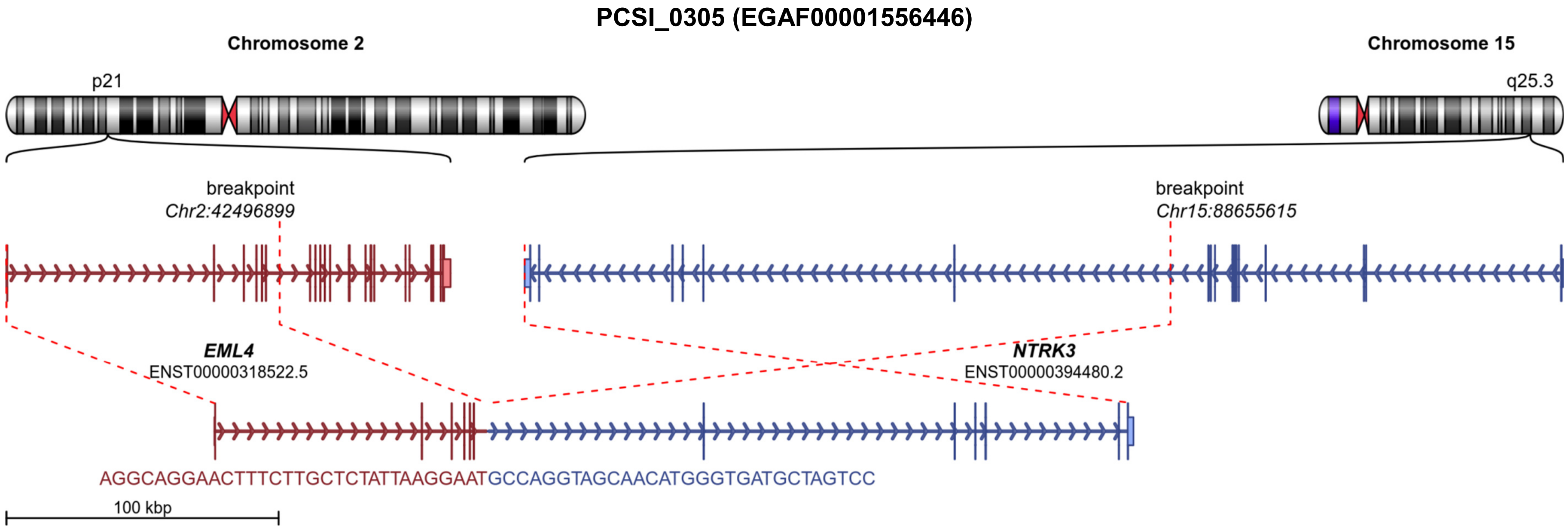
PCSI_0305 (EGAF00001556446)
Chromosome 2
Chromosome 15
p21
q25.3
breakpoint
Chr2:42496899
breakpoint
Chr15:88655615
EML4
ENST00000318522.5
NTRK3
ENST00000394480.2
AGGCAGGAACTTTCTTGCTCTATTAAGGAATGCCAGGTAGCAACATGGGTGATGCTAGTCC
100 kbp

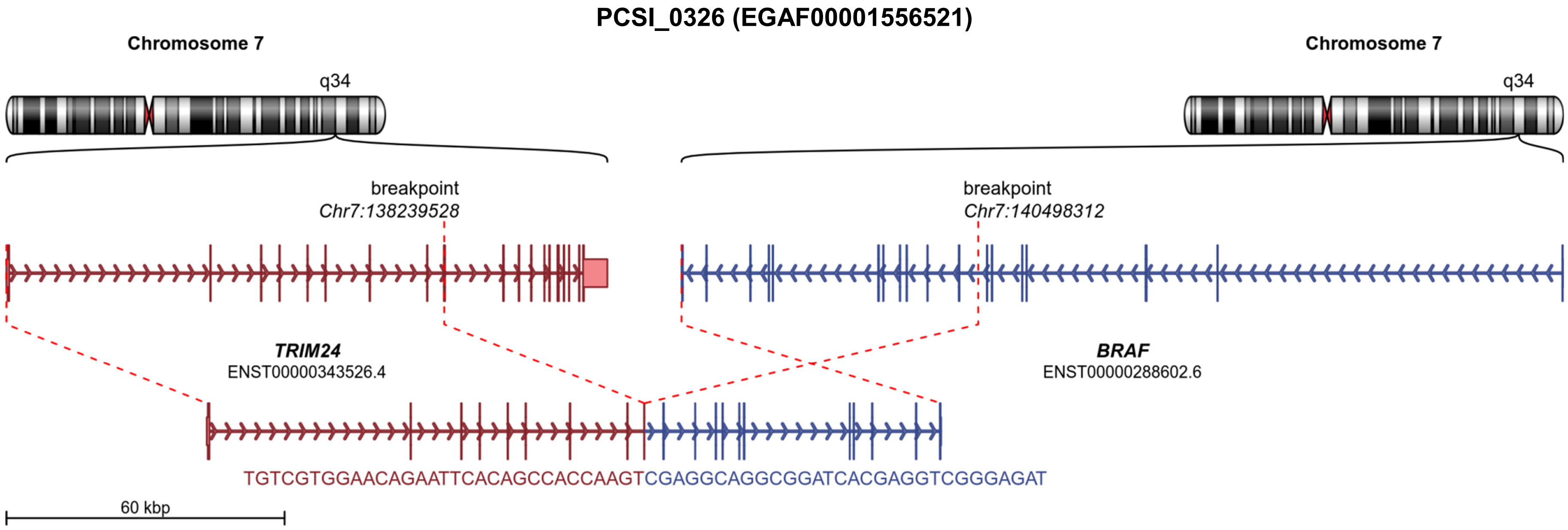
PCSI_0326 (EGAF00001556521)
Chromosome 7
q34
Chromosome 7
q34
breakpoint
Chr7:138239528
breakpoint
Chr7:140498312
TRIM24
ENST00000343526.4
BRAF
ENST00000288602.6
TGTCGTGGAACAGAATTCACAGCCACCAAGTCGAGGCAGGCGGATCACGAGGTCGGGAGAT
60 kbp

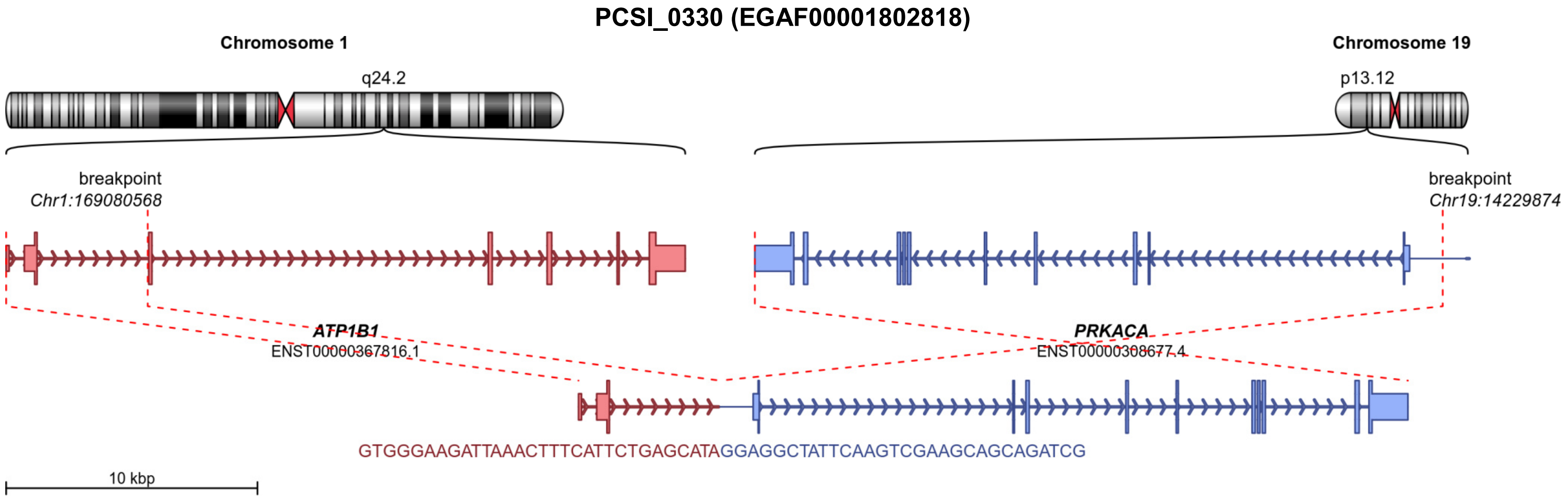

PCSI_0330 (EGAF00001802818)
Chromosome 1
q24.2
Chromosome 19
p13.12
breakpoint
Chr1:169080568
breakpoint
Chr19:14229874
ATP1B1
ENST00000367816.1
PRKACA
ENST00000308677.4
GTGGGAAGATTAAACTTTCATTCTGAGCATAGGAGGCTATTCAAGTCGAAGCAGCAGATCG
10 kbp

PCSI_0347 (EGAF00001556815)

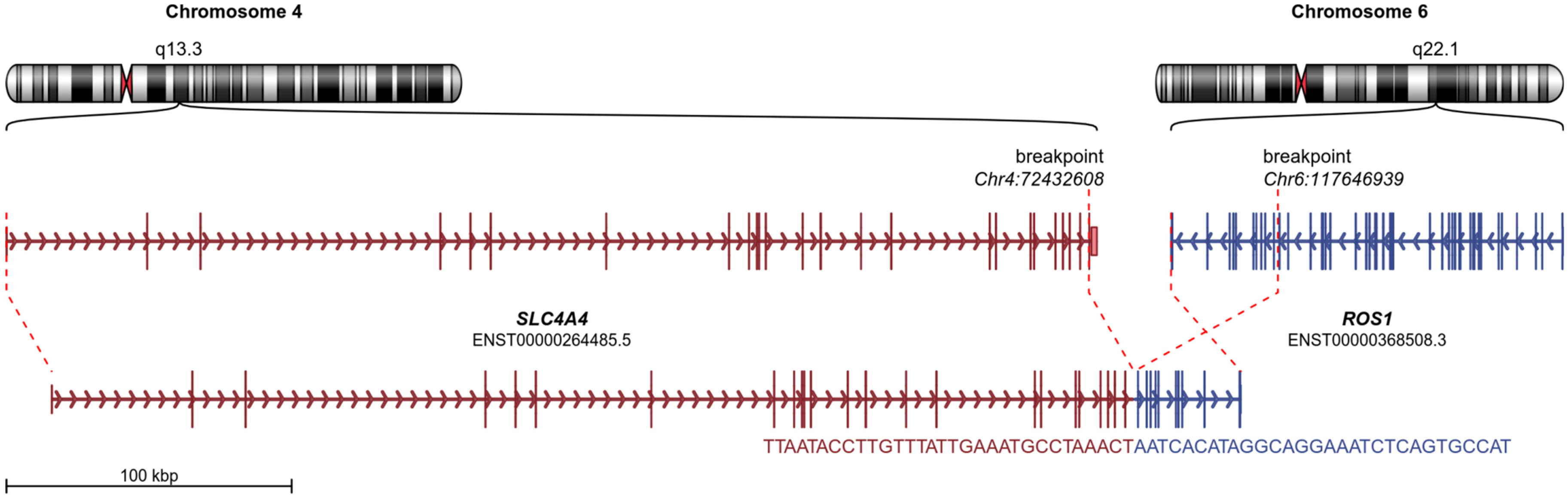

PCSI_0458 (EGAF00001556509)

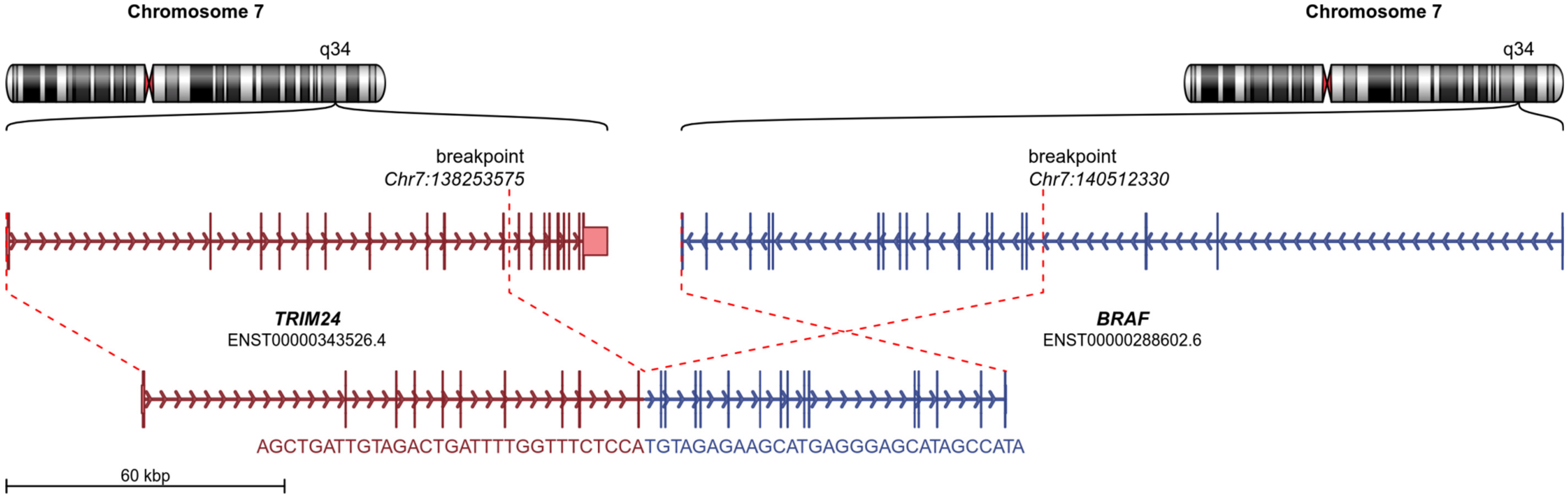

PCSI_0572 (EGAF00001556614)

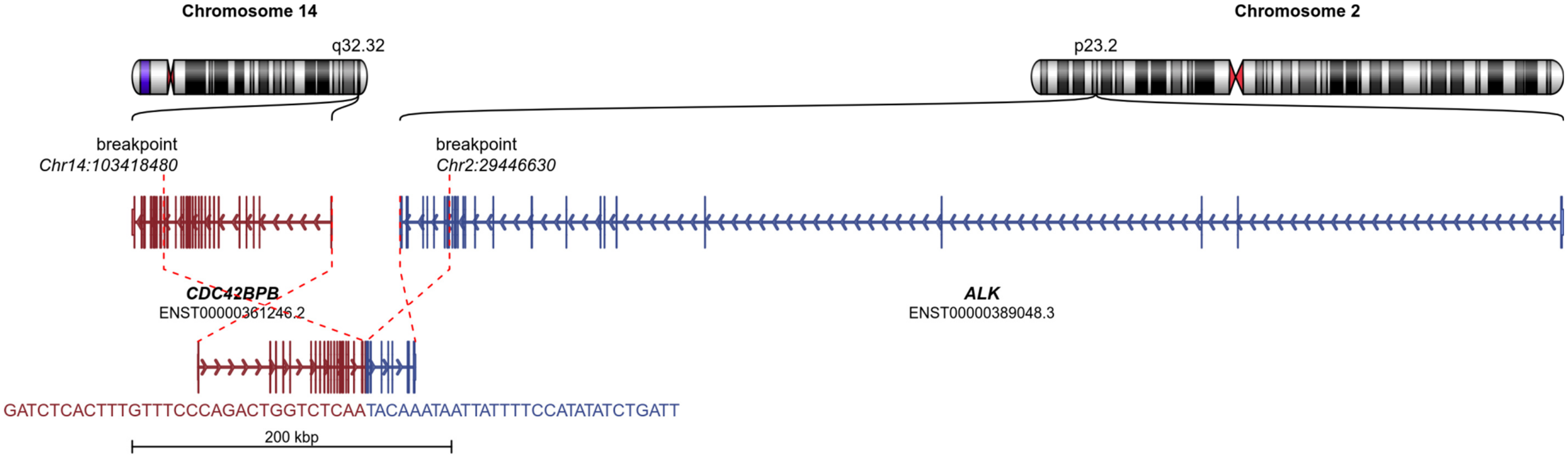

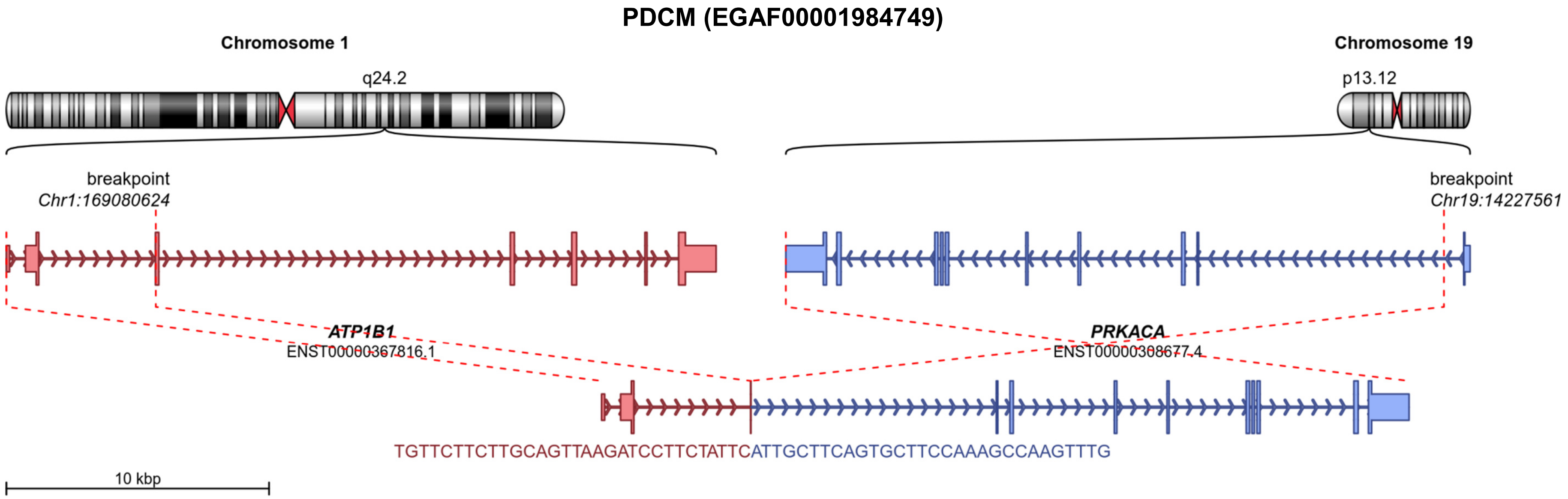

Supplemental Figure S7: Structural variants correlating with the putative driver fusions in Supplemental Figure S6.
Structural variants were identified by SOPHIA in matched whole-genome sequencing samples.
