# Supplementary material for: Accurate and efficient detection of gene fusions from RNA sequencing data
Source: Genome Res. 2021 Mar;31(3):448–60. doi: 10.1101/gr.257246.119 (PMC7919457; doi:10.1101/gr.257246.119)
Supplement: Supplemental Material [file supp_gr.257246.119_Supplemental_Figure_S8.pdf]

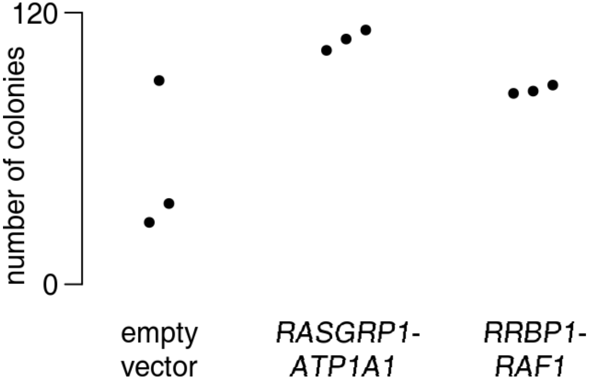

Supplemental Figure S8: Colony formation in soft agar of MCF10A cells transduced with fusion constructs.
$2{\times}10^4$ MCF10A cells were suspended in a top layer of RPMI-1640 containing 10 % fetal calf serum (FCS) and 0.35 % noble agar (Sigma) and plated on a bottom layer of RPMI-1640 containing 10 % FCS and 0.5 % soft agar in 6-well plates. The cells were additionally supplied with fresh growth medium supplemented with EGF every 3 days. After 6 weeks, colonies were stained and fixed with 0.05 % crystal violet in 20 % methanol. Colonies with a size bigger than 50 µm were imaged and quantified using the Lionheart FX automated microscope (BioTek).
