# Supplementary material for: Accurate and efficient detection of gene fusions from RNA sequencing data
Source: Genome Res. 2021 Mar;31(3):448–60. doi: 10.1101/gr.257246.119 (PMC7919457; doi:10.1101/gr.257246.119)
Supplement: Supplemental Material [file supp_gr.257246.119_Supplemental_Figure_S9.pdf]

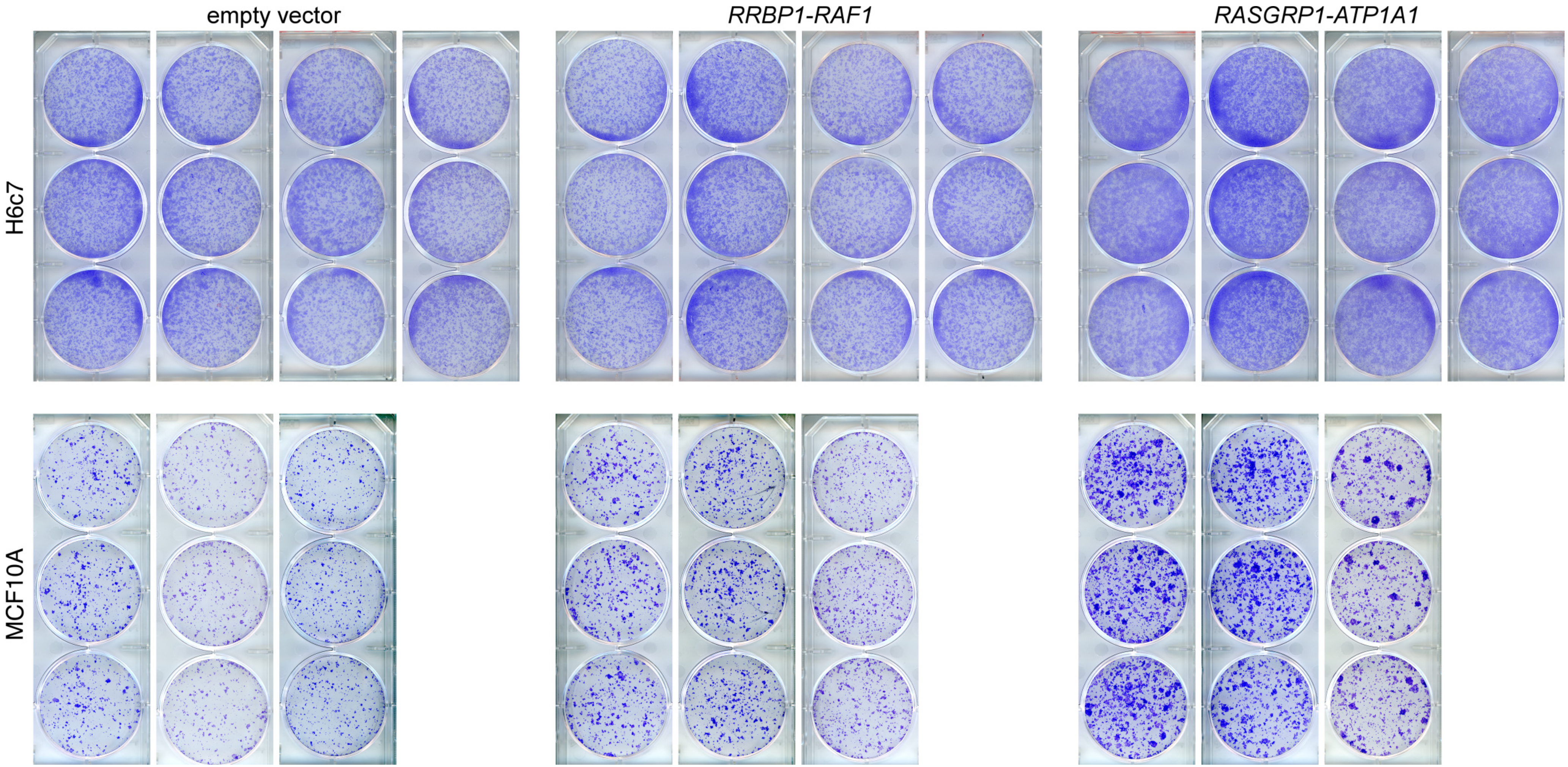

Supplemental Figure S9: Colony formation of H6c7 cells and MCF10A cells transduced with fusion constructs.
H6c7 and *TP53*-deficient MCF10A cells stably transduced with the fusions or empty vector were cultured for 7 days with EGF and 8 days without EGF, respectively.
