# Supplementary material for: Accurate and efficient detection of gene fusions from RNA sequencing data
Source: Genome Res. 2021 Mar;31(3):448–60. doi: 10.1101/gr.257246.119 (PMC7919457; doi:10.1101/gr.257246.119)
Supplement: Supplemental Material [file supp_gr.257246.119_Supplemental_Figure_S10.pdf]

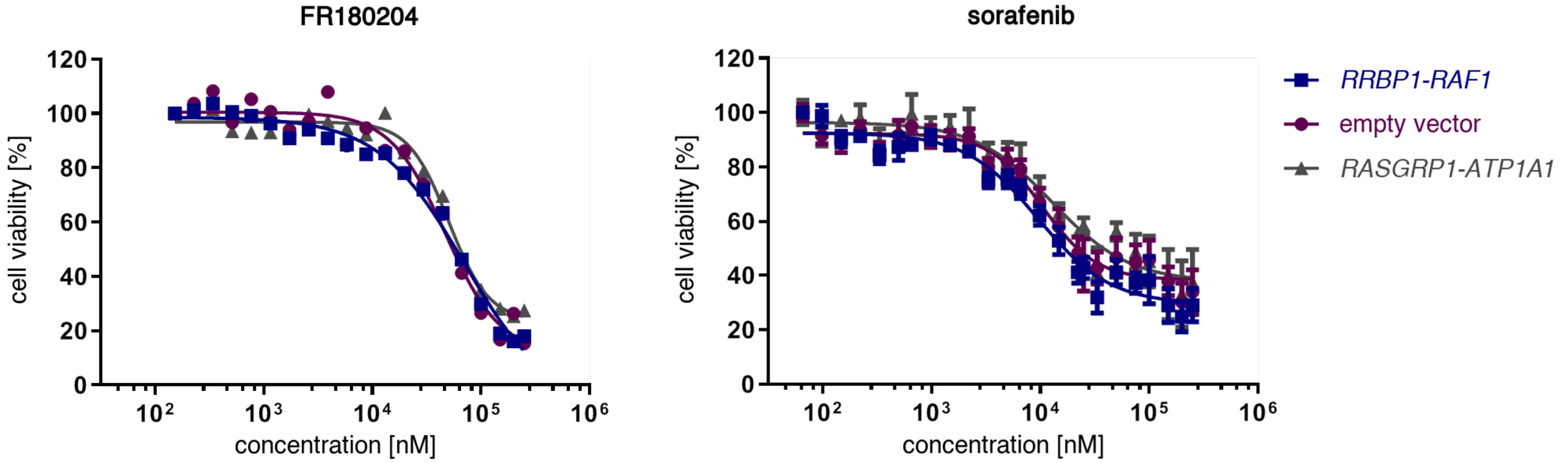

Supplemental Figure S10: Sensitivity of MCF10A cells to two compounds targeting the MAPK signaling pathway.
Treatment with the MAPK inhibitor FR180204 and the RAF1 inhibitor sorafenib over two days showed no significant effects in cells transduced with fusion constructs compared to empty vector controls. Cell viability was measured by MTS assay after 48 hours.
