# Supplementary material for: Accurate and efficient detection of gene fusions from RNA sequencing data
Source: Genome Res. 2021 Mar;31(3):448–60. doi: 10.1101/gr.257246.119 (PMC7919457; doi:10.1101/gr.257246.119)
Supplement: Supplemental Material [file supp_gr.257246.119_Supplemental_Figure_S11.pdf]

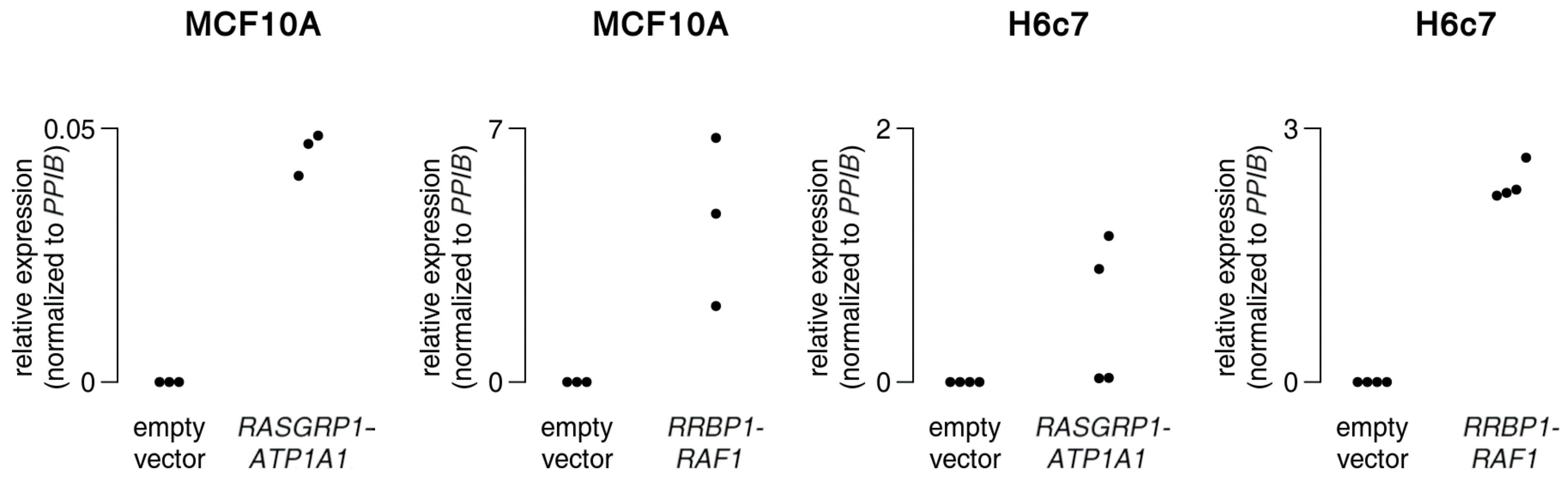

Supplemental Figure S11: mRNA expression of fusions measured by qPCR.
Expression of the fusions in MCF10A and H6c7 cells was determined by qPCR. The expression levels were normalized to the house-keeping gene *PPIB*.
