# Supplementary figures and images for: Accurate and efficient detection of gene fusions from RNA sequencing data
Source: Genome Res. 2021 Mar;31(3):448–60. doi: 10.1101/gr.257246.119 (PMC7919457; doi:10.1101/gr.257246.119)

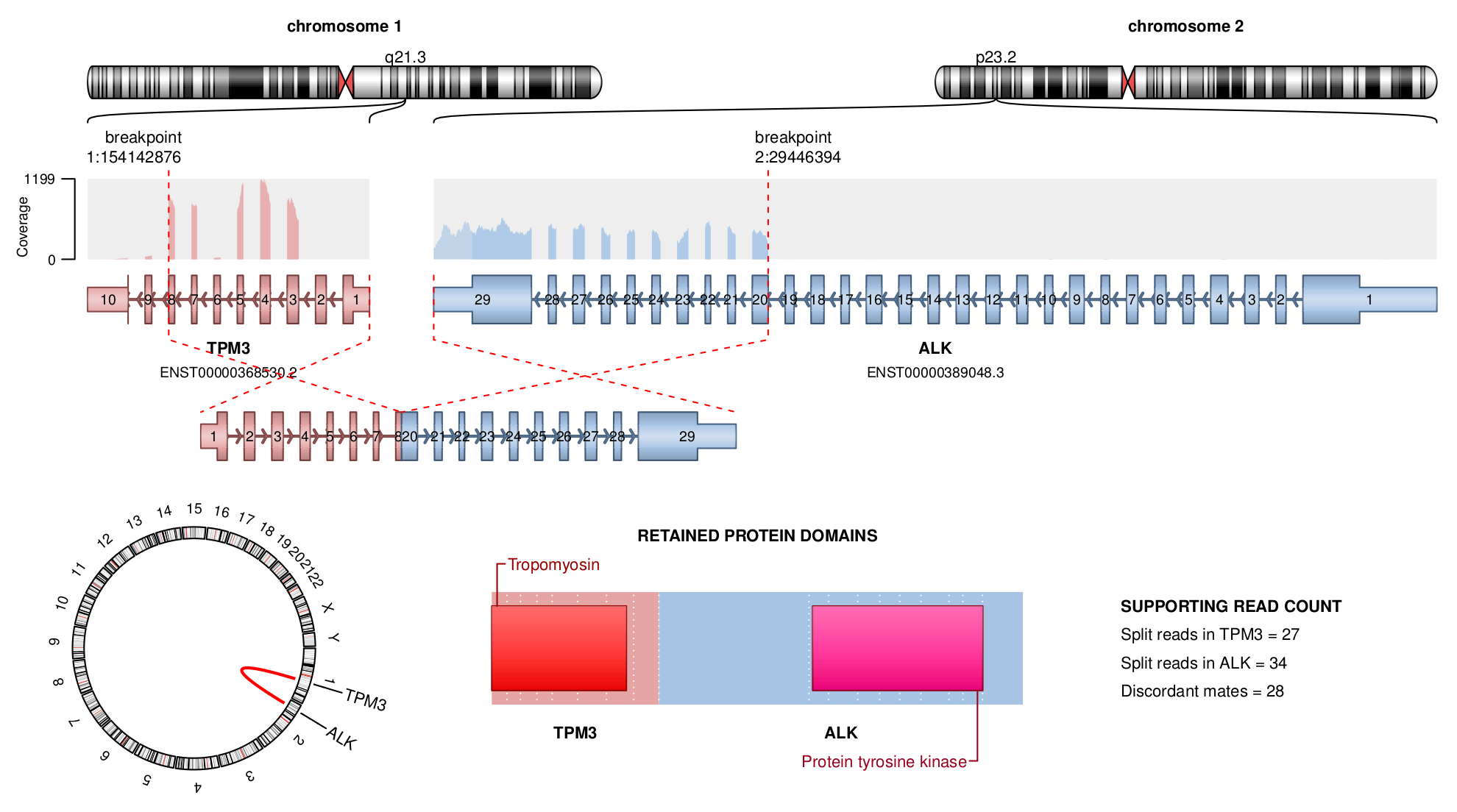

Supplement: Supplemental Material [file supp_gr.257246.119_Supplemental_Code_S1.zip › Supplemental_Code_S1/documentation/draw-fusions-example.png]

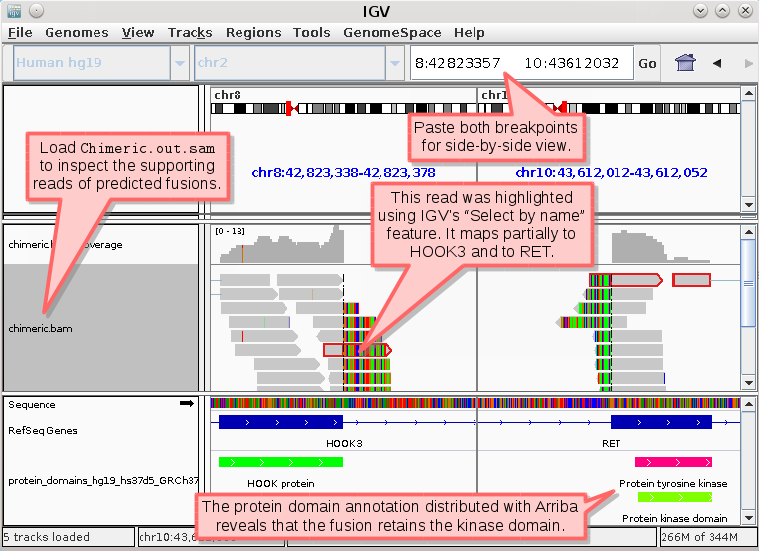

Supplement: Supplemental Material [file supp_gr.257246.119_Supplemental_Code_S1.zip › Supplemental_Code_S1/documentation/igv.png]

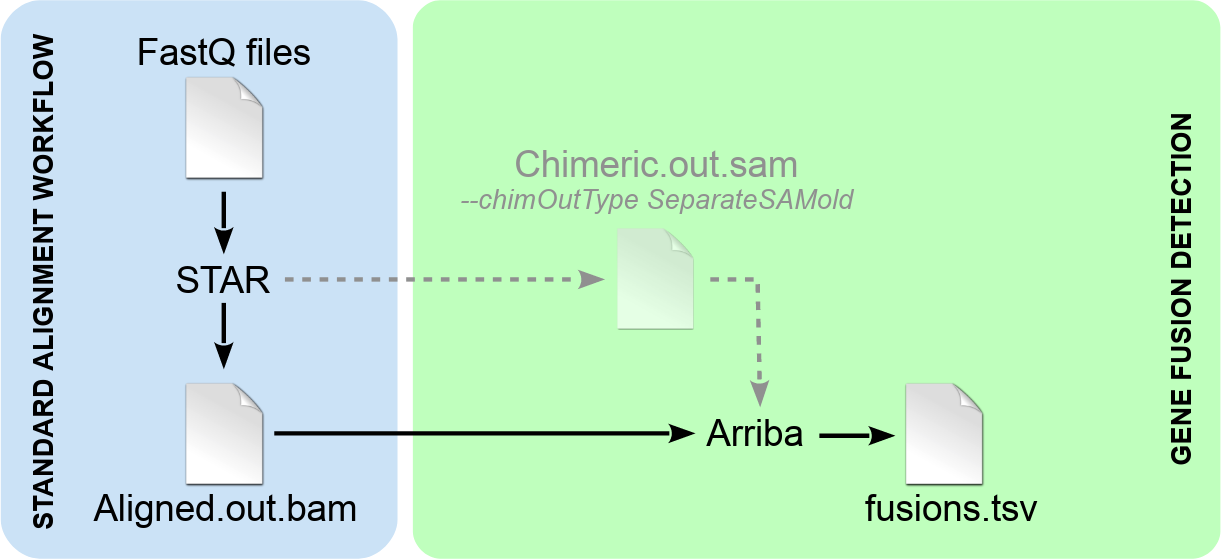

Supplement: Supplemental Material [file supp_gr.257246.119_Supplemental_Code_S1.zip › Supplemental_Code_S1/documentation/workflow.png]
